# Correlated Dual‐Gradient Electrodes Enabling Spatially Synchronized Sulfur Redox in High‐Mass‐Loading Li–S Batteries Under High Current Densities

**DOI:** 10.1002/adma.202517190

**Published:** 2025-12-26

**Authors:** Yuxuan Zhang, Yeongjun Oh, Jinwook Baek, Minyoung Kim, Zachary Didat, Han Wook Song, Sunghwan Lee

**Affiliations:** ^1^ School of Engineering Technology Purdue University West Lafayette Indiana USA; ^2^ Convergence Research Center for Meta‐Touch Korea Research Institute of Standards and Science (KRISS) Daejeon Republic of Korea

**Keywords:** 3D printing, correlated dual‐gradient electrode, high‐mass‐loading, Li–S, redox, spatial synchronized

## Abstract

The practical deployment of Li–S batteries is hindered by sluggish redox kinetics and poor ion transport in high‐mass‐loading sulfur cathodes, especially under fast‐charging and high‐power‐density conditions. Conventional electrocatalyst‐based strategies partially mitigate electrochemical polarization by lowering reaction energy barriers but fail to address concentration and ohmic polarization, which become more pronounced in thick electrodes. Here, a coupled material‐architecture approach is demonstrated by integrating electrocatalysts into a low‐tortuosity, correlated dual‐gradient electrode, fabricated via programmable high‐resolution stereolithography and pyrolysis‐induced carbonization. The microscale pore gradient is deliberately correlated with the active‐material gradient to spatially synchronize redox progression across electrode depth, thereby homogenizing cathode utilization and alleviating concentration polarization. Pyrolysis generates additional nanoscale pores, establishing a hierarchical structure and transforming polymer‐salt precursors into a conductive carbon framework embedding Li_2_S@Fe_2_O_3_/Fe‐N‐C, enhancing ion accessibility and minimizing ohmic polarization, while Fe_2_O_3_/Fe‐N‐C accelerates polysulfide conversion, reducing electrochemical polarization. Benefiting from this synergy, the Li_2_S@Fe_2_O_3_/Fe‐N‐C electrode delivers high‐areal‐capacities of 22.7 mAh cm^−2^ (1048 mAh g^−1^) at 0.1 C, 15.7 mAh cm^−2^ (725 mAh g^−1^) at 5 C, and retains 82% capacity over 1100 cycles at 4 C. A single‐layer pouch cell achieves a specific energy of 403 Wh kg^−1^, demonstrating the promise of this dual‐gradient strategy for real‐world high‐energy and high‐power Li–S batteries.

## Graphical abstract

Coupling a dual‐gradient carbonized framework with Fe_2_O_3_/Fe‐N‐C catalytic sites enables spatially synchronized sulfur redox across the entire electrode thickness in high‐mass‐loading Li–S batteries. This synergistic structural–catalytic design effectively mitigates concentration, ohmic, and electrochemical polarization, thereby achieving high‐capacity utilization rates under both high areal mass loading and fast‐charging conditions, advancing the practical realization of high‐energy‐ and high‐power‐density Li–S batteries.

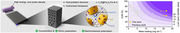

## 1 Introduction

Lithium‐sulfur (Li–S) batteries are recognized as one of the most promising next‐generation energy storage systems due to their high theoretical energy density (∼2600 Wh kg^−1^), low cost, and abundant resource availability [1, 2]. However, their practical application has been hindered by sluggish reaction kinetics, severe lithium polysulfides (LiPSs) shuttling effects, and low utilization of sulfur active materials, significantly limiting the achievable capacity and power density [3, 4]. To improve these reaction kinetics, extensive studies have focused on introducing electrocatalysts into sulfur cathodes. An ideal electrocatalyst for Li–S batteries should not only exhibit high catalytic activity but also demonstrate high thermodynamic stability and low material cost [5, 6].

**FIGURE 1:**
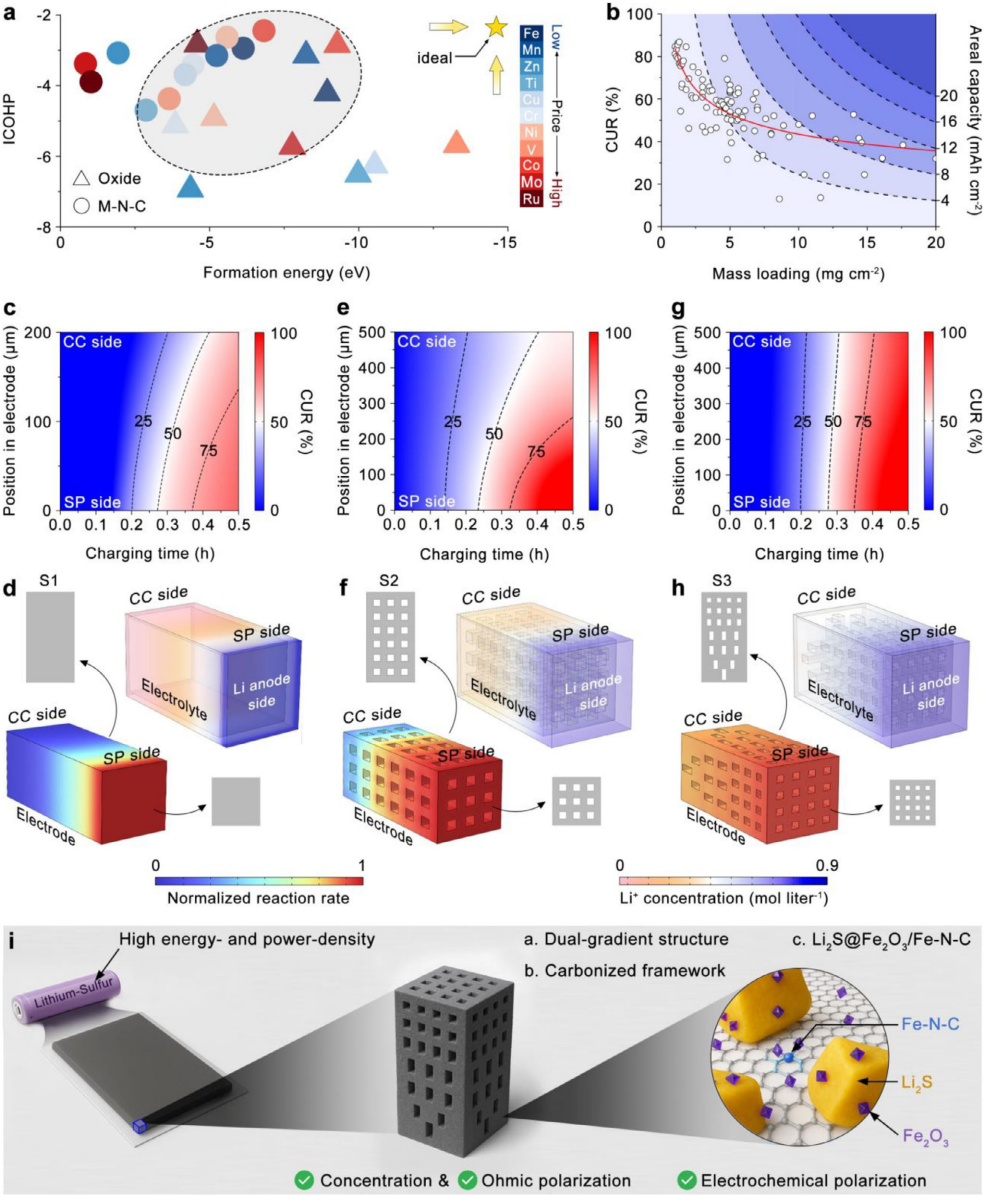
(a) Scatter plots of ICOHP values versus formation energies of representative metal oxides and M‐N‐C catalysts. (b) Statistical analysis of CUR at different mass loadings and the corresponding areal capacities (red line: fitted trend showing the decrease of CUR with increasing mass loading). (c, e, g) CUR distributions of the 2D planar, 3D uniform, and 3D dual‐gradient electrode structures along the electrode depth as a function of charging time. (d, f, h) Normalized reaction rate distributions (left‐bottom) and corresponding Li^+^ concentration profiles in the electrolyte (right‐top) for the 2D planar, 3D uniform, and 3D dual‐gradient electrode structures, respectively. (i) Schematic illustration of the synergistic effects between dual‐gradient structure and electrode material engineering.

To quantitatively assess the catalytic performance and stability of commonly used transition‐metal‐based metal–nitrogen–carbon (M–N–C) structures and metal oxides, we calculated the integrated crystal orbital Hamilton population (ICOHP) and formation energies [7, 8, 9, 10].ICOHP serves to evaluate catalytic activity by analyzing bonding interactions between active sites and adsorbed species, where less negative ICOHP values correspond to more favorable reaction kinetics. In parallel, formation energy provides insight into material thermodynamic stability, reflecting chemical structure robustness under electrochemical environments [11, 12]. These computational results (Figure 1a) indicate that catalysts based on Fe, Mn, Co, and Ru exhibit favorable catalytic activity and thermodynamic stability, aligning with the desired criteria for effective sulfur redox catalysis. Among these elements, Fe and Mn are especially attractive due to their significantly lower cost, eco‐friendliness, and absence of geopolitical mining concerns compared to Co and Ru [13, 14, 15, 16]. In recent studies, Fe‐ and Mn‐based catalysts have been extensively explored and confirmed to be effective for enhancing sulfur reaction kinetics. For instance, Ding et al. reported a biotemplated single‐atomic Fe‐N_2_ mediator that exhibited strong electrocatalytic activity toward dual‐directional sulfur conversion, delivering areal capacities of 1.56 mAh cm^−2^ at 1.2 mg cm^−2^ sulfur loading and 6.18 mAh cm^−2^ at 5.75 mg cm^−2^ sulfur loading [17]. Similarly, Zhang et al. designed a Fe_3_O_4_‐doped mesoporous carbon cubosome with a bicontinuous “plumber's nightmare” structure, which efficiently adsorbed and catalyzed polysulfide species, achieving areal capacities of 3.5 mAh cm^−2^ at 3.2 mg cm^−2^ and 6.5 mAh cm^−2^ at 8.2 mg cm^−2^ [18]. In addition to Fe‐based systems, Jiang et al. developed a cation/anion dual‐doped MnO_2_ catalyst that promotes sulfur redox by tuning the Mn‐O electronic structure, which delivered areal capacities of 1.48 mAh cm^−2^ at 1.2 mg cm^−2^ and 8.0 mAh cm^−2^ at 8.1 mg cm^−2^ [19]. Notably, even though these studies highlight the strong potential of Fe‐ and Mn‐based catalysts in enabling high‐performance Li–S batteries by simultaneously promoting sulfur redox kinetics and suppressing polysulfide shuttling, it has been observed across multiple studies that the areal capacity does not proportionally scale with increasing sulfur loading. Statistical analyses of data from over 100 recent publications (Figure 1b) reveal that strategies relying solely on material design for electrocatalytic enhancement lead to disproportionate scaling, which is directly related to a significant decrease in cathode utilization ratio (CUR) at high‐mass loadings, often falling below 40% [20]. Consequently, up to 60% of the active sulfur material remains electrochemically inactive during cycling, severely limiting achievable areal capacities beyond ∼16–20 mAh cm^−2^. This limitation significantly restricts battery energy densities, making it difficult to meet the high energy demands of long‐range electric vehicles, electric vertical take‐off and landing aircraft, drones, and humanoid robots [21, 22].

Moreover, most of the areal capacity values summarized in Figure 1b were obtained at relatively low current densities (≤ 0.5C) and are expected to decrease significantly at higher current densities, falling far short of meeting the fast‐charging and high‐power‐density requirements of practical devices [23, 24]. Therefore, in addition to material design, careful electrode structuring, particularly accounting for tortuosity, pore distribution, and active material distribution, is essential for enabling efficient ion transport and uniform redox kinetics [25, 26, 27]. Taking a representative two‐dimensional (2D) planar cathode (Structure 1, hereafter S1, thickness ∼200 µm shown in Figure S1a) as an example, we performed finite element analysis (FEA) to investigate active material utilization across the electrode depth under varying current densities. At a moderate current density (0.5 C, Figure S2a), the CUR near the separator side (SP side) was slightly higher than that near the current collector side (CC side) after 1 h charging. As the current increased to 2 C, the SP side reached over 75% CUR, whereas the CC side remained below 25% even at the end of charging, as indicated in Figure 1c, resulting in a low overall CUR (∼62%). At a higher current of 4 C, active materials from 70 to 200 µm depth showed utilization below 50% as shown in Figure S2b, further reducing CUR to ∼27%. It is observed that the reaction rate of the S1 electrode at the SP side is significantly higher than that at the CC side, leading to different utilization statuses of cathode active materials between the SP side and the CC side. As shown in Figure 1d, the Li^+^ transport was limited at the SP side of the electrode, leading to high concentration polarization along the electrode depth direction. This severe polarization in 2D planar cathodes originates from their inherently high tortuosity, where the distorted or non‐continuous ion channel in the electrode structure critically hinders efficient Li^+^ transport from the SP side to the CC side [28].

To alleviate this issue, reducing electrode tortuosity through engineered 3D structures has been proposed. For example, Han et al. demonstrated a low‐tortuosity “reinforced‐concrete type” cathode that preserved structural integrity and facilitated rapid ion transport, achieving a CUR of 30% at 2.5 C, which was more than twice that of a conventional 2D planar electrode under identical conditions [29]. Similarly, Wang et al. developed a free‐standing N, O co‐doped wood‐like carbon framework with vertically aligned microchannels, which delivered CURs of 51% at 0.5 C and 41% at 1 C with a sulfur loading of 17.3 mg cm^−2^, underscoring the critical role of reducing tortuosity in enhancing the CUR of high‐loading electrode [30]. To further understand the influence of low‐tortuosity design, we constructed a uniform 3D cathode (Structure 2, S2, 500 µm thick) featuring bidirectional through‐channels (horizontal and vertical) aligned along the electrode thickness by introducing a periodic lattice architecture, as shown in Figure S1b. Simulation results revealed reduced polarization of the S2 electrode at 0.5 C, illustrated in Figure S3a. At 2 C, CUR at the CC side reached above 50% higher than that in the S1 electrode, significantly increasing overall CUR to about 64% (Figure 1e). These results show that the uniform 3D structure substantially improves utilization at the CC side compared to traditional 2D electrodes due to the reduced tortuosity. Besides, the Li^+^ concentration gradients along the thickness direction have been decreased, resulting in a mitigated concentration polarization compared to the S1 electrode. However, there still existed a notable utilization difference between SP and CC sides as shown in Figure S3b, with the bottom (CC side) experiencing incomplete utilization of 25% at 4 C, lowering overall CUR (∼52%).

To further minimize polarization and balance utilization across electrode depth, we propose a novel low‐tortuosity dual‐gradient electrode (Structure 3, S3, as shown in Figure S1c). This design incorporates a correlated‐dual gradient architecture, in which the gradients of pore size and active material distribution are meticulously coupled to balance ion transport and redox kinetics across the electrode thickness. On the SP side, numerous smaller microscale‐pores are incorporated to create sufficient electrolyte pathways while avoiding excess electrolyte retention, thereby lowering the local Li^+^ concentration and mitigating concentration polarization. In parallel, a higher fraction of active material is intentionally allocated to this side, matching its intrinsically faster reaction rate. Conversely, on the CC side, the electrode features fewer but larger microscale‐pores, enabling greater local electrolyte storage and enhanced Li^+^ availability without sacrificing overall active material loading. Correspondingly, less active material is placed at the CC side to compensate for its slower reaction rate. This correlation between microscale pore‐size gradient and active‐material gradient defines the dual‐gradient nature of the design: the two gradients act synergistically to homogenize sulfur utilization across the electrode depth, effectively minimizing polarization and utilization imbalance between the SP and CC sides. As simulations demonstrated in Figure S3a, the S3 electrode exhibits nearly uniform active material utilization from SP to CC sides at 0.5 C. At a higher current density of 2 C, the difference in active material utilization between the SP side and CC side of the electrode is minimal, as shown in Figure 1g, with nearly 100% utilization achieved throughout the electrode thickness by the end of charging. Even at an elevated current density of 4 C, the active material utilization near the CC side remains above 75% as demonstrated in Figure S3b, resulting in an overall CUR as high as 87%. Analysis of the reaction rate distribution further demonstrates that the difference in reaction rates between the SP side and the CC side in the S3 electrode is significantly smaller compared to that observed in the S2 electrode. Moreover, the Li^+^ concentration gradient between the SP and CC sides is also substantially reduced in the S3 electrode relative to the S2 structure.

Based on comprehensive statistical comparisons and theoretical modeling, this study aims to establish practical manufacturing strategies for dual‐gradient electrode structures that effectively address the challenge of limited areal capacity at high mass loadings by mitigating concentration polarization and enabling spatially synchronized CUR along electrode depth. Although several approaches have been investigated for constructing graded electrodes, most reported demonstrations remain limited in practicality, particularly in terms of structural precision, scalability, and compatibility with diverse electrode chemistries. For instance, sequential blade‐casting with slurries of varying concentrations can generate graded structures; however, the electrodes fabricated by this method typically exhibit high tortuosity, and achieving multi‐gradient architectures requires repeated casting and drying steps, rendering the process complex and energy‐intensive [31]. Magnetic‐field‐assisted techniques have also been employed to align active materials and introduce compositional gradients, but their applicability is limited to magnetic species, with poor spatial precision and a narrow range of usable materials [32]. The template method, which relies on pre‐designed scaffolds to impose gradient architectures, also faces obstacles in fabricating high‐precision templates and in completely removing them after electrode formation without damaging the structure [33]. In addition, achieving practical high‐mass‐loading electrodes requires precise control over the size of microscale pores, since excessively large void spaces compromise sulfur loading and reduce areal capacity. Therefore, appropriately minimized voids are required to maintain mass loading while still facilitating ion transport. However, the scalable fabrication of highly customized dual‐gradient electrodes remains challenging due to inherent limitations in precision control in both microscale pore size and active material distribution, universality, and suitability for high‐mass‐loading electrodes.

Additive manufacturing offers significant advantages in terms of customizability, scalability, and compatibility with diverse materials. Despite its facile design and manufacturing capabilities, the practical application of additive manufacturing in battery electrode design remains limited by the resolution of the printed structures. Achieving high definition is particularly challenging for internal features within bulk electrodes, as uniform delivery of source materials and polymerization/curing energy is difficult to control. Consequently, nozzle‐based methods such as extrusion or inkjet printing typically yield feature sizes of 100–200 µm for external structures and a sub‐millimeter level (300–700 µm) for internal features (e.g., void space) due to the resolution constrained by nozzle diameters, which are generally larger than 100 µm [34, 35, 36].

In contrast, photopolymerization‐based 3D printing, such as stereolithography (SLA), uses photons to initiate monomer crosslinking. Here, the resolution is determined by the pixel size of the curing image pattern. Current SLA systems achieve external feature sizes of 20–40 µm and internal features of 70–80 µm with precise control over printing and curing parameters [37, 38]. More importantly, curing light can be patterned into millions of pixels using digital micromirror devices or liquid–crystal display technologies, enabling the simultaneous solidification of millions of features within seconds [39, 40]. This process is orders of magnitude faster than nozzle‐based methods and, therefore, more suitable for mass production and potential commercialization. We have recently demonstrated such sub‐100 µm 3D patterns for flexible sensor applications [41, 42].

Beyond the advantages of SLA, further shrinkage of micro‐size pore and enhancement of electronic conductivity were achieved in this study through pyrolysis. Pyrolysis not only induces volumetric shrinkage as polymeric precursors decompose, generating additional nanosized meso‐and macropores within the dual‐gradient architecture, but also transforms electrically insulating polymers into conductive carbon frameworks via pyrolytic carbonization [43, 44, 45]. Thus, a hierarchical pore structure is introduced, where SLA constructs a well‐defined micro‐scale pore gradient, while subsequent pyrolysis generates uniformly distributed nano‐sized pores on the electrode surface. This multi‐level hierarchy ensures both directional ion transport and enhanced interfacial accessibility.

Specifically, 1,3,5‐triallyl‐1,3,5‐triazine‐2,4,6‐trione (TTT) was selected as the polymer precursor due to its nitrogen‐containing functional groups and conjugated triazine‐based structure [46, 47, 48, 49]. After polymerization and carbonization, a conductive nitrogen‐doped carbon matrix forms, providing electronic conductivity to reduce ohmic polarization and additional active nitrogen‐defect sites for sulfur redox reactions. Li_2_SO_4_ was chosen as the sulfur precursor, reacting during carbonization to generate Li_2_S as the active cathode material. Additionally, as demonstrated by the computational analyses discussed above (Figure 1a), Fe‐based catalysts exhibit both high catalytic activity and excellent thermodynamic stability, alongside low material cost. Moreover, previous studies have reported that iron salts can react with carbon matrices at elevated temperatures, yielding various Fe‐based catalytically active species [50, 51]. Thus, Fe(NO_3_)_3_ was selected as the precursor material for catalysts to incorporate Fe species into the carbon framework of the electrode.

Benefiting from this rationally integrated material and structural design (Figure 1i), our S3 electrode demonstrated markedly enhanced electrochemical performance. It delivered a specific capacity of 980 mAh g^−1^ (21.2 mAh cm^−2^) at 0.5 C, and even at a high rate of 5 C retained 726 mAh g^−1^ (15.7 mAh cm^−2^). During long‐term cycling at 4 C, the electrode exhibited an initial capacity of 740 mAh g^−1^ (16.0 mAh cm^−2^) and maintained over 82.4% of its original capacity after 1100 cycles. Robust performance was also achieved under subzero conditions (−10°C), with a capacity of 638 mAh g^−1^ (13.8 mAh cm^−2^) at 1 C, highlighting the electrode's resilience under extreme operating environments. Importantly, the Li‐containing dual‐gradient cathode can be paired with a Si/C anode to circumvent the challenges associated with metallic lithium. The resulting S3||Si/C full cell delivered 774 mAh g^−1^ (16.7 mAh cm^−2^) at 3 C and retained 94.1% of its capacity after 100 cycles. Furthermore, a single pouch cell assembled with the S3 electrode achieved an energy density of 403 Wh kg^−1^ at 0.05 C, maintaining 89.7% of its capacity after 50 cycles under 0.5 C, underscoring the strong potential of the S3 design for practical high‐energy and high‐power applications. In situ characterizations coupled with simulations elucidated clear kinetic differences during sulfur evolution reactions (SER)/sulfur reduction reactions (SRR) among 2D planar (S1), uniform 3D (S2), and dual‐gradient (S3) electrodes. Mechano‐electrochemical coupling simulations further explained why structural pulverization occurs in S2, whereas S3 maintains structural integrity after cycling. In conclusion, this study presents a rational electrode design strategy enabling high areal capacity and utilization at high rates, providing a promising route toward high‐energy‐ and high‐power‐density Li–S batteries.

## 2 Result and Discussion

The synthetic procedures for the S1, S2, and S3 electrodes are schematically illustrated in Figure S5. Initially, the electrode “green body” was fabricated using a precursor solution containing lithium sulfate and ferric nitrate, as detailed in the Experimental Section. After undergoing pyrolysis, the “green body” was converted into a carbonized “brown body” with a robust conductive skeleton. The resulting carbonized electrodes exhibited an electronic conductivity of 6.7 S cm^−1^ (Figure S6), which is sufficiently high to ensure efficient electron transport even under high charge–discharge rates. To further suppress the shuttling of LiPSs, the brown body electrodes were conformally coated with a thin o‐PEDOT layer via oxidative chemical vapor deposition, following our previously reported protocols [52, 53]. Notably, while all electrodes were composed of the same material components, they were engineered with distinct structural architectures to systematically investigate how electrode architecture influences Li–S conversion kinetics in high‐mass‐loading electrodes.

**FIGURE 2:**
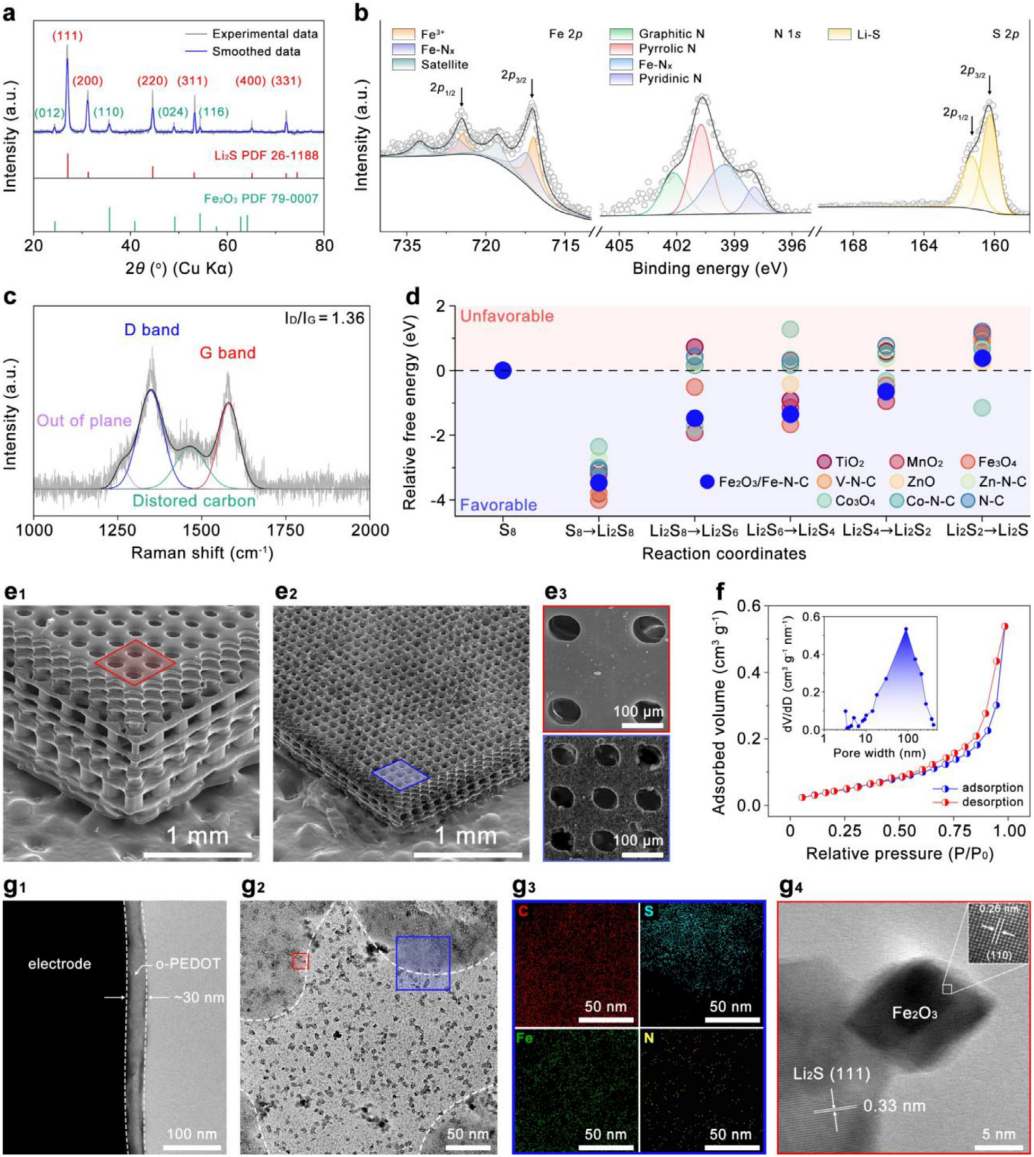
(a) XRD patterns of electrode materials after pyrolysis. (b) High‐resolution XPS spectra of Fe 2p, N 1s, and S 2p binding energy regions of the electrode materials after pyrolysis. (c) Raman spectra of carbon matrix. (d) Relative free energy profiles of typical oxide and M‐N‐C materials and Fe_2_O_3_/Fe‐N‐C in terms of reaction coordinates. (e) SEM images of the S3 electrode before and after pyrolysis. (f) N_2_ adsorption‐desorption isotherms and corresponding pore size distribution of the electrode after pyrolysis. (g) TEM images of the pyrolyzed electrode. (g1) conformal o‐PEDOT coating layer; (g2) TEM image of the electrode material after pyrolysis; (g3) EDX elemental mapping of the selected region in (g2) (blue square); (g4) HR‐TEM image of the selected region in (g2) (red square). Inset: lattice fringe image of the selected white square in Figure 2g4.

The crystalline structures of the electrode materials were characterized by X‐ray diffraction (XRD), as shown in Figure 2a. Diffraction peaks located at 2θ values of 26.9, 31.3, 44.6, 53.3, 65.1, and 72.1 can be assigned to the (111), (200), (220), (311), (400), and (331) planes of Li_2_S. The formation of Li_2_S as the active material in the electrode is attributed to the redox reaction between Li_2_SO_4_ and the carbonized poly‐(TTT). In addition, weak peaks at 2θ values of 24.2, 35.6, 49.1, and 54.4 correspond to the (012), (110), (024), and (116) planes of Fe_2_O_3_, which results from the thermal decomposition of Fe(NO_3_)_3_ during the synthetic pyrolysis process. The presence of Fe_2_O_3_ verifies the successful incorporation of catalytic species, which are expected to facilitate polysulfide adsorption and reduce the energy barriers for LiPSs conversion. The chemical interactions and bonding environment of the electrode materials were further analyzed by X‐ray photoelectron spectroscopy (XPS).

As shown in Figure 2b, two prominent spin‐orbit splitting peaks at 711.3 and 724.6 eV can be assigned to Fe 2p_3/2_ and Fe 2p_1/2_, respectively. The binding energies of these peaks are attributed to the highly spin‐paired electron in Fe components, which corresponds to the presence of the weak‐field ligand nature of O^2−^ in the Fe‐O coordination environment. In addition, characteristic satellite peaks appear at 732.6 and 717.9 eV, which are attributed to the strong electron‐electron interactions between Fe 3d and O 2p states, confirming the formation of Fe_2_O_3_ [54]. Notably, the leftward skew of the Fe 2p_3/2_ and Fe 2p_1/2_ peaks is ascribed to the altered chemical environment, arising from interactions between Fe and coordinated anions (such as C, N, or S) [54, 55]. These heteroatom ligands influence the local electronic environment of Fe, leading to partial electron withdrawal and thus elevating the binding energy of a fraction of Fe 2p states. Subsequent high‐resolution XPS analysis of C, N, and S species confirms that these two component peaks originate from Fe—N interactions. The high‐resolution Fe 2p and N 1s XPS spectra were further analyzed to elucidate the coordination environment of Fe species. According to the XPS‐derived atomic composition, Fe and N account for 2.9 at.% and 12.4 at.% of the total sample, respectively. The deconvolution of the Fe 2p spectrum reveals that approximately 42% of Fe exists in Fe—N_x_ coordination states, while the remaining 58% corresponds to Fe—O species. Therefore, the atomic fraction of Fe bonded with N is estimated to be 2.9 × 42% = 1.21%. In the N 1s spectrum, four distinct components are identified as pyridinic N (19.5%), pyrrolic N (36.1%), Fe—N_x_ (35.7%), and graphitic N (8.7%), indicating that 35.7% of the nitrogen atoms are coordinated with Fe. Thus, the atomic fraction of N involved in Fe—N_x_ coordination is calculated as 12.4 × 35.7% = 4.43%. Consequently, the relative atomic ratio between Fe and N participating in Fe—N_x_ bonding is approximately 1.21:4.43, corresponding to a Fe:N atomic ratio of about 1:3.6. However, XPS alone cannot directly determine the coordination number of Fe—N species. To further clarify the local structure, DFT calculations were conducted to evaluate the thermodynamic stability of Fe—N complexes with different coordination numbers anchored on a single‐layer carbon substrate as shown in Figure S8. The results reveal that the Fe—N_4_ configuration possesses the lowest formation energy (−1.1 eV), indicating that the Fe‐N_x_ complex is most likely coordinated in an Fe—N_4_ configuration. The N 1s high‐resolution spectrum exhibits a broad and asymmetric peak, indicating the coexistence of multiple nitrogen coordination environments with distinct binding energies. Deconvolution of the N 1s peak reveals four components located at 397.9, 399.7, 400.5, and 401.4 eV. The gradual increase in binding energy reflects the progressive reduction in electron density around the nitrogen atoms as their bonding environments shift from localized lone‐pair states to more delocalized π‐conjugated systems. Accordingly, the four component peaks can be assigned to pyridinic N, pyrrolic N, metal‐coordinated N, and graphitic N, respectively [56]. In particular, the component at 400.5 eV is attributed to metal‐coordinated nitrogen, predominantly arising from Fe—N bonding rather than Li—N, because Fe exhibits a much stronger orbital hybridization with N 2p states and a higher electronegativity difference compared to Li [57, 58]. This stronger coordination leads to greater electron withdrawal from nitrogen, consistent with the higher binding energy observed. These nitrogen defects are known to enhance the redox kinetics of sulfur species by facilitating electron transfer and providing active sites for LiPSs adsorption and conversion [59]. The S 2p spectrum of elemental sulfur (S_8_) typically exhibits an S 2p doublet at ∼164.0–164.2 eV (2p_3/2_) and ∼165.2–165.4 eV (2p_1/2_) with a characteristic spin‐orbit splitting of ∼1.18 eV and a 2:1 area ratio [60]. However, the dominant doublet peaks of the pyrolyzed specimen are observed at 161.2 and 160.3 eV, far below the elemental sulfur region, confirming that their signals originate from sulfur bound in a reduced, metal‐sulfur environment, rather than elemental S_8_ [61, 62, 63]. Since the S 2p3/2 peak of Fe‐S species typically appears at a slightly higher binding energy (about 162–163 eV) due to the higher electronegativity of Fe [63, 64], the doublet peaks observed at 161.2 and 160.3 eV are more likely attributed to Li—S interactions. In Li–S binding, electrons donated from Li 1s to S 3p orbitals increase the local electron density around sulfur, consequently, lowering the binding energies of the S 2p_3/2_ and S 2p_1/2_ core levels. In addition, the C 1s spectrum (Figure S7) displays deconvoluted peaks located at 284.5 and 285.8 eV, which correspond to C‐C and C‐N bonding states, confirming the graphitic carbon framework and heteroatom doping [65, 66]. Crucially, no signal is detected in the 283–284 eV region, where Fe—C bonds are typically found due to Fe 3d‐C 2p hybridization [55, 67]. The absence of this low‐binding‐energy feature rules out Fe—C coordination. Taken together, the combined Fe 2p, N 1s, and C 1s analyses confirm that Fe is incorporated as Fe—N and Fe—O species within the carbon framework, with no evidence for Fe—S or Fe─C bonding. To further probe the structural characteristics of the carbon matrix, Raman spectroscopy was conducted both before (Figure S9) and after pyrolysis (Figure 2c) to analyze the degree of graphitization and structural disorder in the electrode. Prior to pyrolysis, the spectrum of the polymeric precursor displays broad features characteristic of organic backbones and triazine‐derived vibrations, without distinct D or G bands, reflecting the absence of a graphitized carbon framework. After pyrolysis, two prominent peaks appear at ∼1350 and ∼1580 cm^−1^, corresponding to the D‐ and G‐bands of carbon, respectively. The D‐band at ∼1350 cm^−1^ arises from the breathing mode of sp^2^ carbon rings and is activated by structural disorder, while the G‐band at ∼1580 cm^−1^ originates from the in‐plane stretching of sp^2^ carbon networks, reflecting graphitic ordering. The relative intensities of the two bands therefore provide a reliable measure of the degree of disorder and defect density within the carbon framework [59, 68]. The calculated I_D_/I_G_ ratio of 1.36 suggests a moderate degree of disorder with comparable contributions from amorphous and graphitic domains, which provides both electrical conductivity and abundant defect sites for polysulfide adsorption. In addition, a shoulder peak observed at ∼1260 cm^−1^ is attributed to out‐of‐plane carbon vibrations, indicating the presence of heteroatom doping and defects within the carbon framework, likely due to nitrogen incorporation and the partial disruption of graphitic domains by Fe species during pyrolysis. Another peak located at ∼1460 cm^−1^ is assigned to distorted carbon structures, further evidencing defect‐induced disorder. Collectively, these Raman results confirm that pyrolysis transforms the insulating polymer precursor into a conductive, defect‐rich carbon matrix, providing efficient electron pathways and abundant active sites. Within this matrix, Li_2_S and Fe‐based catalytic species generated during pyrolysis are uniformly embedded, ensuring intimate interfacial contact that enhances overall redox kinetics.

To evaluate the electrocatalytic role of Fe_2_O_3_/Fe‐N‐C species in sulfur conversion reactions, comparative experiments were performed using a Li_2_S/C electrode and a Li_2_S@Fe_2_O_3_/Fe‐N‐C electrode. The fabrication procedures for both electrodes are described in the Experimental Section. As shown in Figure S10, the charge–discharge profile of the Li_2_S@Fe_2_O_3_/Fe‐N‐C electrode at 1 C displays a significantly lower overpotential than that of the Li_2_S/C electrode, indicating a reduced energy barrier for Li_2_S oxidation and thus enhanced sulfur evolution reaction (SER) kinetics. During discharge, the Q_L_/Q_H_ (defined as the capacity contribution from the low‐voltage plateau relative to that from the high‐voltage plateau) ratio of the Li_2_S@Fe_2_O_3_/Fe‐N‐C electrode reaches 2.75, notably higher than the 1.82 observed for the Li_2_S/C electrode, suggesting superior catalytic activity toward sulfur reduction reactions (SRR). In addition, the voltage gap of the Li_2_S/Fe_2_O_3_/Fe‐N‐C electrode appears at approximately 0.21 V, lower than the 0.42 V of the Li_2_S/C electrode, further evidencing facilitated LiPSs conversion. These results demonstrate that Fe_2_O_3_/Fe‐N‐C species effectively promote both the SER and SRR processes by lowering the reaction energy barriers and improving redox reversibility. To further quantify these catalytic effects, density functional theory (DFT) calculations were performed to compare the relative free energies of typical oxide catalysts, M‐N‐C catalysts, and Fe_2_O_3_/Fe‐N‐C (Figure 2d; Figure S11 and Table S2). The results reveal that Fe_2_O_3_/Fe‐N‐C exhibits lower free energy barriers across most reaction coordinates compared with most of the benchmark catalysts, particularly for the critical Li_2_S_2_ to Li_2_S conversion step, where it shows the lowest relative energy among all tested catalysts [69, 70]. This confirms that Fe_2_O_3_ primarily acts as a solid catalytic center that facilitates the conversion of medium‐ to short‐chain LiPSs while the Fe‐N‐C provides favorable kinetics for the conversion from Li_2_S_8_ to Li_2_S_6_ and the precipitation of Li_2_S, underscoring its superior electrocatalytic capability in accelerating LiPSs conversion.

In addition to compositional characteristics, the structural features of the electrodes were examined by scanning electron microscopy (SEM). As shown in Figure 2e1, the as‐fabricated S3 electrode “green body” exhibits a designed dual‐gradient structure, characterized by a decreasing microscale pore size toward the upper direction, as schematically illustrated in Figure S12. This unique feature was enabled by highly customized SLA‐based manufacturing. For comparison, the SEM image of the S2 electrode (Figure S12a) reveals uniformly distributed micrometer‐pores and channels of nearly identical size, as expected from its 3D uniform design. Both S2 and S3 “green body” electrodes display the smooth surface morphology typical of SLA‐manufactured parts. After pyrolysis, the structural frameworks of both S2 and S3 were well preserved, exhibiting only a shrinkage in electrode dimensions, as shown in Figure 2e2 and Figure S12b. The cross‐sectional images (Figure S13) further reveal that the thicknesses of the S2 and S3 electrodes are approximately 490 µm. Moreover, the S2 electrode displays uniformly arranged and regularly distributed pores, whereas the S3 electrode exhibits a clear dual‐gradient pore‐size distribution across its thickness. Notably, the pyrolyzed electrodes exhibit a much rougher surface texture. To further evaluate this change, top‐view SEM images of the S3 electrode before and after pyrolysis were compared (Figure 2e3). The surface of the electrode before pyrolysis (red box) is relatively smooth with flat microscale‐pore walls. After pyrolysis (blue box), the surface becomes significantly roughened, with numerous nanoscale protrusions and depressions distributed along the micrometer pore walls. This increased surface roughness is likely associated with gas release and structural reconstruction during pyrolysis, which may contribute to enhanced interfacial contact with electrolyte and provide additional active sites for electrochemical reactions. Specifically, the thermal decomposition and carbonization of poly‐(TTT) release gaseous byproducts, while the reaction between Li_2_SO_4_ and carbon further contributes to gas evolution. Moreover, the transformation of Fe(NO_3_)_3_ in the presence of carbon to form Fe‐N‐C species is also accompanied by gas release, which collectively contributes to the development of additional pyrolytic micro‐sized pores within the electrode over the primary microscale porous framework.

**FIGURE 3:**
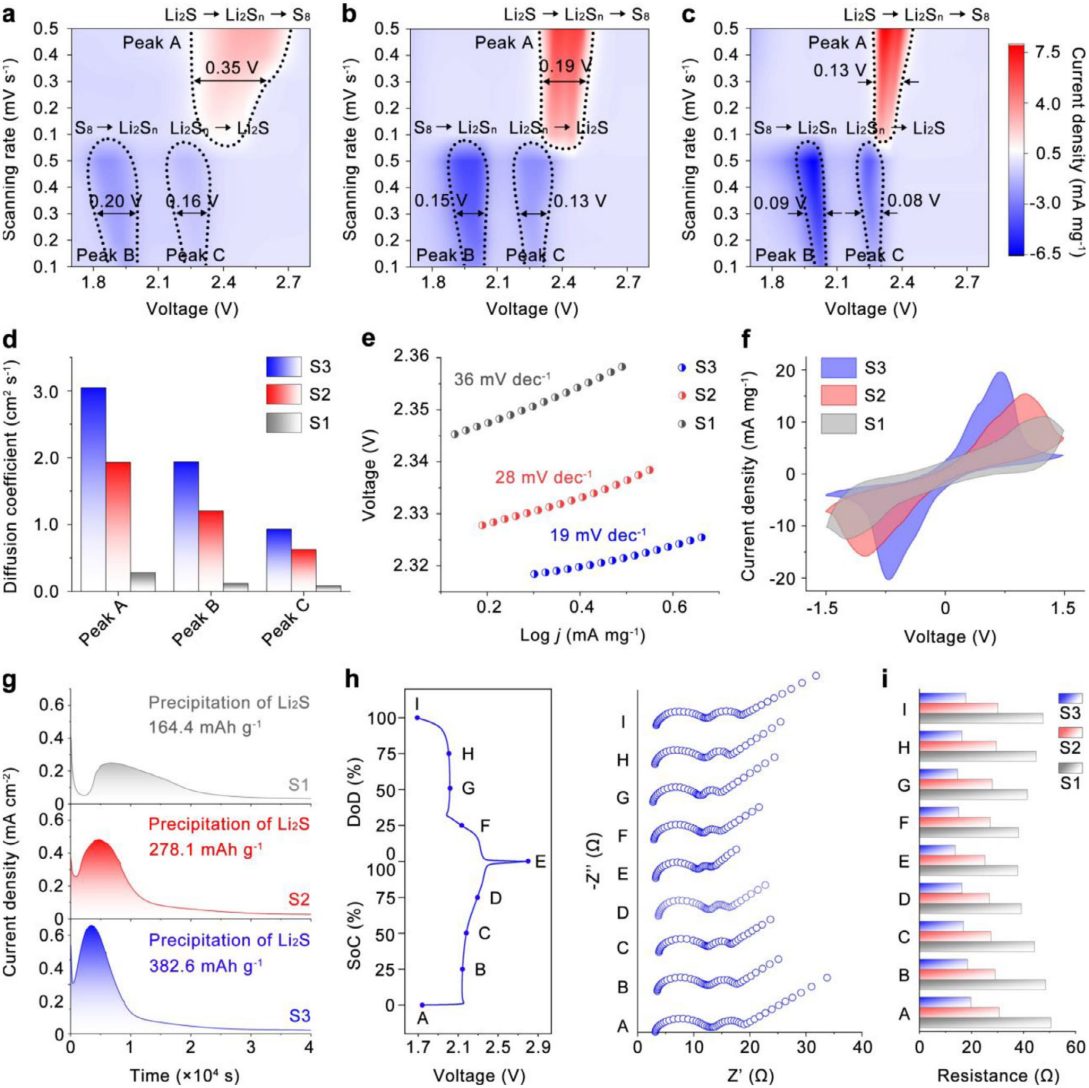
Contour maps for the CV curves of (a) S1 electrode, (b) S2 electrode, and S3 electrode (0.1 ‐ 0.5 mV s^−1^). (d) Chemical diffusion coefficients of Li^+^ of S1, S2, and S3 electrodes at peak A, B, and C. (e) Corresponding Tafel plots of peak A of CV curves. (f) CV curves of symmetric batteries at a scan rate of 5 mV s^−1^. (g) Identification of Li_2_S precipitation on S1, S2, and S3 electrodes through potentiostatic discharge measurements. (h) In situ EIS analysis of the S3 electrode and corresponding voltage profile during the second cycle. (i) Histograms of S1, S2, and S3 electrode obtained from the in situ EIS at different states.

Brunauer‐Emmett‐Teller (BET) analysis was employed to further quantify the additionally generated porous characteristics during the pyrolysis of the electrode materials. The N_2_ adsorption‐desorption isotherms are shown in Figure 2f, with the corresponding pore size distribution curves presented in the inset. According to the International Union of Pure and Applied Chemistry (commonly IUPAC) classification, the isotherms of the electrode materials are categorized as type IV, which indicates the presence of nanoscale mesoporous (typically 2–50 nm) and macroporous (> 50 nm) structures with capillary condensation behavior [71]. The clear hysteresis loop, where the desorption branch lies above the adsorption branch, is identified as a typical H_3_‐type hysteresis loop, which is characteristic of slit‐shaped nanopores formed by particle aggregation [72]. The presence of this hysteresis loop confirms the development of mesoporous structures within the electrode materials. Based on BET fitting (Figure S15), the specific surface area of the electrode materials was determined to be 35.65 m^2^ g^−1^, which provides (i) a continuous interface that facilitates Li–S conversion reactions with the low‐tortuosity design, (ii) internal voids to buffer the large volume change, and (iii) polysulfide adsorption. These nanopores are primarily generated during pyrolysis as a result of precursor decomposition and associated gas release, and structural shrinkage.

The nanopore size distribution (inset of Figure 2f) reveals that the nanopores are predominantly larger than 10 nm, with negligible micropores (< 2 nm). The distribution extends from the mesopore to the macropore range, with an average nanopore diameter of 133.18 nm. Such a hierarchical nanoscale meso‐/macroporous structure, combining a designed micrometer‐scale pore‐gradient, synergistically enhances electrolyte infiltration and accelerates Li^+^ transport, thereby supporting efficient sulfur redox reactions in thick electrodes

Transmission electron microscopy (TEM) analysis was conducted to elucidate the microstructural features of the electrode materials. As shown in Figure 2g1, an amorphous layer with a thickness of approximately 30 nm is closely attached to the electrode surface, confirming the conformal o‐PEDOT coating. The bulk structure of the electrode, presented in a plan‐view TEM image (Figure 2g2), reveals darker contrast regions near the four corners of the image compared to the central area, accompanied by numerous black nanoparticles with diameters of ∼5–10 nm dispersed throughout the matrix. To further identify the chemical composition of these regions, EDX mapping was performed at the boundary between a dark and light region marked by a blue square in Figure 2g2. The mapping results show a uniform distribution of carbon across the entire area, as shown in Figure 2g3, consistent with the presence of Li_2_S and Fe_2_O_3_/Fe‐N‐C embedded within the carbon matrix, which provides both electrical conductivity and anchoring sites for stable participation in redox reactions. Sulfur is predominantly localized in the darker regions, suggesting that these areas correspond to Li_2_S. Meanwhile, Fe and N signals are homogeneously distributed across the electrode, confirming the uniform dispersion of Fe_2_O_3_/Fe‐N‐C species. In this region, partial overlaps between Fe‐N‐C domains and Li_2_S particles can be observed, suggesting direct interfacial contact between them. During charging, the solid Li_2_S gradually converts into soluble LiPSs, which readily migrate through the electrolyte to reach the Fe‐N‐C sites. The atomically dispersed Fe‐N active centers serve as abundant catalytic sites for liquid‐phase redox reactions of LiPS intermediates, accelerating their conversion kinetics and suppressing shuttle effects. The black nanoparticles are most likely Fe_2_O_3_ due to the pronounced crystallinity of Fe_2_O_3_. To verify this, HR‐TEM analysis was conducted on a region marked with a red box in Figure 2g2. As shown in Figure 2g4, the nanoparticle displays the characteristic octahedral geometry, which is the typical shape of Fe_2_O_3_ single crystal, corroborating the previous conclusion. Moreover, the inset of Figure 2g4 displays distinct lattice fringes with an interplanar spacing of 0.26 nm, corresponding to the (110) facet of Fe_2_O_3_, further confirming that the octahedral nanoparticle is Fe_2_O_3_. In addition, lattice fringes with an interplanar spacing of approximately 0.33 nm are also observed, corresponding to the (111) plane of Li_2_S, further confirming that the dark‐contrast regions in Figure 2g2 are composed of Li_2_S. Thermogravimetric analysis (TGA) was employed to quantify the sulfur loading of the electrodes. The measurements were conducted in air, with the temperature ramped from room temperature to 800°C at a heating rate of 5°C min^−1^, and the resulting TGA curves are presented in Figure S16. As shown, when the temperature exceeded 750°C, the mass of the electrodes without Li_2_S and those containing Li_2_S decreased by 89.8% and 16.0%, respectively. Beyond this temperature, no further mass loss was observed. Based on the weight changes, the mass ratio of Li_2_S in the electrode is calculated to be approximately 30.8%, while Fe_2_O_3_/Fe‐N‐C accounts for about 10.2%, and the carbon matrix comprises 59% of the total electrode mass.

**FIGURE 4:**
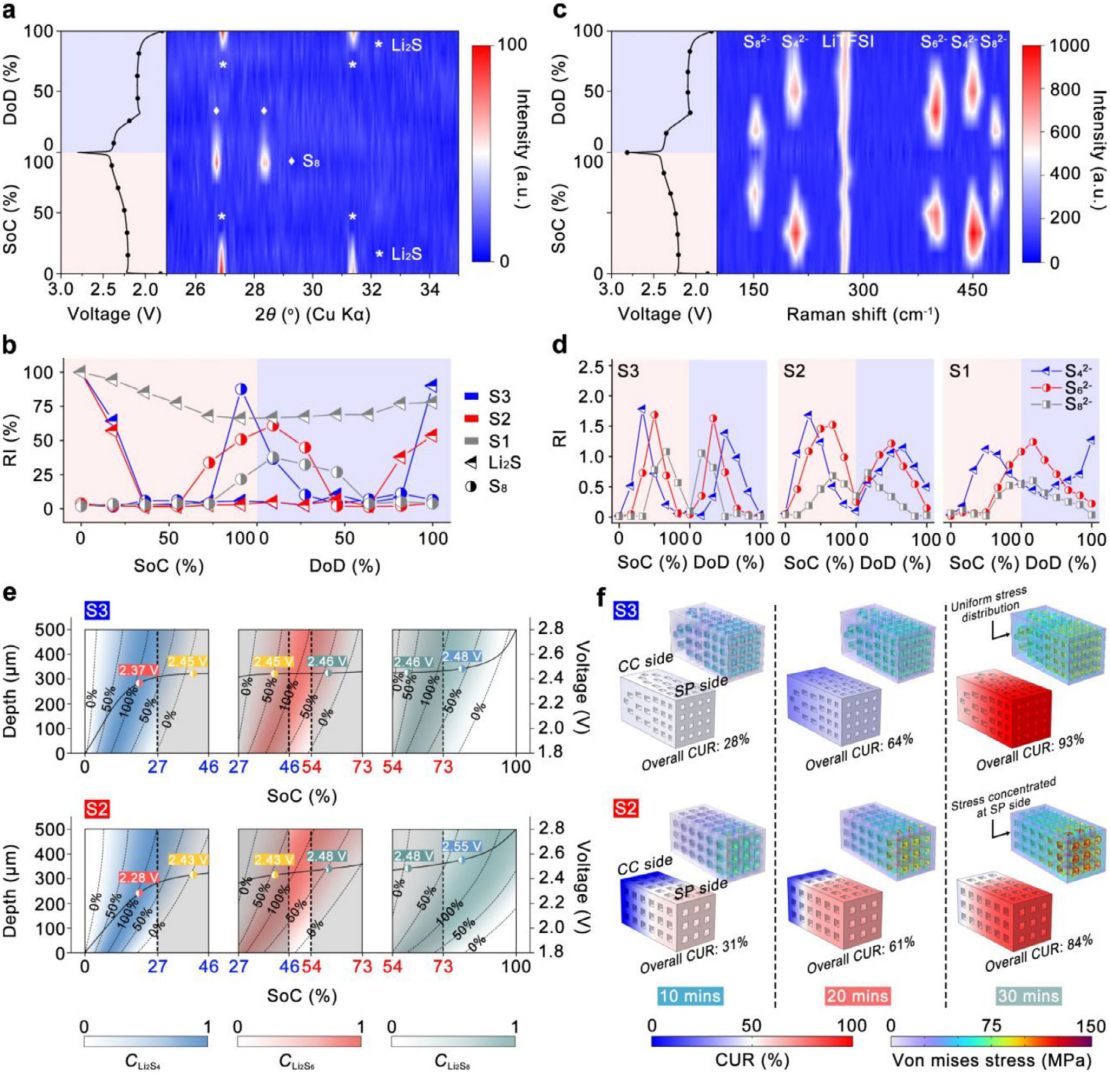
(a) The contour plot of the XRD patterns for a Li–S cell with the S3 electrode, with the corresponding voltage profiles. (b) The normalized diffraction peak intensity showing the evolution of crystalline S_8_ and Li_2_S of the S3 electrode. (c) The contour plot of the Raman spectrum for a Li–S cell with the S3 electrode, with the corresponding voltage profiles. (d) The normalized Raman peak intensity showing the evolution of Li_2_S_8_, Li_2_S_6_, and Li_2_S_4_ of the S3 electrode. (e) The LiPSs species distribution in the S3 (top) and S2 (bottom) electrode from 0% to 100% SoC (0 µm: SP side, 500 µm: CC side). (f) Simulation of the cathode utilization evolution and von Misses stress distribution in the S3 (top) and S2 (bottom) electrodes after charging 10, 20, and 30 min at 2 C.

To gain deeper insight into the electrochemical kinetics of S1, S2, and S3 electrodes, cyclic voltammetry (CV) was conducted over a voltage window of 1.7–2.8 V at scan rates ranging from 0.1 to 0.5 mV s^−1^. As shown in the contour plots of the CV curves (Figure 3a,b,c), all three electrodes exhibited three distinct redox peaks. A prominent oxidation peak (denoted as Peak A) appeared around 2.4 V and is attributed to the oxidation of Li_2_S/Li_2_S_2_ to S_8_. Two reduction peaks, observed at approximately 1.9 V (Peak B) and 2.3 V (Peak C), correspond to the stepwise reduction of S_8_ to higher‐order soluble polysulfides (LiPSs, e.g., Li_2_S_8_, Li_2_S_6_, and Li_2_S_4_), followed by their conversion into lower‐order, insoluble LiPSs (e.g., Li_2_S_2_ and Li_2_S), respectively.

Notably, the S3‐based cell displayed the highest current response across all redox events, accompanied by the smallest voltage gap between oxidation and reduction peaks, regardless of scan rate. Furthermore, the half‐peak widths S3‐based cell for Peaks A, B, and C were 0.13, 0.09, and 0.08 V, respectively, narrower than those observed for the S1‐based (0.35 V for peak A, 0.20 V for peak B, 0.16 V for peak C) and S2‐based (0.19 V for peak A, 0.15 V for peak B, 0.13 V for peak C) cells. These results suggest that the S3‐based battery has faster redox reaction kinetics of LiPSs conversion and lower polarization compared to S1 and S2‐based batteries.

To further evaluate the mass transport kinetics in batteries assembled with S1, S2, and S3 electrodes, the chemical diffusion coefficient of Li^+^ (*D*_Li+_) was calculated using the Randles‐Sevcik equation: [73]

(1) $$i_{p}=\left(2.69\times 10^{5}\right)n^{3/2}Av^{1/2}D_{Li}^{1/2}C_{Li}$$

In this equation, *i_p_* is the peak current, n is the number of electrons involved in the redox reaction (*n* = 2), A is the electrode area (*A* = 0.88 cm^2^), v is the scan rate (*v* = 0.1 mV s^−1^), and *C_Li_* is the Li^+^ concentration in the electrolyte (*C_Li_* = 1 × 10^−3^ mol mL^−1^). The *D_Li_* values were determined from the linear relationship between *i_p_* and *v* ^1/2^ for the three redox peaks, designated as peak A, peak B, and peak C, as shown in Figure S17. The calculated *D_Li_* values for the S1 electrode at peaks A, B, and C are 2.8 × 10^−8^ cm^2^ s^−1^, 1.2 × 10^−8^ cm^2^ s^−1^, and 8.4 × 10^−9^ cm^2^ s^−1^, respectively, and for the S2 electrode are the 1.9 × 10^−7^ cm^2^ s^−1^, 1.2 × 10^−7^ cm^2^ s^−1^, and 6.3 × 10^−8^ cm^2^ s^−1^ for peak A, B, and C. In comparison, the S3‐based battery exhibits the highest *D_Li_* values of 3.0 × 10^−7^ cm^2^ s^−1^, 1.9 × 10^−7^ cm^2^ s^−1^, and 9.3 × 10^−8^ cm^2^ s^−1^ at the same peaks, as indicated in Figure 3d. The enhanced ion diffusion kinetics observed in the S3 electrode can be attributed to its dual gradient architecture, which facilitates a more uniform Li^+^ concentration gradient throughout the entire electrode thickness, compared to the S1 and S2 where the Li^+^ is non‐uniformly distributed. The galvanostatic intermittent titration technique (GITT) results further provide a qualitative evaluation of the Li⁺ diffusion behavior during the repeated charge–discharge processes. As shown in Figure S18, the *D*_Li_ of all electrodes increases around 2.2 V during charging and 2.3 V during discharging, which corresponds to the formation of soluble LiPSs that enhance ionic transport. Nevertheless, the overall *D*_Li_ values of S3 remain consistently higher than those of S2 and S1, in good agreement with the Randles‐Sevcik analysis, further demonstrating that the dual‐gradient architecture effectively accelerates Li⁺ transport and facilitates LiPSs conversion kinetics. The Tafel slopes corresponding to the oxidation peak (peak A) and the two reduction peaks (peaks B and C) were calculated to quantitatively assess the influence of the dual‐gradient structures on the electrocatalytic behavior toward LiPSs conversion. As shown in Figure 3e and Figure S19, the S2‐based battery exhibits lower Tafel slopes of 28 142, and 43 mV dec^−1^ for peaks A, B, and C, respectively, compared to the corresponding values of 36, 198, and 71 mV dec^−1^ for the S1‐based battery. This result suggests that the three‐dimensional hollow architecture of the S2 electrode with lower tortuosity is more favorable for LiPSs conversion than the high‐tortuosity two‐dimensional planar structure of S1. Notably, the S3‐based battery demonstrates the lowest Tafel slopes of 19, 96, and 27 mV dec^−1^ at the respective redox peaks, highlighting that the dual‐gradient structured S3 electrode can further enhance the kinetics of both the oxidation and reduction reactions of LiPSs during cycling, surpassing even the performance of the S2 electrode. The relative enhancement in kinetics among S1, S2, and S3 electrodes primarily stems from the progressive optimization of their structural architectures. This synergy between catalyst material selection and structural design contributes to the superior redox kinetics observed in the S3 electrode.

To further elucidate the intrinsic electrochemical reactivity of the S1, S2, and S3 electrodes toward LiPS redox kinetics without the influence of lithium metal anodes and solid active materials, three symmetrical cells were assembled using identical Li_2_S‐free S1, S2, and S3 electrodes and a common electrolyte containing Li_2_S_6_. CV was performed within a voltage window of ‐1.5–1.5 V at a scan rate of 5 mV s^−1^. As shown in Figure 3f, all three cells display two well‐defined redox peaks, corresponding to the reversible conversion between soluble LiPSs and Li_2_S. Notably, the symmetrical cell with the S3 electrode exhibits higher peak currents and lower polarization than its S1 and S2 counterparts, suggesting improved electrochemical reversibility and enhanced LiPSs conversion kinetics in the S3 electrode.

The decomposition and nucleation of Li_2_S, involving solid‐to‐liquid and liquid‐to‐solid phase transitions, respectively, are widely recognized as the rate‐determining steps during the charge and discharge processes due to their high energy barriers [74]. To probe these critical steps, potentiostatic charge and discharge experiments were conducted to evaluate the decomposition behavior of Li_2_S during charging and the nucleation and growth of Li_2_S during discharging. As shown in Figure 3g, the Li_2_S precipitation capacities of the S1 and S2‐based batteries are 164.4 and 278.1 mAh g^−1^, respectively, with corresponding nucleation response times of ∼6910 s and ∼4820 s. In contrast, the S3‐based battery delivers the highest Li_2_S precipitation capacity of 382.6 mAh g^−1^ and the shortest nucleation response time of ∼3540 s. Furthermore, in the potentiostatic charge process, the S3‐based battery also exhibits the highest Li_2_S dissolution capacity of 552.7 mAh g^−1^ and the shortest dissolution response time of ∼4450 s, outperforming the S1‐ and S2‐based batteries, which show capacities of 339.6 and 477.3 mAh g^−1^ and response times of ∼6230 and ∼7460 s, respectively, as shown in Figure S20. These findings underscore that the structural design of the S3 electrode effectively facilitates both the nucleation and growth of Li_2_S during discharge and its dissolution during charge, thereby accelerating the overall conversion kinetics of the LiPSs redox process. These improvements in Li_2_S precipitation and dissolution kinetics are not only attributed to the spatially optimized structure that facilitates uniform Li^+^ distribution, but also to the catalytic effect of the Fe_2_O_3_/Fe‐N‐C components, which lower the energy barriers associated with solid‐phase transformations, as supported by both DFT calculations and prior studies.

The evolution of electrochemical resistance in the S1, S2, and S3 electrodes was evaluated using in situ electrochemical impedance spectroscopy during the second charge and discharge cycle, as shown in Figure 3 h and Figures S21 and S22. The Nyquist plots display two semicircles at high and medium frequencies, followed by a short, inclined tail at low frequencies. To quantitatively analyze these impedance responses, all spectra were fitted using an equivalent circuit model as shown in Figure S23. The specific resistance components, including electrolyte resistance (*R*_e_), interfacial contact resistance (*R*_s_), and charge transfer resistance (*R*_ct_), were also extracted and plotted against the corresponding charge states for S1, S2, and S3 (Figure S24) with total resistance values summarized at each state of charge and discharge in Figure 3h. The *R*_e_ remains around 5 Ω across all samples, indicating stable bulk ionic conduction. In contrast, the *R*_s_ and *R*_ct_ show clear differences. For Li–S batteries, the *R*_s_ includes contributions from the electronic resistance of the electrode and the resistance arising from the formation of a solid electrolyte interphase (SEI). The reduced *R*_s_ in S3 suggests that its dual‐gradient electrode design leads to less SEI accumulation and thus a more favorable Li^+^ transport kinetic for a reduced overpotential. More notably, the evolution of the second semicircle reflects substantial variations in *R*_ct_. This resistance peaks near 0% state of charge (SoC, point A) and 100% depth of discharge (DoD, point I), highlighting sluggish kinetics during the formation and decomposition of solid Li_2_S_2_/Li_2_S. At all charge states, S3 exhibits the lowest *R*_ct_, followed by S2 and then S1. Since high *R*_ct_ correlates with poor faradaic kinetics, these results confirm that the S3 electrode promotes the most efficient redox kinetics for sulfur conversion [75, 76]. The consistently reduced interfacial and charge transfer resistances in the S3 electrode underscore the combined benefit of minimized SEI formation due to dual‐gradient design and the electrocatalytic function of Fe_2_O_3_/Fe‐N‐C in accelerating LiPSs conversion. In addition, in the distribution of relaxation times (DRT) analysis, the S3 electrode exhibits the narrowest peak distribution and the smallest relaxation times, as shown in Figure S25, indicating the most homogeneous ion and electron transport. In contrast, S1 and S2 display broader and more intense peaks, reflecting sluggish charge‐transfer and diffusion kinetics. These DRT results are fully consistent with the Nyquist analysis, further confirming that the dual‐gradient structure of S3 effectively minimizes polarization and synchronizes redox reactions across the electrode thickness.

To elucidate the phase evolution of sulfur species during electrochemical cycling, in‐situ XRD measurements were conducted on S1, S2, and S3 electrodes via a custom‐designed cell, as schematically illustrated in Figure S26. At the beginning of the charging process of the S3 electrode, two distinct diffraction peaks are observed at 2θ angles of 26.9° and 31.3°, corresponding to the (111) and (200) crystal planes of Li_2_S, respectively. As charging progresses, the intensities of these peaks gradually diminish, indicating the continuous consumption of Li_2_S. Toward the end of charging, two new peaks emerge at 26.7° and 28.3°, which are attributed to the (311) and (008) planes of orthorhombic S_8_, suggesting the conversion of soluble LiPSs to solid S_8_ during the SER [77, 78]. In the subsequent discharge process, these crystalline S_8_ peaks progressively disappear, reflecting the reverse solid‐to‐liquid transition as S_8_ is reduced to form soluble LiPSs through the SRR. Finally, by the end of discharge, the original Li_2_S peaks at 26.9° and 31.3° reappear, indicating the successful regeneration of Li_2_S and completion of the redox cycle.

To quantitatively analyze the evolution of the solid phases, the relative intensities (RI, calculated based on Note S1) of key diffraction peaks of solid phases during the charge–discharge process are plotted in Figure 4b for all three electrodes. Notably, the rate of Li_2_S consumption in the S3 electrode closely matches that of the S2 electrode (Figure S27, In situ XRD of S2 electrode), where Li_2_S has almost completely disappeared at 36.4% SoC as indicated in Figure 4b. In contrast, the S1 electrode exhibits a significantly slower rate of Li_2_S consumption, with residual Li_2_S remaining unconverted at the end of the charging process, as shown in Figure S28 and Figure 4b. This observation suggests that the S2 and S3 electrodes exhibit comparable kinetics for Li_2_S activation, and both display more favorable solid‐state reaction kinetics compared to the S1 electrode.

As the SER progresses, the RI of S_8_ in the S3 electrode reaches 88% at approximately 91% SoC. Although S_8_ is detected earlier in the S2 electrode at 72% SoC with an RI of 34%, its intensity only reaches 51% at 91% SoC, remaining significantly lower than that of the S3 electrode. In the S1 electrode, the RI of S_8_ reaches just 21% at the same SoC, underscoring limited solid product formation. These results indicate that the S3 electrode achieves the highest yield of S_8_ at the end of charging, contributing to the enhanced charge capacity observed in the S3‐based cell. In addition, the S_8_ peak in the S3 electrode displays the narrowest full width at half maximum among all electrodes, which indicates higher crystallinity of the regenerated S_8_ in the S3 electrode, suggesting more favorable kinetics for S_8_ nucleation and crystal growth during the SER.

Upon discharge, the S3 electrode exhibits the fastest S_8_ consumption, with nearly 100% of the relative intensity of the orthorhombic S_8_ peak diminishing at ∼27% DoD, indicating the most favorable solid‐state conversion kinetics. In contrast, the consumption of S_8_ in the S2 electrode is noticeably slower than in the S3 electrode, with complete depletion of the S_8_ peak occurring at ∼46% DoD. The S1 electrode shows the slowest kinetics, with complete S_8_ disappearance delayed until approximately 64% DoD. The improved kinetics for S_8_ consumption in S2 over S1 likely result from reduced electrode tortuosity, which facilitates better Li^+^ transport and provides more active reaction sites.

At the end of discharge, even though the S2 electrode shows the earliest appearance of Li_2_S at ∼91% DoD, the Li_2_S formed in the S3 electrode exhibits the narrowest full width at half maximum and the highest RI among all electrodes, indicating both higher crystallinity and a larger quantity of regenerated Li_2_S.

Overall, the in situ XRD results suggest that 3D hollow structures promote more efficient solid‐liquid and liquid‐solid conversion kinetics compared to 2D planar structures. While the S2 electrode enables faster initial formation of S_8_ and Li_2_S, the S3 electrode achieves faster consumption of S_8_ during discharge. Importantly, the high RIs and sharp diffraction peaks of both S_8_ and Li_2_S in the S3 electrode indicate that the dual‐gradient design not only accelerates redox reaction rates but also improves the structural order of the solid products, ultimately contributing to enhanced reversibility and long‐term cycling performance.

To further understand the causation of the difference of SER and SRR processes among S1, S2, and S3 electrodes, the dynamic evolution of soluble LiPSs was monitored by in situ Raman spectroscopy, as shown in Figure 4c and Figures S29, and S30.

A consistent peak observed at 282 cm^−1^ is attributed to lithium bis(trifluoromethanesulfonyl)imide (LiTFSI) in the electrolyte [79]. At the initial charging process in all three electrodes, two peaks appear at 200 and 450 cm^−1^, which correspond to Li_2_S_4_ species, suggesting the conversion of the solid LiPSs to soluble LiPSs [3]. As charging progresses, a new peak emerges around 400 cm^−1^, corresponding to the formation of Li_2_S_6_, indicating the further reduction of Li_2_S_4_. As the SER continues, two Raman peaks emerge at approximately 150 and 473 cm^−1^ in all three electrodes, corresponding to the formation of Li_2_S_8_ [3]. During the discharge, the two peaks corresponding to Li_2_S_8_ appear at first due to the solid‐liquid conversion between S_8_ and Li_2_S_8_. Then followed by the soluble LiPSs conversion from long chain to medium chain, Li_2_S_6_ and Li_2_S_4_ have gradually been detected again. To quantitatively compare the evolution of soluble LiPSs, the peak of each species (i.e., ∼450 cm^−1^ for Li_2_S_4_, ∼400 cm^−1^ for Li_2_S_6_, and ∼152 cm^−1^ for Li_2_S_8_) was selected, and its intensity was normalized to the LiTFSI peak (Note S2). The temporal evolution of each soluble LiPSs species during the charge and discharge processes is plotted in Figure 4d.

During the SER, the RI of Li_2_S_4_ in the S3 electrode shows an increase to 0.53 at ∼16.7% SoC and then drops to 0.64 at ∼50% SoC, respectively. Subsequently, the RI of Li_2_S_6_ reaches 0.73 at approximately 33.4% SoC, decreases to 0.61 at 66.7% SoC, and nearly vanishes at 83.3% SoC with a residual RI of only 0.06, reflecting a rapid and complete conversion. For the Li_2_S_8_ signal, it is first detected at ∼50% SoC with an RI of 0.79 and drops to 0.09 at ∼100% SoC, indicating the full liquid to solid conversion from Li_2_S_8_ to S_8_.

In contrast, the RI of Li_2_S_4_ reaches 1.07 at approximately 16.7% SoC and decreases to 0.64 at around 50% SoC in the S2 electrode, suggesting a slightly faster initial formation of Li_2_S_4_ from Li_2_S_2_ but a slower consumption rate of Li_2_S_4_ than that observed in the S3 electrode. In addition, Li_2_S_6_ in the S2 electrode exhibits higher RIs of 1.08 and 1.52 at 33.4% and 66.7% SoC, respectively, and still retains a significant RI of 0.24 at full charge (100% SoC). This persistent signal indicates incomplete conversion of Li_2_S_6_, once again highlighting slower soluble LiPSs conversion kinetics in the S2 electrode compared to the S3 electrode. Li_2_S_8_ appears earlier at 33.4% SoC (RI = 0.18) but remains at 0.35 by 100% SoC in the S2 electrode, confirming poor conversion to S_8_. Moreover, the accumulation of soluble LiPSs in the S2 electrode appears to contribute to a decline in subsequent conversion kinetics. Specifically, during the initial stage of charging, in situ XRD confirms that the solid‐to‐liquid conversion kinetics of Li_2_S are comparable between the S2 and S3 electrodes, indicating similar Li_2_S activation behavior. However, as charging proceeds and soluble intermediates such as Li_2_S_6_ and Li_2_S_8_ accumulate within the S2 electrode, they may begin to block electrochemically active sites and hinder further redox reactions. This hypothesis is supported by the in situ XRD results discussed above, indicating that after charging, the rate of S_8_ consumption in the S2 electrode during discharge becomes noticeably slower than in the S3 electrode. Therefore, the presence of accumulated LiPSs not only reflects incomplete intermediate conversion but also introduces a feedback limitation that deteriorates the kinetics of subsequent solid‐liquid conversion reactions. However, the kinetics of S2 in S_8_ consumption are still faster than those in the S1 electrode, likely resulting from reduced electrode tortuosity, which facilitates Li^+^ transport and enhances the accessibility of active reaction sites, thereby accelerating the overall reaction process.

The S1 electrode exhibits the slowest reaction kinetics among the three designs. Owing to its high structural tortuosity, the RI of Li_2_S_4_ remains at 0.53 even at the end of charging. Additionally, the RIs of both Li_2_S_6_ and Li_2_S_8_ continue to increase throughout the charge process, implying delayed formation and incomplete consumption of intermediates. This behavior reflects significant kinetic limitations for both solid‐to‐liquid and soluble LiPSs conversions in the S1 electrode.

Upon discharge, the RI of Li_2_S_8_ in the S3 electrode reaches a peak value of 1.05 at approximately 16.7% DoD and rapidly declines to 0.07 by 50% DoD, indicating efficient consumption and favorable kinetics for both solid‐to‐liquid and soluble LiPSs conversion reactions. Followed by the formation of Li_2_S_6_, the first signal appears at ∼16.7% DoD and nearly disappears by ∼66.7% DoD with an RI of only 0.14, suggesting the efficient conversion of soluble LiPSs. Toward the end of the discharge process, Li_2_S_4_ forms as a downstream product from Li_2_S_6_ conversion, emerging at ∼33.4% DoD and being fully consumed by 100% DoD, highlighting complete conversion of soluble intermediates to solid‐state products.

In contrast, Li_2_S_8_ in the S2 electrode persists until ∼83.3% DoD, lasting significantly longer than in the S3 electrode. The prolonged presence of Li_2_S_8_ and delayed Li_2_S_6_ consumption in the S2 electrode likely led to residual LiPSs remaining at the end of discharge. Specifically, at 16.7% DoD, the RI of Li_2_S_6_ in the S2 electrode is 0.63, which is substantially higher than the 0.35 observed in the S3 electrode. Furthermore, at ∼66.7% DoD, the RI of Li_2_S_6_ remains as high as 0.83, nearly 6 times larger than that of the S3 counterpart, indicating pronounced kinetic retardation in soluble LiPSs conversion in the S2 electrode. Additionally, Li_2_S_4_ in the S2 electrode is not fully consumed by the end of discharge, ultimately limiting the quantity of Li_2_S formed and eventually reducing the achievable discharge capacity. However, the S2 electrode still outperforms the S1 electrode, in which both Li_2_S_6_ and Li_2_S_4_ are not fully consumed at the end of the discharge (Figure 4d and Figure S24).

Taken together with the results from the charging process, these findings indicate that although the S2 and S3 electrodes exhibit comparable kinetics for initial Li_2_S activation, the S3 electrode demonstrates markedly improved kinetics for the conversion of soluble LiPSs intermediates. This enhanced intermediate conversion underpins the superior performance of the S3 electrode in both the SER and SRR.

To better understand the origin of the different kinetics of soluble LiPSs conversion in the S2 and S3 electrodes, and hence the role of dual‐gradient structure as well as the 3D uniform channels structure, an FEA was carried out to visualize the temporal‐spatial evolution of the concentration of soluble LiPSs species (Li_2_S_4_, Li_2_S_6_, and Li_2_S_8_) during the charging process, as indicated in Figure 4e. The electrode thickness in the model was set to 500 µm, where one end, positioned at 0 µm, corresponds to the side contacted with the SP, and the other end, positioned at 500 µm, represents the side in contact with the CC.

In both S3 and S2, the concentration of each LiPS varies between SP and CC, where the concentration at the SP region reaches the expected value faster, leading to the concentration polarization along the electrode thickness direction. Specifically, in the S3 electrode, the Li_2_S_4_ concentration (C_Li2S4_) at the SP side reaches 50% at approximately 4.6% SoC, which is 7.4% earlier than the CC side, where the same concentration is reached at 12.0% SoC. As charging proceeds, C_Li2S4_ declines to 50% at 17.1% SoC on the SP side and at 32.8% SoC on the CC side, resulting in a SoC gap of 15.7% between the top and bottom of the electrode. In contrast, the S2 electrode exhibits a more pronounced disparity. The C_Li2S4_ at the SP side reaches 50% at around 3.9% SoC, while the CC side only achieves this level at 16.2% SoC. As charging progresses (Li_2_S_4_ to Li_2_S_6_), C_Li2S4_ falls to 50% at 14.3% SoC on the SP side but not until 38.4% SoC on the CC side, yielding a larger difference of 24.1%. As a result, the S3 electrode requires less time to reach complete depletion of Li_2_S_4_ in the entire electrode, leading to a shorter overall duration of Li_2_S_4_ presence. In the S3 electrode, the concentration of Li_2_S_6_ (C_Li2S6_) reaches 50% at the SP side of the electrode at 30.7% SoC and subsequently reaches the same C_Li2S6_ at the CC side at 42.6% SoC, resulting in a SoC difference of 11.9% between the two regions. In comparison, the S2 electrode exhibits a difference of 21.2% SoC between the SP and CC side, showing a larger polarization in the S2 electrode. Similarly, the difference between the SP side and the CC side of the S3 electrode, when the Li_2_S_8_ concentration (C_Li2S8_) reaches 50%, is 9% SoC, which is notably smaller than the difference of 24.6% SoC of the S2 electrode. Furthermore, by the end of the charging process (100% SoC), the C_Li2S8_ in the S2 electrode remains above 50% across the 320–500 µm depth range, indicating the accumulation of Li_2_S_8_ in the CC side.

The earlier appearance of LiPSs at the SP side of the S2 electrode, compared to the S3 electrode, can be attributed to the faster local reaction rate at the SP side of the S2 electrode, as previously discussed in Figure 1. This also explains why solid‐phase products such as S_8_ and Li_2_S are first detected in the S2 electrode, as these species preferentially form near the SP side during the SER and SRR. However, due to the larger concentration polarization across the S2 electrode, LiPSs generated at the CC side cannot be consumed promptly. This leads to their accumulation, which blocks active sites and suppresses subsequent conversion reactions, ultimately hindering both sulfur utilization and redox reversibility. Moreover, the faster reaction rate at the SP side of the S2 electrode contributes to a lower overpotential during the initial stage of charging, as evidenced by the lower voltage of 2.28 V at 20% SoC, compared to 2.37 V for the S3 electrode at the same SoC. However, as charging proceeds, the growing concentration polarization within the S2 electrode becomes increasingly pronounced, leading to a rapid rise in overpotential. As a result, beyond 50% SoC, the S2 electrode exhibits a higher overpotential than the S3 electrode, reflecting deteriorated reaction kinetics and increased transport limitations.

To investigate the effects of concentration polarization on sulfur redox reactions, the CUR of the S2 and S3 electrodes has been visualized as shown in Figure 4f. The S3 electrode demonstrates a more uniform CUR after 10, 20, and 30 min of charging at 2 C, achieving overall utilization values of 28%, 64%, and 93%, respectively. In contrast, the S2 electrode, due to the lower synchronicity of the reaction along the electrode depth, shows a disparity in utilization: the SP side is highly utilized, whereas the CC side remains underutilized. This non‐uniform reaction distribution results in lower overall utilization ratios of 31%, 61%, and 84% at the same time intervals, ultimately contributing to reduced discharge capacity. The dual‐gradient architecture of S3 not only ensures complete LiPSs conversion across the electrode depth but also synchronizes redox pathways, overcoming the spatially asynchronous behavior observed in the S2 electrode.

Beyond capacity, mechanical stability during discharge is also critical, as the conversion of Li_2_S to S_8_ can cause up to 78% volume expansion. This expansion generates significant mechanical stress within the electrode due to the mutual compression of active materials.

To evaluate the mechanical stress due to the volume changes during the discharge process, von Mises stress distributions, which indicate a risk of mechanical failure by reflecting the intensity of internal mechanical stress, were analyzed for both electrodes at 10, 20, and 30 min of charging and are shown in Figure 4f. The S3 electrode shows a homogeneous stress field with a lower gradient across the electrode, attributed to a more uniform volume variation with its evenly distributed CUR. Moreover, a wide range of pore sizes (i.e., hierarchical) ranging from the nanoscale to micron scale (as evidenced in Figure 2e3,f) provides effective buffering space to accommodate the volume change associated with Li_2_S formation and consumption. This uniform stress distribution in the S3 electrode prevents localized stress buildup during cycling and helps preserve electrode integrity.

In contrast, although the S2 electrode also features a similar porosity, the non‐uniform utilization results in an uneven distribution of mechanical stress. Greater stress accumulates in the top region, where reaction activity and Li_2_S consumption/deposition are more concentrated, while the bottom region experiences less stress accumulation due to the underutilized active materials within the S2 electrode. The pronounced stress gradient in the S2 electrode promotes structural damage, including pulverization of the active material.

Overall, this integrated experimental and computational study reveals that the S3 electrode achieves both superior ion transport and enhanced redox kinetics for SER and SRR, while simultaneously alleviating mechanical stress accumulation associated with large‐volume variation during phase transitions. The overall high performance of the S3 electrode arises from the deliberate dual‐gradient structural engineering, which plays the decisive role in enabling synchronized redox kinetics, mitigating stress accumulation, and ensuring long‐term stability, as well as from the catalytic activity of Fe_2_O_3_/Fe‐N‐C.

**FIGURE 5:**
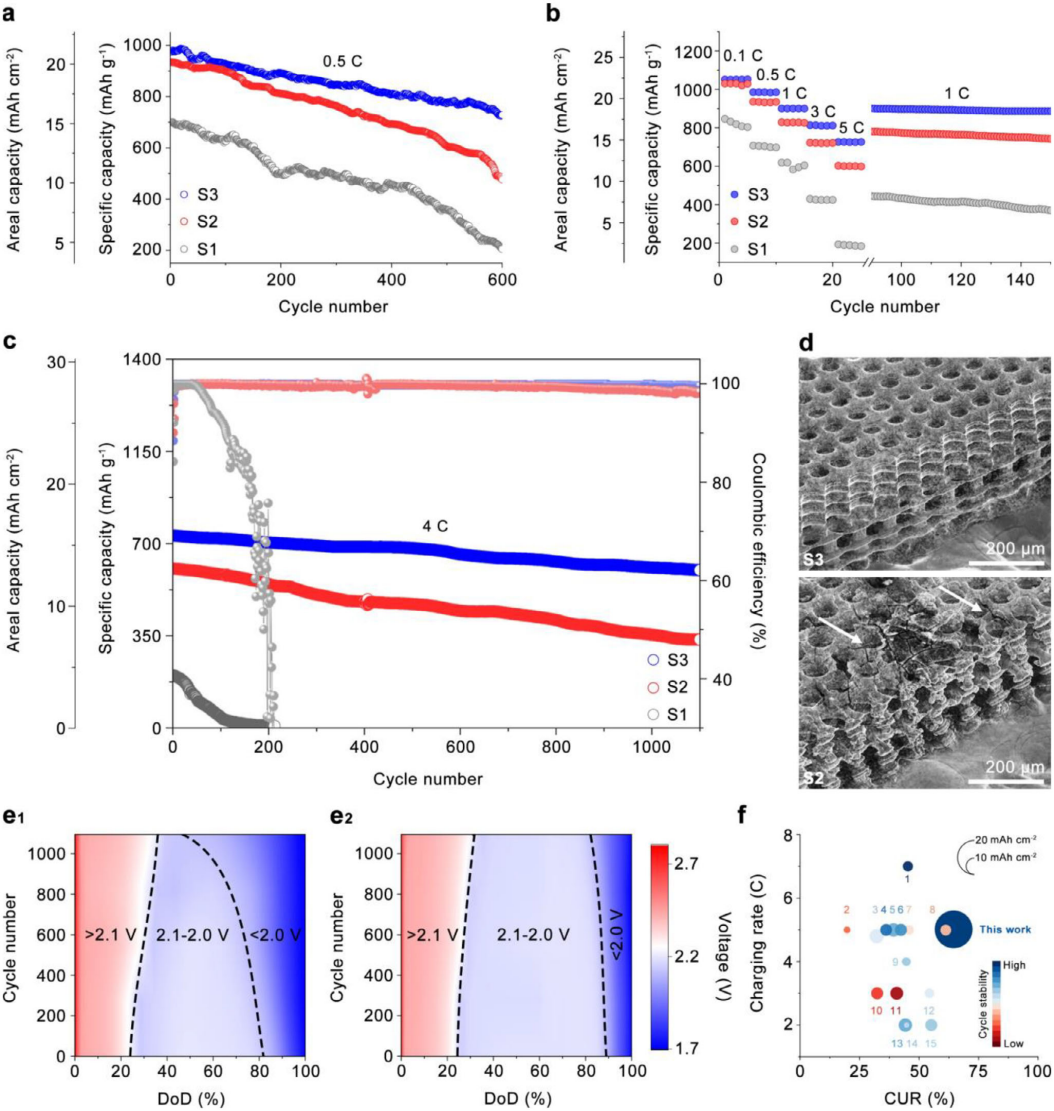
(a) Long‐term cycling stability of different cathodes at 0.5 C for 600 cycles. (b) Rate capability of the cathodes under current densities ranging from 0.1 to 5 C. (c) Extended cycling stability of the cathodes at 4.0 C for 1100 cycles. (d) SEM images of the S3 (top) and S2 (bottom, the cracks are pointed by white arrows) electrodes after long‐term cycling. (e) Contour plots of discharge profiles during 1100 cycles based on (e1) S2 electrode and (e2) S3 electrode. (f) Comparison of the cathode performance in this work with recently reported Li–S cathodes in terms of charging rate, CUR, areal capacity, and cycling stability.

The electrochemical performance of the S3 electrode was systematically evaluated using assembled coin cells. To highlight the structural advantages of the S3 architecture in Li–S batteries, a comparative analysis was conducted against S1 and S2 electrodes, as shown in Figure 5. At low current densities of 0.1 and 0.5 C, the S3‐based cell delivered the highest initial specific capacities of 1050 and 980 mAh g^−1^, respectively, corresponding to sulfur utilization ratios of 88.2% and 84.0% (Figure S31 and Figure 5a). In contrast, the S2‐based cell exhibited initial specific capacities of 1024 and 934 mAh g^−1^ at 0.1 and 0.5 C, while the S1‐based cell only reached 833 and 700 mAh g^−1^ under the same conditions. The improved capacity performance of the S3 cell also translates into an areal capacity of 21.2 mAh cm^−2^ under 0.5 C, exceeding the 4 mAh cm^−2^ benchmark typical of commercial lithium‐ion batteries (LIBs).

More importantly, the S3 electrode demonstrated excellent long‐term stability. At 0.1 C, the cell retained 85.6% of its initial capacity after 200 cycles (Figure S31), and at 0.5 C, it maintained 74% after 600 cycles (Figure 5a). These results represent the lowest capacity fading among the three electrode designs. In comparison, the S2‐based cell experienced capacity losses of 16% at 0.1 C over 200 cycles (Figure S32) and 37% at 0.5 C over 600 cycles (Figure 5a). The S1‐based cell showed even more pronounced fading, with 38% and 71% capacity losses under the same respective conditions. All three electrodes exhibit high CEs after the initial cycles, with the S3 electrode maintaining 99.89% over 600 cycles and demonstrating the best long‐term stability, followed by S2 (99.68%), while S1 shows greater fluctuations and lower efficiency, confirming that the structural design of S3 effectively enhances electrochemical reversibility under practical cycling conditions (Figure S33).

The rate performance of the batteries with these three electrodes was evaluated under various current densities ranging from 0.1 to 5 C, as compared in Figure 5b. Among them, the S3 electrode‐based battery demonstrates the best rate capability, delivering capacities of 1047, 984, 900, and 815 mAh g^−1^ at 0.1, 0.5, 1, and 3 C, respectively. Even at a high current rate of 5 C, it maintains a substantial capacity of 726 mAh g^−1^, corresponding to 15.7 mAh cm^−2^.

In comparison, the S3 electrode consistently outperforms both S1 and S2 electrodes across all current rates. Furthermore, when the current density returns to 1 C after high‐rate cycling, the capacity of the S3 electrode recovers to 900 mAh g^−1^, indicating superior reversibility and structural resilience. In contrast, the S1 and S2 electrodes show significant capacity irreversibility with markedly lower values compared to their original capacities at 1 C, suggesting much poorer high‐rate durability.

The outstanding rate performance of the S3 electrode‐based battery reflects highly efficient reaction kinetics, which can be attributed to its synergistic structural and compositional design. On the one hand, the well‐balanced distribution of Li^+^ concentration and the even distribution of soluble polysulfide intermediates along the electrode depth direction promote fast redox kinetics by reducing concentration polarization. On the other hand, the high catalytic activity of Fe_2_O_3_/Fe‐N‐C components embedded in the S3 structure further accelerates the reaction pathways, as supported by theoretical calculations (Figure 2) and experimental results (Figure S11). While the intrinsic catalytic activity benefits all electrodes containing these materials, the relative performance advantage of S3 is primarily due to its optimized structure, which enables more effective utilization of active sites and facilitates uniform redox reactions throughout the entire electrode.

The long‐term cycling performance of the S3 electrode was further evaluated at a high current density of 4 C. As shown in Figure 5c, the S3 electrode‐based battery delivered an initial capacity of 730 mAh g^−1^ (15.8 mAh cm^−2^) and maintained an average coulombic efficiency of 99.67% over 1100 cycles. Notably, it exhibited an extremely low‐capacity decay rate of only 0.016% per cycle. Even after prolonged cycling, the S3‐based battery retained a specific capacity of 600 mAh g^−1^ (13 mAh cm^−2^) well above the 4 mAh cm^−2^ benchmark of commercial LIBs, highlighting its promise for high‐loading applications. In addition, no distinguishable LiPSs‐related peaks were observed in the Raman spectra (Figure S34) of the S3 electrode after cycling, indicating effective suppression of polysulfide accumulation and excellent reversibility of sulfur redox reactions.

For comparison, the S2 electrode‐based battery delivered an initial capacity of 604 mAh g^−1^ under the same current density, with an average coulombic efficiency of 98.73% during 1100 cycles. Its capacity decay rate was 0.04% per cycle, which was approximately three times higher than that of the S3‐based battery and lower than that of the S1‐based cell. The higher CE of the S3 electrode can be attributed to its correlated dual‐gradient architecture, which synchronizes the generation and conversion of polysulfides along the electrode thickness. This structural regulation prevents local LiPSs accumulation, suppresses shuttle‐induced side reactions, and ensures more reversible redox reactions, thereby maintaining a high CE throughout prolonged cycling. However, Raman spectra (Figure S33) collected from the S2 electrode after cycling revealed persistent peaks at ∼200 and ∼450 cm^−1^, corresponding to residual Li_2_S_4_ species. These signals suggest incomplete conversion of LiPSs during the charge–discharge cycles, which may be particularly attributed to the bottom of the electrode. One plausible explanation is that the longer chain length of Li_2_S_8_ leads to slower migration from the low‐activity bottom region to the higher‐activity top region of the electrode, causing Li_2_S_8_ to accumulate and persist in poorly reactive zones.

The S1 electrode‐based battery showed the poorest performance among the three. It delivered an initial capacity of only 202 mAh g^−1^ at 4 C and failed to retain any meaningful capacity after just 200 cycles. Raman analysis of the S1 electrode after 200 cycles showed intense peaks corresponding to Li_2_S_4_, Li_2_S_6_, and Li_2_S_8_, respectively, as shown in Figure S33. This indicates severe accumulation of soluble LiPSs due to sluggish redox kinetics and pronounced concentration gradients across the electrode thickness. The highly tortuous, non‐continuous porous structure of the S1 electrode, which lacks sufficient through‐plane connectivity, hinders uniform reaction distribution. This leads to poor ionic accessibility and accumulation of inactive species, ultimately causing nanopore blockage and passivation of active materials.

SEM observations further reveal distinct morphological differences among the three electrodes after cycles. As shown in Figure 5d, the S3 electrode retained its highly ordered architecture and structural integrity even after prolonged cycling, benefiting from the correlated dual‐gradient design that homogenizes reaction distribution and alleviates local stress accumulation. By contrast, the S2 electrode displayed noticeable cracks near the separator side (as marked by arrows), which is a consequence of non‐uniform utilization and intensified concentration polarization, leading to the pulverization of the electrode. These results confirm that only the dual‐gradient architecture in S3 can effectively suppress electrode degradation, ensuring both mechanical stability and sustained electrochemical activity.

Moreover, EIS results after cycling further corroborate the kinetic differences among the three electrodes, as shown in Figure S34. The S1 electrode exhibited a high Rct value of 49.3 Ω, confirming its sluggish redox kinetics and poor reversibility compared to the S2 and S3 electrodes. In contrast, the S3 electrode maintained the lowest impedance value of 15.2 Ω after 1100 cycles, reflecting its superior charge transfer kinetics and stable interface, which are essential for maintaining high reversibility and reaction efficiency in Li–S systems.

To visualize the evolution of polarization behavior during high‐rate cycling, discharge potential maps at 4 C were analyzed for all electrodes, as shown in Figure 5e and S35. In these maps, the discharge process is divided by dashed lines into three distinct regions: >2.1 V (initiation region), 2.1–2.0 V (discharge plateau region), and <2.0 V (polarized region). In these maps, the discharge process is divided by dashed lines into three distinct regions: >2.1 V (initiation region), 2.1–2.0 V (discharge plateau region), and <2.0 V (highly polarized region). The S1 electrode‐based battery (Figure S35) exhibited a large, polarized region even in the early stage of cycling, primarily due to its high structural tortuosity and limited ion transport pathways. The S2 electrode showed somewhat reduced polarization, compared to S1, but still exhibited noticeable voltage depression from 800 to 1100 cycles (Figure 5e1). In sharp contrast, the S3 electrode‐based battery demonstrated a significantly narrower polarized region (Figure 5e2), indicating suppressed polarization throughout prolonged cycling. This improvement is attributed to the dual‐gradient structural design of the S3 electrode, which enables a balanced spatial distribution of Li ions and soluble LiPSs along the electrode thickness. Such uniform distribution promotes synchronized redox reactions, facilitates high utilization of the active material, and maintains fast reaction kinetics even at high C‐rates. However, the imbalance in Li^+^ and LiPSs concentration in the S2 electrode, along with asynchronous conversion between the top and bottom regions, likely leads to underutilization of active sites, especially near the bottom of the electrode. Over extended cycles, this polarization imbalance is further intensified by the continuous accumulation of unconverted LiPSs in low‐activity regions. This accumulation not only increases the ionic concentration difference but also limits the effective reaction depth, further slowing the redox kinetics and amplifying capacity fading. Moreover, non‐uniform tensile and compressive stress distributions induced by uneven sulfur utilization contribute to the mechanical degradation of the electrode. The development of cracks and delamination results in the physical loss of active material, which reduces the effective surface area available for electrochemical reactions and increases the local current density, leading to further polarization and rapid performance decay. In the case of the S1 electrode with its 2D planar structure, the polarization is already pronounced within the first 100 cycles. This behavior highlights the inherently sluggish kinetics of S1, stemming from its high tortuosity and poor through‐plane ion and electron transport. These limitations ultimately result in rapid electrode passivation, capacity loss, and inferior long‐term performance.

To quantitatively evaluate the electrochemical performance of the S3 electrode, a comparative analysis was conducted against previously reported studies from the past three years. Key performance metrics, including areal capacity, rate capability, cycle stability, and sulfur utilization, were considered. Specifically, the CUR was calculated as the ratio of the measured discharge capacity to the theoretical capacity of Li_2_S multiplied by the experimentally determined sulfur mass fraction (30.8 wt%, obtained from TGA, Figure S16). As shown in Figure 5f and detailed in Table S2, the electrochemical performance achieved in this work ranks among the highest reported for both Li_2_S‐based cathodes and 3D printed Li–S electrodes. Specifically, the S3 electrode demonstrates competitive advantages in combining high areal capacity, excellent rate performance, long cycle life, and efficient sulfur utilization, confirming the effectiveness of the dual‐gradient structural design.

To further highlight the advantages of the correlated dual‐gradient structural design in Li–S battery electrodes, electrochemical performance was evaluated under challenging conditions to assess both reaction kinetics and practical feasibility. Specifically, the batteries were tested at −10°C to simulate low temperature environments. As shown in Figure S36, the S3 electrode‐based battery delivered an initial capacity of 639 mAh g^−1^ at 1 C, with a capacity retention of 96.1% after 70 cycles. This performance significantly exceeds that of the S2 and S1 electrode‐based batteries, which achieved capacities of 590 and 389 mAh g^−1^ with retention rates of 93.2% and 86.8%, respectively. Moreover, the S3 electrode‐based battery delivered an average CE of 99.91% after 70 cycles, surpassing that of the S2 (92.22%) and S1 (86.95%) counterparts (Figure S37). These results indicate that the superior low‐temperature performance of the S3 electrode is attributed to the synergistic effects of both the Fe_2_O_3_/Fe‐N‐C catalytic component and the rationally engineered dual‐gradient structure, which collectively promote more efficient Li^+^ transport and polysulfide conversion kinetics even under harsh conditions. In contrast, the high tortuosity of the S1 electrode intensifies Li^+^ transport barriers and slows LiPSs redox at low temperatures. The 3D uniform channels of the S2 electrode improve ion transport but fail to balance Li^+^ concentration across the electrode, leading to localized LiPSs accumulation and prolonged solid‐liquid conversion, which exacerbate polarization and limit overall reaction kinetics.

Building upon this demonstration of kinetic superiority, the next step was to evaluate the practical compatibility of the S3 electrode in full‐cell configurations. Benefiting from the lithium‐containing nature of Li_2_S as the active material in all S1, S2, and S3 cathodes, the use of a lithium metal anode can be avoided, enabling the construction of lithium‐free full cells. To evaluate practical applicability and safety, a commercial silicon‐carbon (Si/C) anode was employed to replace the lithium metal counterpart. This configuration eliminates the risk of lithium dendrite formation and mitigates undesirable side reactions, thus enhancing battery safety.

**FIGURE 6:**
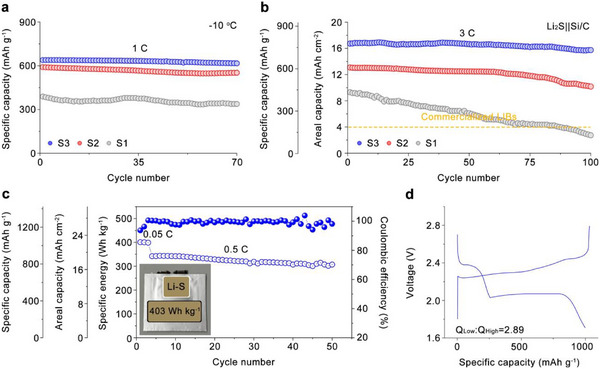
(a) Cycling performance of the cathodes at −10 °C under 1 C. (b) Cycling performance at 3 C using Si/C anodes in coin cells. (c) Cycling performance of single‐layer pouch cells with Li metal anodes under 0.05 C (first 3 cycles) and 0.5 C. (d) Voltage‐capacity profile of the second cycle corresponding to the pouch cell shown in (c).

As shown in Figure 6b, the full cell assembled with the S3 electrode delivers an initial specific capacity of 774 mAh g^−1^ at a current density of 3 C, corresponding to an areal capacity of 16.7 mAh cm^−2^. In comparison, the full cell using the S2 electrode achieves an initial capacity of 605 mAh g^−1^ (13.1 mAh cm^−2^), while the S1‐based full cell only reaches 431 mAh g^−1^, approximately half of that obtained with the S3 configuration. After 100 cycles, the S3‐based full cell maintains a capacity retention of 94.1%, outperforming the S2 and S1 counterparts, which show retention rates of 77.8% and 29.4%, respectively. Moreover, the S3‐based full cell also exhibits reduced polarization and more efficient sulfur utilization compared to S1‐ and S2‐based full cells, consistent with the voltage‐capacity profiles shown in Figure S38. Over extended cycling, the S3‐based full cell maintains highly stable Coulombic efficiency with minimal fluctuations, while the S1‐ and S2‐based cells show noticeable instabilities (Figure S39). These results underscore that the rational dual‐gradient structural design of the S3 electrode not only enhances sulfur utilization but also contributes to improved structural stability and reversibility in full‐cell configurations, supporting the development of safer, high‐performance, lithium‐free Li–S batteries.

To further validate the practical potential of the S3 electrode beyond coin and full‐cell configurations, an ampere‐hour‐level pouch cell was assembled under limited electrolyte conditions and realistic mechanical constraints. The pouch cell was constructed using an S3 electrode with dimensions of 2.2 by 2.2 cm^2^, laminated onto an aluminum current collector. A lithium‐coated copper foil served as the anode, and the electrolyte content was precisely controlled to achieve an electrolyte‐to‐sulfur (E/S) ratio of 7.2. Before performance testing, the pouch cell underwent three formation cycles at 0.05 C to stabilize the interface, as shown in Figure 6c. The voltage‐capacity profile of the second cycle, shown in Figure 6d, displays a discharge capacity of 22.1 mAh cm^−2^, corresponding to a specific energy of 403 Wh kg^−1^. The detailed calculation parameters are summarized in Table S4. Notably, the Q_L_/Q_H_ ratio is 2.89, closely matching the value observed in the coin cell configuration, which indicates that the electrochemical advantages of the S3 electrode are effectively retained in the pouch cell setup. The pouch cell operated at 0.05 and 0.5 C with a 90.1% of the capacity retention after 50 cycles and an average coulombic efficiency of 99.28%. These results confirm that the S3 electrode, with its tailored structural design, not only delivers strong performance under practical constraints but also holds promise for large‐scale Li–S battery applications.

## 3 Conclusion

In summary, the S3 electrode, featuring a correlated dual‐gradient structure, was fabricated from a sulfur‐ and iron‐containing photo‐initiable monomer solvent through a unique combination of a programmable high‐resolution SLA manufacturing and subsequent pyrolysis‐induced carbonization. In the resulting Li_2_S@Fe_2_O_3_/Fe‐N‐C electrode, Li_2_S is uniformly embedded within a nitrogen‐rich carbon matrix decorated with octahedral Fe_2_O_3_ nanoparticles. This architecture provides three critical advantages: (1) the dual‐gradient structure mitigates concentration polarization, thereby synchronizing CUR across the electrode depth and enhancing electro‐chemo‐mechanical stability; (2) the synergistic catalytic activity of Fe_2_O_3_/Fe‐N‐C reduces the electrochemical polarization by lowering the reaction energy barriers, thereby accelerating kinetics for both SER and SRR; and (3) the carbonized conductive framework minimizes the ohmic polarization by providing continuous electronic pathway. As a result, the S3 electrode delivers 15.7 mAh cm^−2^ (726 mAh g^−1^) at 5 C with a mass loading of 21.6 mg cm^−2^ and exhibits remarkable long‐term cycling stability over 1100 cycles at 4 C with a low‐capacity decay of only 0.016% per cycle.

Further in situ characterizations combined with computational simulations reveal that the S3 electrode effectively prevents the accumulation of soluble LiPSs during the SER and SRR by mitigating concentration polarization, thereby ensuring spatially synchronized conversion throughout the redox process. At the initial solid‐liquid conversion stage, both S2 and S3 electrodes exhibit comparable reaction rates, indicating similar levels of electrochemical polarization. However, in subsequent stages, the larger concentration polarization in the S2 electrode leads to asynchronous LiPSs conversion and results in LiPSs accumulation. These accumulated intermediates gradually cover the catalytic active sites, reducing their availability and slowing down the subsequent liquid‐solid conversion. In contrast, the S3 electrode achieves a more complete and continuous conversion of soluble LiPSs, producing larger quantities of final products (S_8_/Li_2_S) and thereby yielding higher CUR. These findings underscore that rational electrode structural design to promote spatially synchronized redox reactions not only enhances overall electrode utilization but also provides sustained improvements in reaction kinetics. Moreover, the spatially synchronized reaction status throughout the S3 electrode depth promotes a uniform electrode deformation, preventing localized stress accumulation and thus avoiding electro‐chemo‐mechanical failure. This finding highlights that the spatial synchronization of reactions along the electrode depth also governs its electro‐chemo‐mechanical stability, since loss of synchronization can induce mechanical failure such as cracking and pulverization, which is otherwise observed at the SP side of the S2 electrode.

This work demonstrates the importance of integrating both material engineering and structural design to achieve high areal capacities, excellent rate capability, and superior cycling stability in high‐mass‐loading Li–S cathodes. The preparation strategy and mechanistic insights presented here provide a blueprint for the development of fast‐charging and high‐power‐density electrodes for next‐generation energy storage systems.

## Conflicts of Interest

The authors declare no conflicts of interest.

## Supporting information




**Supporting File**: adma71916‐sup‐0001‐SuppMat.docx

## Data Availability

The data that support the findings of this study are available from the corresponding author upon reasonable request.
