# Supplementary material for: Correlated Dual‐Gradient Electrodes Enabling Spatially Synchronized Sulfur Redox in High‐Mass‐Loading Li–S Batteries Under High Current Densities
Source: Adv Mater. 2025 Dec 26;38(10):e17190. doi: 10.1002/adma.202517190 (PMC12910539; doi:10.1002/adma.202517190)
Supplement: Supplementary file 1 — Supporting File: adma71916‐sup‐0001‐SuppMat.docx [file ADMA-38-e17190-s001.docx]

**Supporting Information**

**Title: Correlated dual-gradient electrodes enabling spatially synchronized sulfur redox in high-mass-loading Li-S batteries under high current densities**

Author: Yuxuan Zhang, Yeongjun Oh, Jinwook Baek, Minyoung Kim, Zachary Didat, Han Wook Song, and Sunghwan Lee*

Y. Zhang, Y. Oh, J. Baek, M. Kim, Z. Didat, S. Lee

School of Engineering Technology

Purdue University

West Lafayette, IN 47907, United States

*Email: [sunghlee@purdue.edu](mailto:sunghlee@purdue.edu)

H. W. Song

Convergence Research Center for Meta-Touch

Korea Research Institute of Standards and Science (KRISS)

Daejeon, 34413, Republic of Korea

**Table of Contents**

Experimental Section

Supplementary Notes

Supplementary Figures and Tables

Supplementary References

**Experimental Section**

**Fabrication of Electrodes**

The fabrication process consisted of three stages: (a) preparation of the printable precursor mixture, (b) stereolithography (SLA)-based 3D printing, and (c) pyrolysis followed by oxidative chemical vapor deposition (oCVD) of a PEDOT coating.

**a. Precursor Preparation:**

Lithium sulfate (Li_2_SO_4_, 99.5%, Sigma-Aldrich) and iron (III) nitrate nonahydrate (Fe(NO_3_)_3_·9H_2_O, 98%, Thermo Scientific Chemicals) were mixed at a weight ratio of 4:1 by adding 4.0 g of Li_2_SO_4_ and 1.0 g of Fe(NO_3_)_3_·9H_2_O into a glass vial. Subsequently, 12 g of triallyl-1,3,5-triazine-2,4,6-(1H,3H,5H)-trione (TTT, 98%, Sigma-Aldrich) was added to the mixture, followed by stirring at 600 rpm on a magnetic hotplate at room temperature for 3 hours. After homogenization, 0.12 g of 2,2-dimethoxy-2-phenylacetophenone (DMPA, 99%, Sigma-Aldrich) was added as the photoinitiator. To prevent premature polymerization of TTT monomer due to light sensitivity, the vial was immediately wrapped in aluminum foil and stirred for 1 hour. The resulting mixture was then stored in the dark until printing.

**b. 3D Printing of Multiscale Substrates:**

A procured LCD-based printer (Phrozen Sonic Mini 8K, Phrozen3D Technology Ltd.) was used to fabricate the microstructured porous substrates. The 3D model was designed using CAD software and sliced using CHITUBOX, with optimized parameters for layer height, exposure duration, lifting distance, and lifting speed to ensure uniform resin flow and accurate feature resolution. The resin was selectively photopolymerized using a 405 nm LED light source. After printing, the green body electrodes were rinsed thoroughly with isopropanol (99.5%) to remove unpolymerized resin and dried under ambient conditions in a fume hood for 30 minutes.

**c. Pyrolysis and oCVD Coating:**

The printed structures were pyrolyzed in a tube furnace under a nitrogen atmosphere. The pyrolysis protocol involved a two-step heating process: first, the temperature was ramped to 450 ^o^C at a rate of 1 ^o^C min^-1^ and held at 450 ^o^C for 2 hours; then, it was further increased to 950 ^o^C at the same ramp rate and maintained for 4 hours before naturally cooling to room temperature. The pyrolyzed cathodes were subsequently coated with a conformal layer of oxidatively polymerized PEDOT via oCVD, following our previously reported procedures.^[1]^

**Electrochemical measurements**

Li-S coin cells were assembled using lithium metal foil as the anode, a Celgard polypropylene (PP) membrane as the separator, and 150 μL of electrolyte in CR2032-type coin cells. The electrolyte consisted of 1 M lithium bis(trifluoromethanesulfonyl)imide (LiTFSI) in a 1:1 volume ratio mixture of 1,3-dioxolane (DOL) and 1,2-dimethoxyethane (DME), with the addition of 0.2 M lithium nitrate (LiNO_3_) as an additive. The Si/C anode exhibits a mass loading of 12-13 mg cm^-2^ with an N/P ratio of 1.4. All cell assembly procedures were conducted in an argon-filled glovebox (O_2_, H_2_O < 0.1 ppm). Based on the measured Young’s modulus (~10.1 GPa) of the carbonized electrode as shown in Figure S14, the applied stacking pressure during cell fabrication was set to 8 MPa, which is well below the elastic limit of the electrode material. This ensures reliable interfacial contact while preventing any structural damage during assembly. For pouch cell fabrication, a patterned sulfur cathode (S3 type, 2.2 cm × 2.2 cm) was hot-pressed onto an aluminum current collector. A lithium-coated copper foil of identical size was used as the anode. An Al_2_O_3_ coated Celgard PP separator was placed between the cathode and anode, and 480 μL of the aforementioned electrolyte was injected into the stack. The assembly was vacuum sealed in an argon atmosphere. Pouch cells were tested under a constant external pressure of 100 kPa using a custom-designed pouch cell holder.

Electrochemical tests were conducted using a VersaSTAT3 electrochemical workstation (Princeton Applied Research) and a Neware CT-4000 battery testing system at room temperature. Electrochemical Impedance Spectroscopy (EIS) measurements were performed in the frequency range of 100 kHz to 0.01 Hz using a 10 mV AC amplitude. Cyclic Voltammetry (CV) curves were recorded in the voltage range of 1.7-2.8 V (vs. Li^+^/Li) at scan rates ranging from 0.1 to 0.5 mV s^-1^. Galvanostatic charge-discharge tests were carried out at current densities from 0.1 C to 5 C (1 C = 1166 mA g^-1^). Electrodes for symmetric cell and Li_2_S precipitation/dissolution experiments were prepared using the same fabrication method as described above, except Li_2_SO_4_ was excluded from the precursor formulation. For symmetric cells, two identical Li_2_SO_4_-free electrodes were used as both cathode and anode. The electrolyte comprised 0.2 M Li_2_S_6_ and 1 M LiTFSI in a 1:1 (v/v) mixture of DME/DOL, with 1 wt% LiNO_3_ additive. Celgard PP separators were employed. CV measurements were performed in the range of −0.8 to 0.8 V at a scan rate of 5 mV s⁻¹. For Li_2_S precipitation and dissolution tests, Li_2_SO_4_-free electrodes were paired with lithium metal anodes and separated by Celgard PP membranes. The electrolyte contained 0.2 M Li_2_S_8_ and 1 M LiTFSI in DME/DOL (1:1 v/v), with 1 wt. % LiNO_3_. Cells were galvanostatically discharged at 0.1 mA to 2.06 V, followed by a potentiostatic discharge step at 2.05 V until the current declined below the cutoff threshold to evaluate the Li_2_S precipitation. For the Li_2_S dissolution, cells were first discharged to 1.7 V at 0.1 mA, then charged potentiostatically at 2.4 V.

**Morphology and chemo-physical characterization**

The chemical and physical properties of the fabricated electrodes were characterized by Fourier-transform infrared spectroscopy (FTIR, Nexus 670 ThermoNicolet Spectrometer). The crystallographic information of the fabricated electrodes was analyzed on a Panalytical Empyrean Powder X-ray diffractometer with Cu Kα radiation. The particle surface elemental and valence states were examined by X-ray photoelectron spectroscopy (Thermo Fisher Scientific NEXSA). A scanning electron microscopy (SEM) system (FEI Nova NanoSEM) and a transmission electron microscope (TEM) (JEOL 2010) were employed to investigate the morphology and element information of the sample. The cycled Li metal anode was rinsed with dimethyl carbonate (DMC) before SEM characterization to remove the residual lithium salt. Thermogravimetric analysis (TGA) was performed on a Pyris Diamond TG-DTA instrument in a nitrogen atmosphere. The specific surface area and pore size distribution were calculated by the Brunauer-Emmett-Teller (BET) method using a Micromeritics ASAP2020 instrument. Raman spectroscopy was conducted on an In Via Qontor confocal micro-Raman spectrometer (Renishaw, UK) to characterize the graphitic degree of carbon materials. In-situ experiments were obtained using a self-made cell, of which the schematic diagram of the in-situ cell device is shown in Figure S21.

**Computational Methods**

**Density functional theory (DFT)**

DFT calculations were performed using the Vienna Ab initio Simulation Package (VASP).^[2]^ The projector augmented wave (PAW) method was employed to describe the interaction between core and valence electrons.^[3]^ Electron exchange-correlation interactions were treated within the generalized gradient approximation (GGA) using the Perdew-Burke-Ernzerhof (PBE) functional.^[4]^ A plane-wave energy cutoff of 450 eV was adopted. The Brillouin zone was sampled using a Monkhorst-Pack k-point grid of 2 × 1 × 1. The electronic energy was considered converged when the total energy change fell below 10^-4^ eV, and the structures were relaxed until the residual forces on each atom were less than 10^-2^ eV Å^-1^. DFT-D3 of dispersion correction was adopted to describe the van der Waals (vdW) interactions.

The adsorption energy ($E_{ads}$) was calculated to evaluate the interaction strength between the substrate and sulfur species according to the following equation:

$$E_{ads}=E_{total}-(E_{sub}+E_{LiPSs})$$

where $E_{total}$ represents the total energy of the substrate-polysulfide adsorption system, $E_{sub}$ is the energy of the isolated substrate, and $E_{LiPSs}$ denotes the energy of the isolated polysulfide.

**Finite Element Analysis (FEA)**

FEA simulations of the Li-S battery were conducted using COMSOL Multiphysics 6.1. The column geometry represents a *periodic unit cell* to capture through-thickness gradients to provide a physically meaningful and computationally efficient unit for capturing the through-thickness of the electrode structure. The governing equations included ionic transport, electrochemical kinetics, and mechanical deformation. The transport of ionic species in the liquid phase was modeled using the Nernst-Planck equation, accounting for both diffusion and migration driven by concentration gradients and electric fields:

$$N_{i,m}=-D_{i,m}\times({\nabla C}_{i}^{b}+\frac{z_{i}FC_{i}^{b}}{RT}{\nabla\phi}_{e})$$

Here, $D_{i,m}$ is the diffusion coefficient of species $i$ ($i={Li}^{+}, {{Li}_{2}S}_{(l)}, {S^{2-}, S}_{2}^{2-}, S_{4}^{2-}, S_{6}^{2-}, S_{8}^{2-}$) and $m$ corresponds to the domain (separator or cathode). $N_{i,m}$ denotes the ion flux, $C_{i}^{b}$ is the species concentration, $z_{i}$ is the charge number of the species $i$, and $\phi_{e}$ is the electrolyte potential. On the cathode side, solid sulfur ${{Li}_{2}S}_{(s)}$ dissolves into the electrolyte to form ${{Li}_{2}S}_{(l)}$, which undergoes a cascade of electrochemical reductions to form lower-order LiPSs:

$${{{{Li}_{2}S}_{(s)}\leftrightarrow Li}_{2}S}_{(l)}$$

$$S^{2-}\leftrightarrow\frac{1}{2}S_{2}^{2-}+e^{-}$$

$$S_{2}^{2-}\leftrightarrow\frac{1}{2}S_{4}^{2-}+e^{-}$$

$$\frac{3}{2}S_{4}^{2-}\leftrightarrow S_{6}^{2-}+e^{-}$$

$${2S}_{6}^{2-}\leftrightarrow\frac{3}{2}S_{8}^{2-}+e^{-}$$

$${\frac{1}{2}S}_{8}^{2-}\leftrightarrow S_{8(l)}+e^{-}$$

$$S_{8(l)}\leftrightarrow S_{8(s)}$$

And the reaction process of Li-ions at the interface of the electrolyte and the anode can be described by the simplified reaction:

$${Li}^{+}+e^{-}\leftrightarrow Li$$

The local charge transfer rate for each electrochemical reaction $j$ was modeled using the Butler-Volmer equation:

$$i=i_{ex,j}[\prod\left( \frac{C_{i}}{C_{i,ref}} \right)^{pi,j}\exp\left( \frac{\alpha_{a,j}F_{\eta_{j}}}{RT} \right)-\prod\left( \frac{C_{i}}{C_{i,ref}} \right)^{qi,j}\exp\left( \frac{\alpha_{c,j}F_{\eta_{j}}}{RT} \right)]$$

where $i_{ex,j}$ is the exchange current density, $\alpha_{a,j}$ and $\alpha_{c,j}$ are the anodic and cathodic transfer coefficients, $C_{i,ref}$ is the reference concentration, and $C_{i}$ is the interfacial concentration of species $i$. The overpotential, $\eta_{j}$, was calculated by:

$$\eta=\phi_{s}-\phi_{e}-U_{eq}$$

where $\phi_{s}$ and $\phi_{e}$ are the solid phase and electrolyte potentials, and $U_{eq}$ is the equilibrium potential. The open-circuit potential for each reaction $j$ at $C_{i,ref}$ was determined using the Nernst equation:

$$U_{j,ref}=U_{j}^{\theta}-\frac{RT}{n_{j}F}\sum\left\{ S_{i,j}\ln\left[ \frac{C_{i,ref}}{1000} \right] \right\}$$

where $U_{j}^{\theta}$ is the standard equilibrium potential, and $C_{i,ref}$ represents the reference concentrations, taken to be the initial species concentrations. The precipitation of $S_{8(s)}$ and ${{Li}_{2}S}_{(s)}$ is governed by the following expression:

$$R_{k}=K_{k}\left( \prod_{i}C_{i}^{\gamma_{i,k}}-K_{sp,k} \right)$$

where $R_{k}$ represents the precipitation rate of the solid species ($k=S_{8(s)}$, ${{Li}_{2}S}_{(s)}$), $K_{sp,k}$ is the solubility product of species in the electrolyte, and $K_{k}$ denotes the rate constant. $\gamma_{i,k}$correspond to the number of moles of ionic constituents $i$ and the solid species $k$. To simulate mechanical constraints, fixed boundary conditions ($u=0$) were applied to both ends of the cell to mimic the effect of external fixtures. Other surfaces were modeled with roller boundary conditions ($u\times n=0$), allowing in-plane movement. The cathode surface was assigned a normal displacement to account for the precipitation of $S_{8(s)}$, governed by the surface-normal velocity $u_{n}$:

$$u_{n}=AR_{Li_{2}S}\frac{M}{\rho}$$

where $M$ and $\rho$ are the molar mass and density of $S_{8(s)}$, respectively, and $A$ is the surface area. The stress-strain relationship followed Hooke’s Law:

$$\sigma_{i,j}=\frac{E}{1+\upsilon}\nabla\vec{\mathcal{l}}+\frac{2\upsilon E}{1-2\upsilon}$$

where $E$ is the Young’s modulus, $\nabla\vec{\mathcal{l}}$ is the displacement of the cathode-electrolyte interface, and $\upsilon$ is Poisson’s ratio.

Simulations presented in Figure 1 employed unified COMSOL-based parameters to isolate the effect of structural geometry among S1-S3. In contrast, simulations in Figure 4 were performed using experimentally calibrated, electrode-specific parameters listed in Table S1, enabling quantitative comparison with the measured electrochemical performance and mechanical response. Specifically, diffusion coefficients were determined from cyclic voltammetry (Randles-Sevcik analysis) and scaled from the COMSOL model. Reaction rate constants were fitted using Tafel slopes and symmetric-cell polarization curves. Li_2_S/S_8_ precipitation-dissolution kinetics were refined by matching potentiostatic nucleation/dissolution (I-t) profiles. Young’s modulus values were obtained from nanoindentation/micro-compression experiments (Figure 5e).

| Parameter | Default value from COMSOL | Value scaled in this work | Unit | Source/Calibration method |
| --- | --- | --- | --- | --- |
| DLi+^+^ | 0.88E-12 | 3.2E-12 (S1), 7.4E-12, 1.2E-11 | m^2^ s^-1^ | CV-based Randles-Sevcik fitting |
| DS_8_^2-^ | 3.5E-12 | 1.2E-11 (S1), 2.9E-11 (S2), 4.8E-11 (S3) | m^2^ s^-1^ | Scaled from D_Li+_ |
| i0,1 | 1.9 | 1.1 (S1), 2.1 (S2), 3.5 (S3) | A m^-2^ | Fitted from Tafel slopes |
| i0,4 | 2E-4 | 1.1E-4 (S1), 2.2 E-4 (S2), 3.7E-4 (S3) | A m^-2^ | Scaled from i0,1 |
| kLi_2_S | 3.45E-5 | 5.7E-5 (S1),  8.4E-5 (S2),  1.2E-4 (S3) | m^6^ mol^-1^ s^-1^ | Fitting to potentiostatic I-t |
| kS_8_ | 5 | 4.1 (S1), 6.7 (S2),  7.9 (S3) | s^-1^ | Fittted to dissolution branch |

Table S1. Summary of simulation parameters and calibration sources.

**Supplementary Notes**

**Note 1** | Calculation of relative intensity (RI) for solid-phase quantification in in-situ XRD and for soluble LiPSs using in-situ Raman spectroscopy

To quantitatively analyze the evolution of solid phases during the charge-discharge process, the intensities of the (111) diffraction peak of Li_2_S and the (311) diffraction peak of S_8_, which represent the most prominent peaks of their respective crystalline phases, were selected for analysis. These intensities were normalized to the initial intensity of the Li_2_S (111) peak at the beginning of charge, serving as a reference for calculating the $RI$ throughout the cycle. The normalization was performed using the following equation:

$$RI=\frac{I_{m}^{t}}{I_{Li_{2}S}^{0}}$$

where $I_{Li_{2}S}^{0}$ represent is the initial intensity of the Li_2_S (111) peak at the start of charging, $I_{m}^{t}$ is the intensity of $m$ ($m$ = $Li_{2}S$, $S_{8}$) at time $t$. The evolution of the relative intensities of these two reflections during the charge-discharge process for all three electrodes is plotted in Figure 4b.

**Note 2** | Calculation of relative intensity (RI) for soluble LiPSs using in-situ Raman spectroscopy

To quantitatively track the evolution of soluble lithium polysulfides (LiPSs) during the charge-discharge process, characteristic Raman peaks corresponding to Li_2_S_4_ (~450 cm^-1^), Li_2_S_6_ (~400 cm^-1^), and Li_2_S_8_ (~152 cm^-1^) were selected as representative indicators. The intensity of each of these peaks was normalized to the intensity of the LiTFSI peak at ~282 cm^-1^, which originates from the electrolyte and serves as an internal standard due to its relative stability throughout the cycling process. The $RI$ of each LiPSs species was calculated using the following equation:

$$RI=\frac{I_{n}^{t}}{I_{LiTFSI}^{t}}$$

where $I_{LiTFSI}^{t}$ represent is the initial intensity of the LiTFSI peak at the same state, $I_{n}^{t}$ is the peak intensity of selected LiPSs $n$ ($n$ = $Li_{2}S_{4}$, $Li_{2}S_{6}$*,* $Li_{2}S_{8}$) at time $t$. The temporal evolution of each soluble LiPSs species during the charge and discharge processes is plotted in Figure 4d.

**Supplementary Figures and Tables**


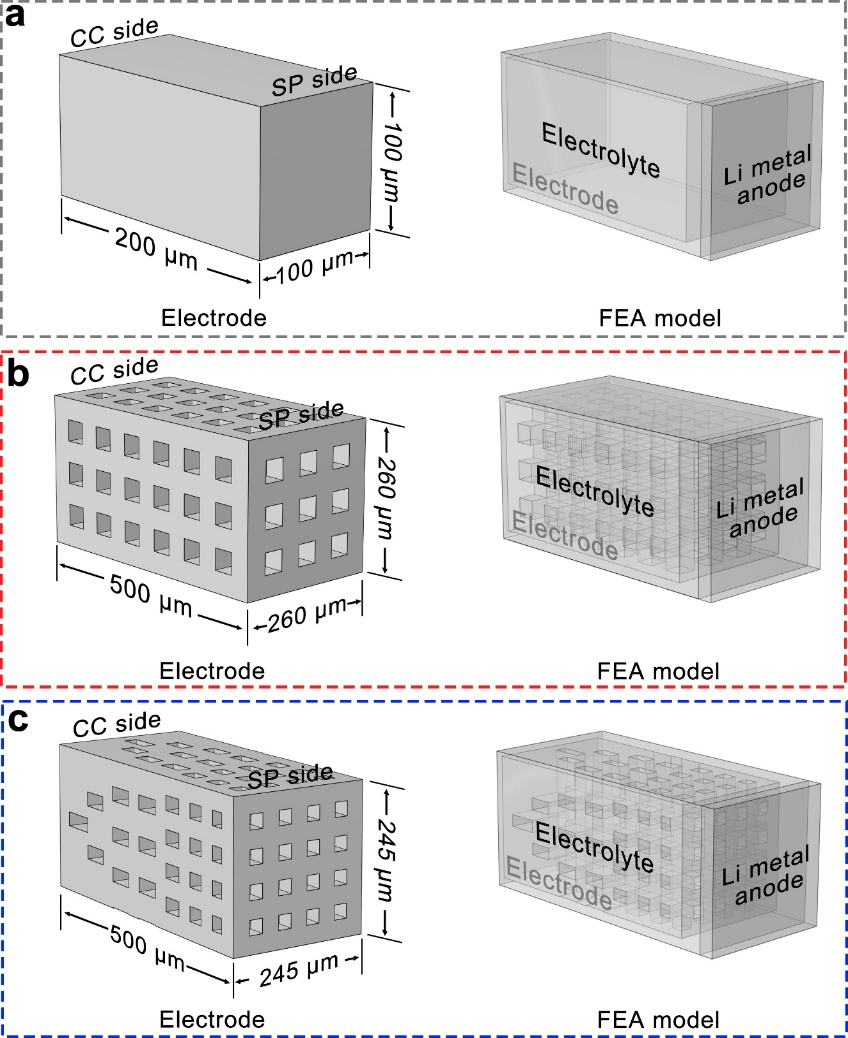


Figure S1. Structural schematics of (a) S1, (b) S2, and (c) S3 electrodes alongside their corresponding FEA models.

The S1 electrode features a conventional two-dimensional planar architecture with a thickness of 200 μm. The S2 electrode incorporates a uniform three-dimensional channel array extending through the thickness (500 μm), designed to reduce tortuosity and enhance ion transport. The S3 electrode adopts a dual-gradient architecture, with pore size and areal active material concentration varying along the thickness (500 μm) to balance Li-ion concentration and reaction kinetics between the SP and CC sides. The FEA models couple the electrode structure with the electrolyte and lithium metal anode to simulate electrochemical-mechanical behavior during cycling.


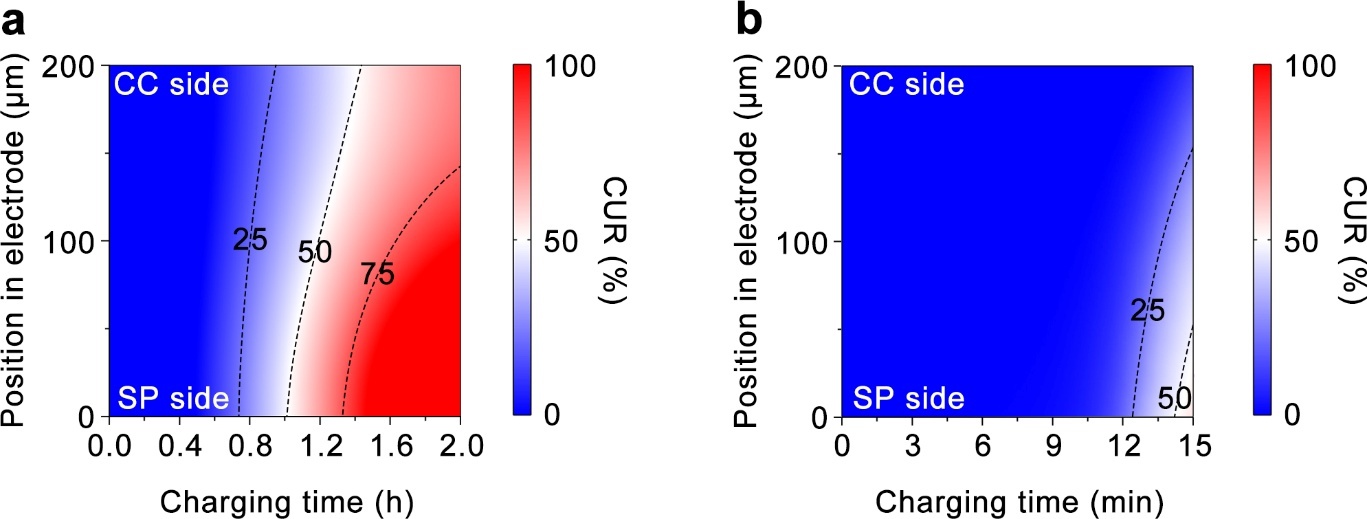


Figure S2. CUR distribution along the thickness direction of the S1 electrode as a function of charging time under a current rate of (a) 0.5 C and (b) 4 C. Note that the x-axis time scale is in hours in (a) and in minutes in (b).

Figure S2 shows the simulated CUR distribution along the thickness of the S1 electrode during charging at 0.5 C and 4 C. At 0.5 C (Figure S2a), the CUR decreases progressively from the SP side toward the CC side as charging proceeds. By the end of charging (2.0 h), the SP side reaches full utilization, while the CC side remains at a much lower CUR, indicating incomplete lithiation of the bottom region toward CC. When the charging current further increasing to 4 C (Figure S2b), this disparity becomes more pronounced: after 15 minutes of charging, the SP side achieves high utilization, but the CC-side region deeper than ~50 μm remains below 50% CUR. These results indicate that thick planar electrodes experience significant concentration polarization and hinder Li^+^ transport, especially under high-rate charging, which ultimately reduces overall cathode utilization.


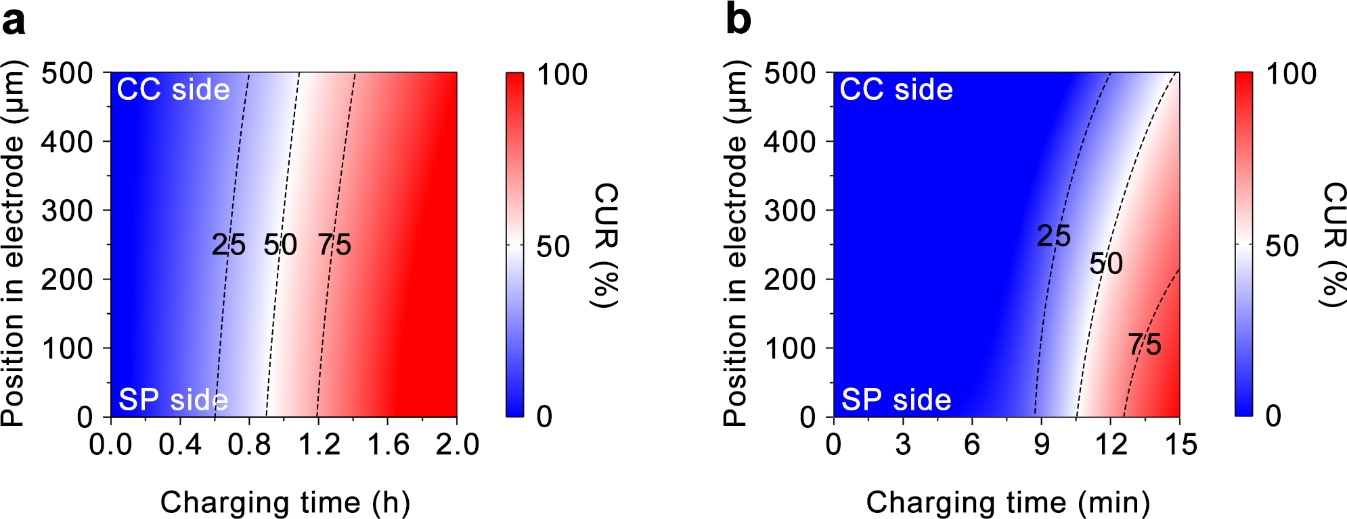


Figure S3. CUR distribution along the thickness direction of the S2 electrode as a function of charging time under a current rate of (a) 0.5 C and (b) 4 C. Note that the x-axis time scale is in hours in (a) and in minutes in (b).

As shown in Figure S3a, the SP side reaches 25%, 50%, and 75% of CUR earlier than the CC side. At 4 C (Figure S3b), the SP side again reaches high utilization quickly, but a pronounced CUR gradient persists, and the CC-side region beyond ~200 μm depth remains below 75% CUR at the end of the 15 min charge. These results indicate that while the 3D uniform structure of the S2 electrode improves Li^+^ transport compared to planar electrode designs, concentration polarization still limits uniform cathode utilization at high charging rates.


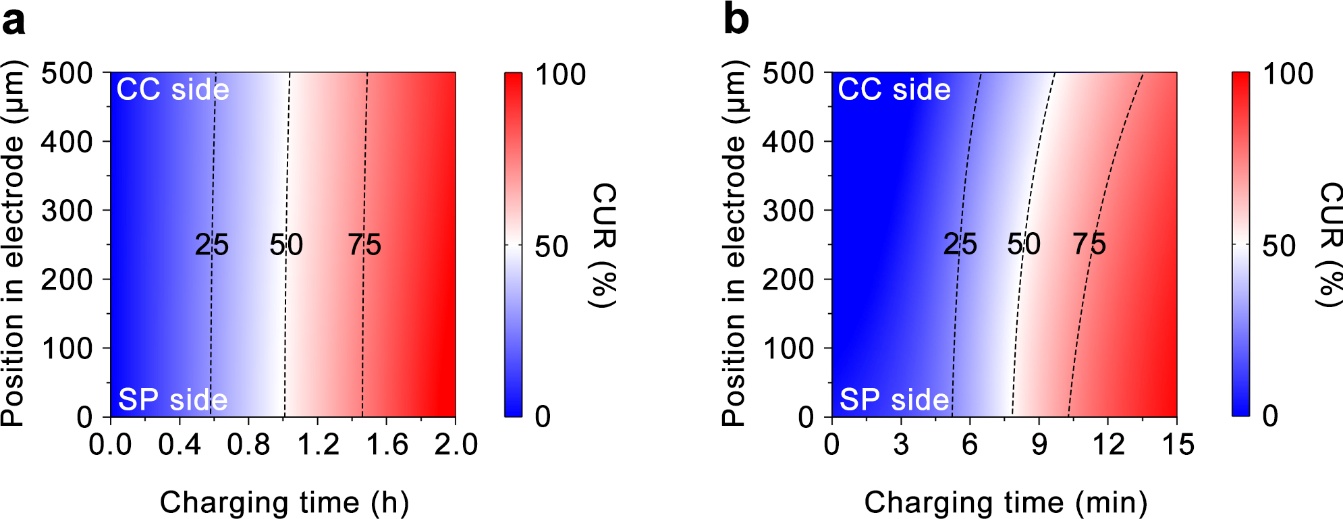


Figure S4. CUR distribution along the thickness direction of the S3 electrode as a function of charging time under a current rate of 0.5 C and 4 C. Note that the x-axis time scale is in hours in (a) and in minutes in (b).

As shown in Figure S4a, the SP and CC sides exhibit nearly synchronized utilization at the same time under 0.5 C. At 4 C (Figure S4b), the CUR distribution remains highly uniform, and even the CC-side region (i.e., entire cathode thickness) achieves over 75% utilization by the end of the 15 min charge. This demonstrates that the correlated dual-gradient design of the S3 electrode effectively minimizes concentration polarization, ensuring balanced Li^+^ distribution and high utilization across the entire electrode, even at high charging rates.


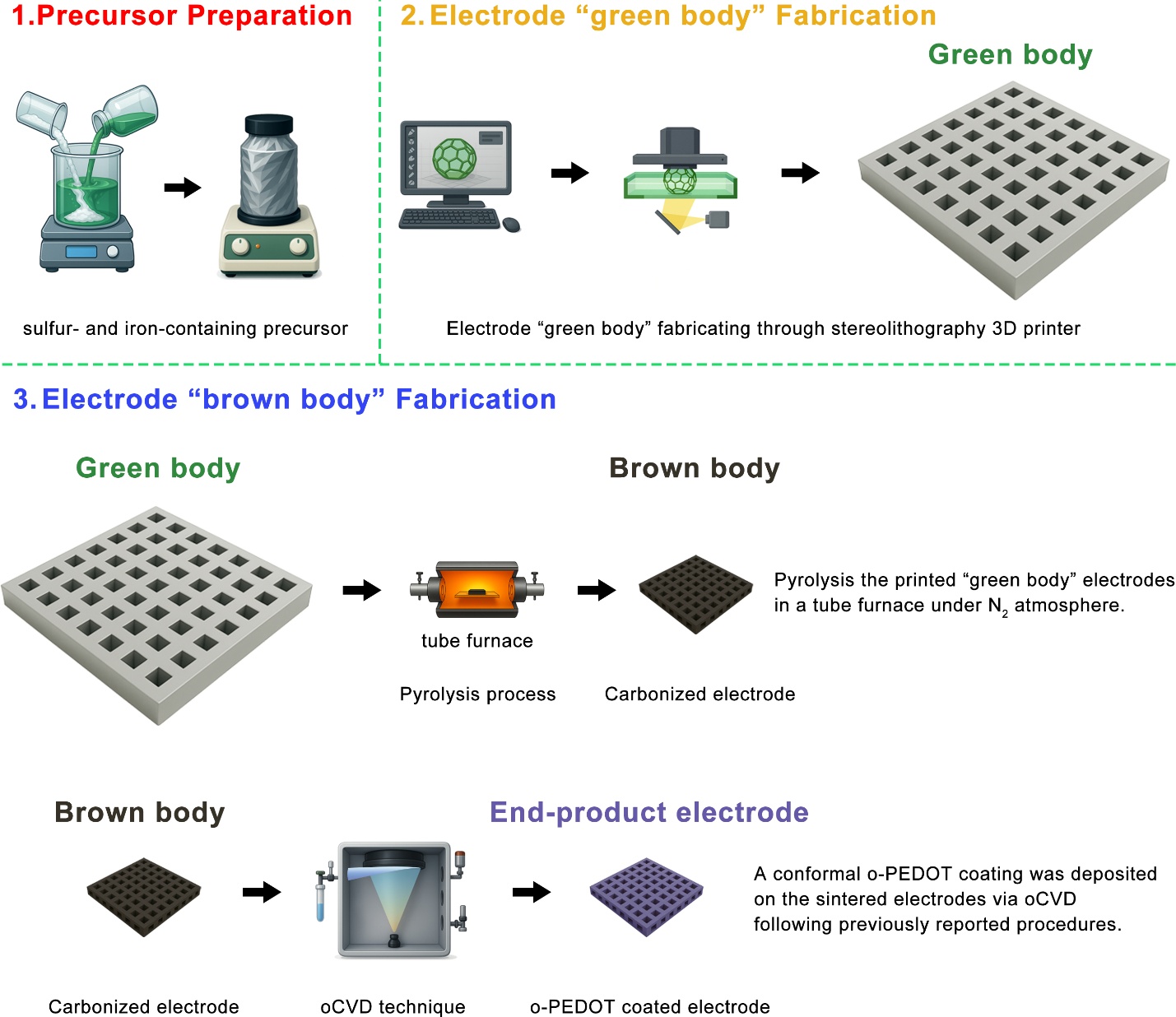


Figure S5. Schematic illustration of the electrode manufacturing process.

Figure S5 schematically illustrates the electrode manufacturing process. (1) Precursor preparation: Li_2_SO_4_, Fe(NO_3_)_3_·9H_2_O, and triallyl-1,3,5-triazine-2,4,6-(1H,3H,5H)-trione were mixed in a beaker under magnetic stirring. After homogenization, 2,2-dimethoxy-2-phenylacetophenone was added as a photoinitiator, and the container was wrapped with Al foil to minimize light exposure. (2) Electrode “green body” fabrication: The electrode architecture was designed and sliced using CAD software and then fabricated by stereolithography (SLA) 3D printing. (3) Electrode “brown body” fabrication: The printed “green body” electrodes were carbonized via pyrolysis in a tube furnace under N_2_ atmosphere. (4) End-product electrode: A conformal o-PEDOT coating was deposited onto the carbonized electrodes using oCVD following previously reported protocols. More detailed procedures are provided in the Experimental section.


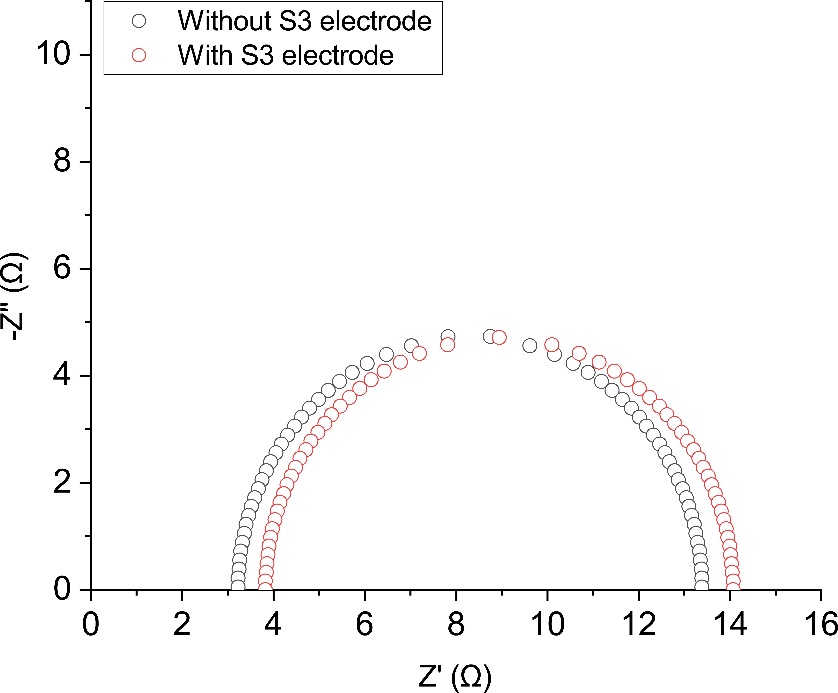


Figure S6. Nyquist plots of a dry coin cell with and without the S3 electrode.

Figure S6 shows the Nyquist plots of dry coin cells assembled without electrolyte, with two different configurations: steel|S3 electrode|steel and steel|steel. The resistance values were extracted from the two x-axis intercepts of the semicircle in the Nyquist plots. The measured resistances of the cells are 10.651 Ω for the cell with the S3 electrode and 10.659 Ω for the blank cell, yielding a difference of 0.008 Ω attributable to the S3 electrode. Based on this resistance value, the electronic conductivity (σ) of the S3 electrode was calculated using σ = L/(R·S), where L is the electrode thickness (~470 μm) and S is the electrode area (0.88 cm2). The resulting conductivity is 6.7 S cm^-1^.


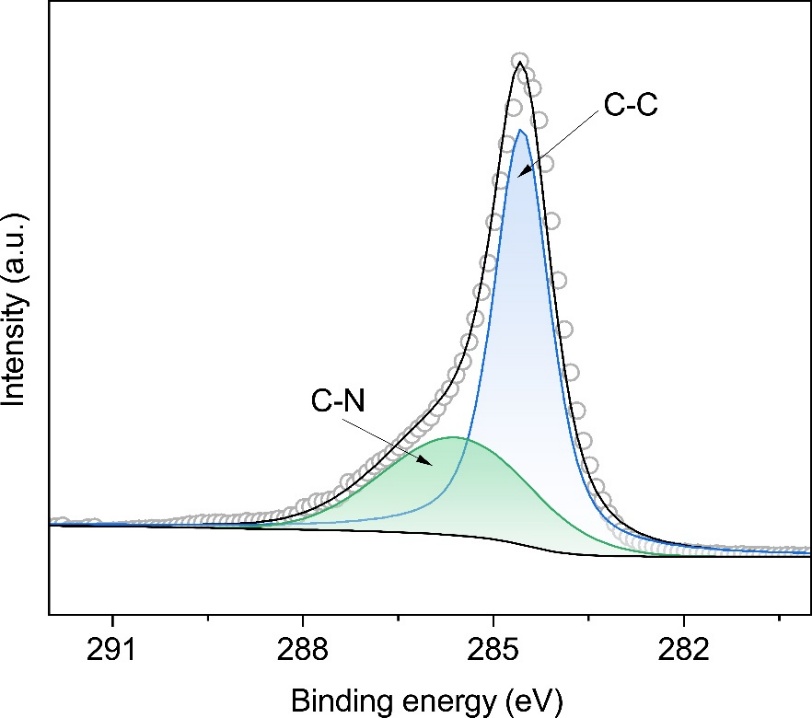


Figure S7. High-resolution XPS spectrum of the C 1s region

Figure S7 presents the high-resolution XPS spectrum of the C 1s region. An asymmetry peak can be observed at around 284.5 eV, which can be deconvoluted into two main components, with the dominant peak at 284.5 eV corresponding to C-C bonding and a smaller peak at 285.8 eV attributed to C-N bonding, formed during the N_2_-ambient pyrolysis. The asymmetry of the HR C 1s XPS (due to C-N component peak) further complements the validation of the incorporation of nitrogen into the carbon framework.


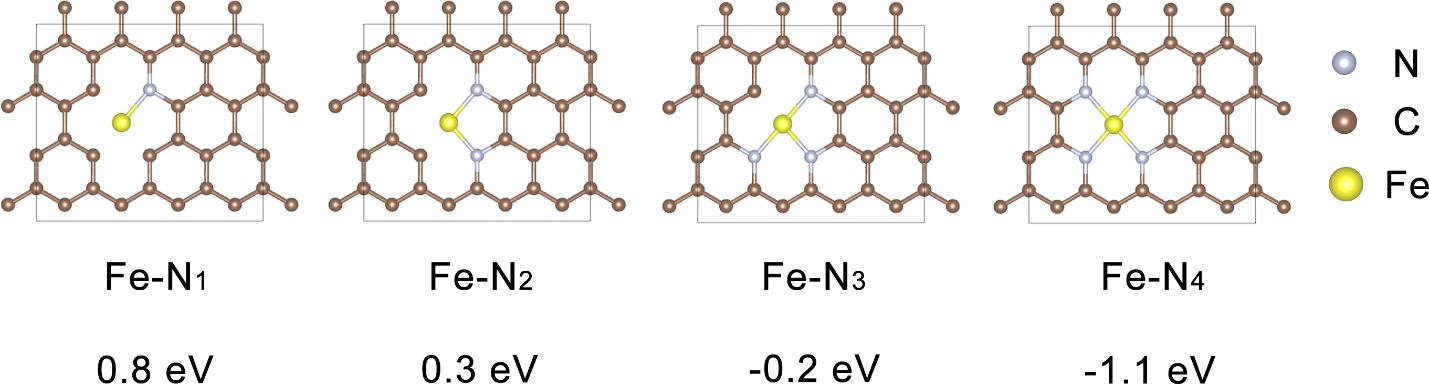


Figure S8. Formation energy of Fe-N_1_, Fe-N_2_, Fe-N_3_, and Fe-N_4_.

To further identify the most thermodynamically stable coordination environment of Fe-Nx, DFT calculations were performed to evaluate the formation energies of Fe-N_1_, Fe-N_2_, Fe-N_3_, and Fe-N_4_ complexes. As illustrated in Figure S8, the corresponding formation energies are 0.8 eV, 0.3 eV, -0.2 eV, and -1.1 eV, respectively. The positive formation energies of Fe-N_1_ and Fe-N_2_ indicate that these configurations are thermodynamically unstable. In contrast, Fe-N_4_ exhibits the lowest formation energy among all models, suggesting the highest thermodynamic stability. Therefore, the experimentally observed Fe-N complex is most likely coordinated in an Fe-N_4_ configuration.


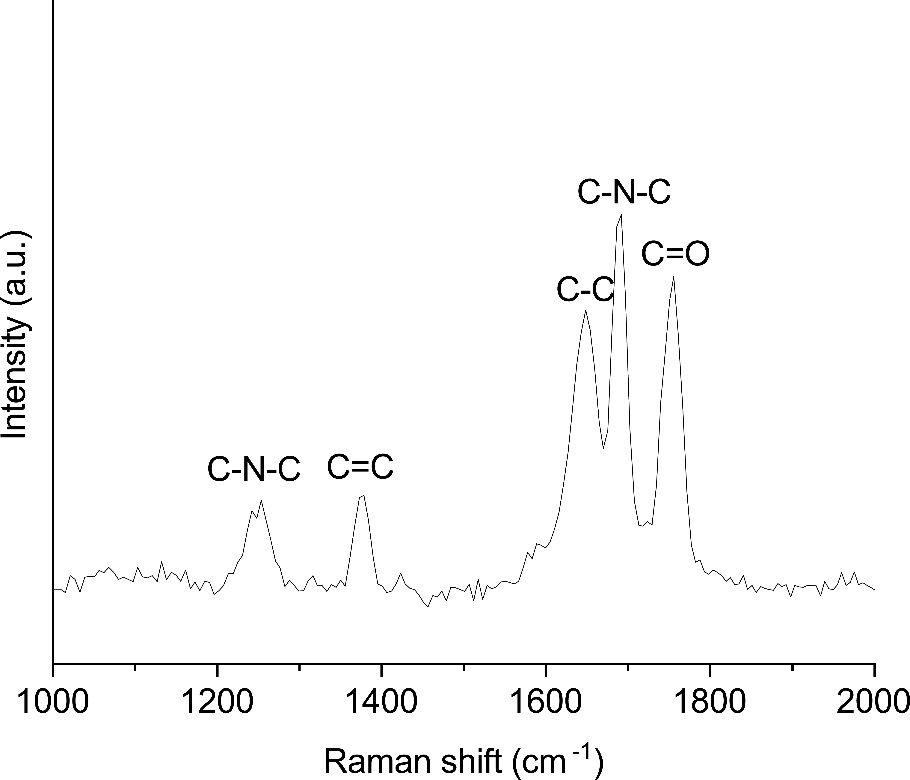


Figure S9. Raman spectrum of the electrode precursor before pyrolysis

Figure S9 presents the Raman spectrum of the electrode precursor poly-(TTT) before pyrolysis. Characteristic peaks of poly-(TTT) are observed at 1250 and 1690 cm^-1^, corresponding to the C-N-C symmetric stretching vibration of the poly-(TTT) skeleton. The peak at 1370 cm^-1^ is assigned to the C=C vibration of poly-(TTT), while the band at 1750 cm^-1^ originates from the C=O group. In addition, the peak at 1650 cm^-1^ corresponds to C-C bonds, confirming the successful polymerization of the TTT monomer. After pyrolysis, all these molecular features disappear, and only the typical carbon D-band and G-band (Figure 2c in the main text) remain, indicating the transformation into a carbonaceous framework. The disappearance of nitrogen- and oxygen-related vibrations is attributed to the release of volatile (i.e., gaseous) species such as NO_x_, CO, and CO_2_ during high-temperature pyrolysis. Nonetheless, non-volatile nitrogen functionalities are retained within the carbon lattice, as later verified by XPS (C-N bonding), contributing to heteroatom doping of the carbon matrix.


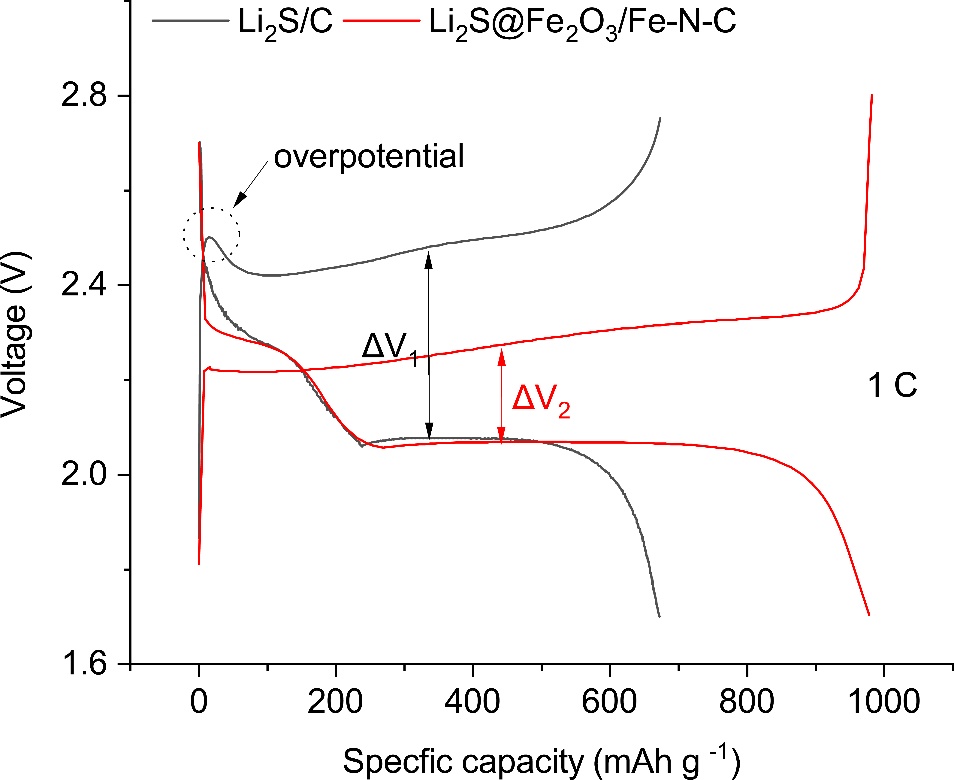


Figure S10. Voltage-capacity profiles of Li_2_S/C and Li_2_S@Fe_2_O_3_/Fe-N-C cathodes under 1 C.

Figure S10 compares the voltage-capacity profiles of Li_2_S/C and Li_2_S@Fe_2_O_3_/Fe-N-C cathodes under 1 C. Both electrodes were fabricated by blade casting with a Li_2_S loading of approximately 2.2 mg cm^-2^, as described in the Experimental Section. For the Li_2_S/C electrode, a pronounced overpotential is observed near 2.5 V during the charging process, indicating sluggish reaction kinetics. The voltage gap (ΔV_1_) is 0.42 V, corresponding to significant electrochemical polarization caused by the high reaction barrier. The capacity ratio (Q_L_/Q_H_) is 1.82, and the specific capacity reaches 673 mAh g^-1^ at 1 C. In contrast, the Li_2_S@Fe_2_O_3_/Fe-N-C electrode shows no obvious overpotential during charging, with a smaller voltage gap (ΔV_2_) of 0.21 V. The Q_H_/Q_L_ value increases to 2.75, and the specific capacity reaches 981 mAh g^-1^. These results indicate that Fe_2_O_3_/Fe-N-C effectively reduces the reaction barrier, improves redox kinetics, and alleviates electrochemical polarization under moderate sulfur loading conditions.


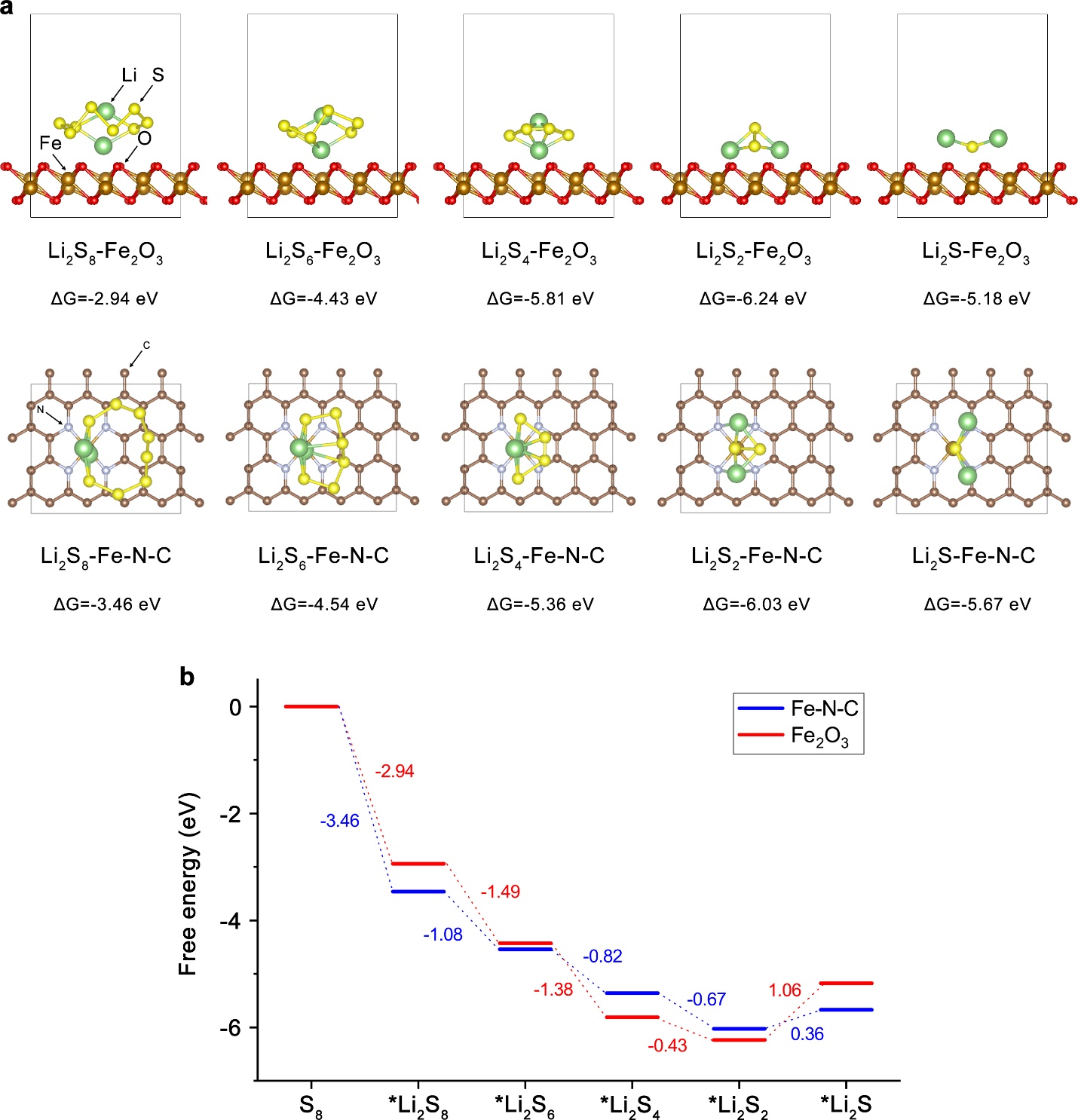


Figure S11. (a) Optimized structures of Li polysulfides (Li_2_S_8_, Li_2_S_6_, Li_2_S_4_, Li_2_S_2_, and Li_2_S) on Fe₂O₃ and Fe-N-C substrates, along with their corresponding Gibbs free energies (ΔG). (b) Free energy profiles of the Li polysulfide conversion process on Fe-N-C and Fe2O3, showing the stepwise changes in Gibbs free energy from S₈ to Li₂S.

DFT calculations were performed to evaluate the Gibbs free energies (ΔG) of lithium polysulfide intermediates (Li_2_S_8_, Li_2_S_6_, Li_2_S_4_, Li_2_S_2_, and Li_2_S) on Fe_2_O_3_ and Fe-N-C surfaces. Figure S11a shows the optimized structures of these intermediates on the two substrates together with their calculated Gibbs free energies. The corresponding free energy profiles in Figure S11b illustrate the stepwise change from S_8_ to Li_2_S. Both catalytic surfaces exhibit a gradual decrease in Gibbs free energy with successive reduction of LiPSs, confirming the thermodynamic feasibility of the conversion pathway. The comparison further indicates distinct energetic features between Fe_2_O_3_ and Fe-N-C, reflecting their different catalytic roles in regulating LiPSs transformations. Notably, Fe_2_O_3_/Fe-N-C exhibits a distinct energetic profile, Fe_2_O_3_ is more effective for the conversion of long- to medium-chain polysulfides, while Fe-N-C preferentially facilitates medium- to short-chain transformations as well as the solid-liquid (S_8_ to Li_2_S_8_) and liquid to solid (Li_2_S_2_ to Li_2_S), hereby highlighting their synergistic catalytic function.

|  | TiO_2_ | MnO_2_ | Fe_3_O_4_ | V-N-C | ZnO | Zn-N-C | Co_3_O_4_ | Co-N-C | N-C | Fe_2_O_3_/Fe-N-C |
| --- | --- | --- | --- | --- | --- | --- | --- | --- | --- | --- |
| Ref. | ^[5]^ | ^[6]^ | ^[7]^ | ^[8]^ | ^[9]^ | ^[10]^ | ^[11]^ | ^[10]^ | ^[12]^ | This work |
| S_8_ | 0 | 0 | 0 | 0 | 0 | 0 | 0 | 0 | 0 | 0 |
| *Li_2_S_8_ | -3.8 | -3.19 | -4 | -3.56 | -3.06 | -2.72 | -2.35 | -3 | -3.07 | -3.46 |
| *Li_2_S_6_ | 0.72 | -1.91 | -0.51 | 0.38 | 0.19 | 0.22 | -1.73 | 0.16 | 0.43 | -1.49 |
| *Li_2_S_4_ | -0.92 | -1.14 | -1.66 | 0.11 | -0.43 | 0.36 | 1.27 | 0.17 | 0.32 | -1.38 |
| *Li_2_S_2_ | 0.58 | -0.95 | -0.46 | 1.1 | 0.66 | 0.37 | -0.32 | 0.52 | 0.76 | -0.67 |
| *Li_2_S | 0.88 | 1.14 | 1.04 | 0.39 | 0.24 | 0.64 | -1.15 | 0.69 | 1.21 | 0.36 |

Table S2. Relative Gibbs free energies (ΔG, eV) of LiPSs on different catalytic materials. Reference values are taken from the literature, and the data for Fe_2_O_3_/Fe-N-C are from this work.

Table S2 summarizes the relative Gibbs free energies (ΔG) of Li_2_S_8_, Li_2_S_6_, Li_2_S_4_, Li_2_S_2_, and Li_2_S on various catalytic surfaces, including TiO_2_, MnO_2_, Fe_3_O_4_, V-N-C, ZnO, Zn-N-C, Co_3_O_4_, Co-N-C, N-C, and Fe_2_O_3_/Fe-N-C. The reference values for previously reported catalysts were extracted from the literature, while the data for Fe_2_O_3_/Fe-N-C were obtained in this work. The free energy values provide a direct comparison of the thermodynamic stability of polysulfide intermediates across different catalysts, offering insights into their relative ability to regulate the stepwise conversion from S_8_ to Li_2_S. Compared with the other catalysts, Fe_2_O_3_/Fe-N-C uniquely enables synergistic regulation of both long-chain and short-chain polysulfide transformations.


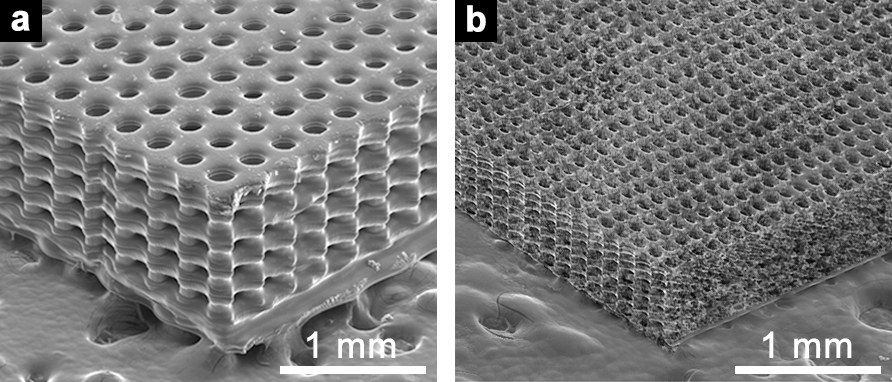


Figure S12. SEM images of the S2 electrode before and after pyrolysis.

Figure S12 shows SEM images of the S2 electrode before and after pyrolysis. The as-printed electrode (Figure S12a) exhibits a well-defined periodic structure with smooth surfaces and uniform pores. After pyrolysis (Figure S12b), the electrode retains its overall 3D-printed architecture while the surface becomes rougher with nano-sized pores, compared to the smoother surface before pyrolysis, reflecting structural evolution during the thermal treatment. After pyrolysis, the precursor framework in Figure S12a was effectively converted into a conductive carbon-based skeleton while preserving the designed macroscopic architecture.


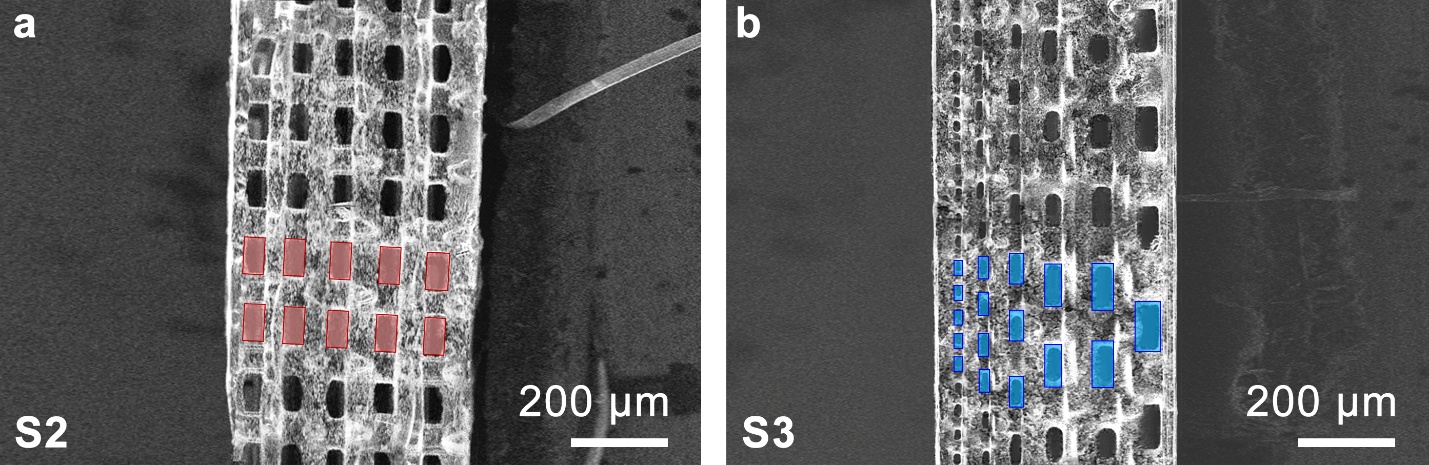


Figure S13. Cross-sectional images of (a) S2 electrode and (b) S3 electrode after pyrolysis.

As annotated in Figure S13a, the S2 electrode displays uniformly sized and periodically arranged microscale pores (marked by red rectangles), consistent with its designed uniform 3D architecture. In contrast, as indicated by the artificial blue-rectangle annotations in Figure S13b, the S3 electrode exhibits a clear and continuous increase in pore size from one side to the other, confirming the presence of the intended pore-size gradient. In addition, the cross-sectional images reveal that the S2 and S3 electrodes possess comparable overall thicknesses of approximately 490 μm and 485 μm, respectively. These results verify that the experimentally constructed S2 and S3 architectures accurately reflect their designed morphologies and remain structurally stable after pyrolysis.


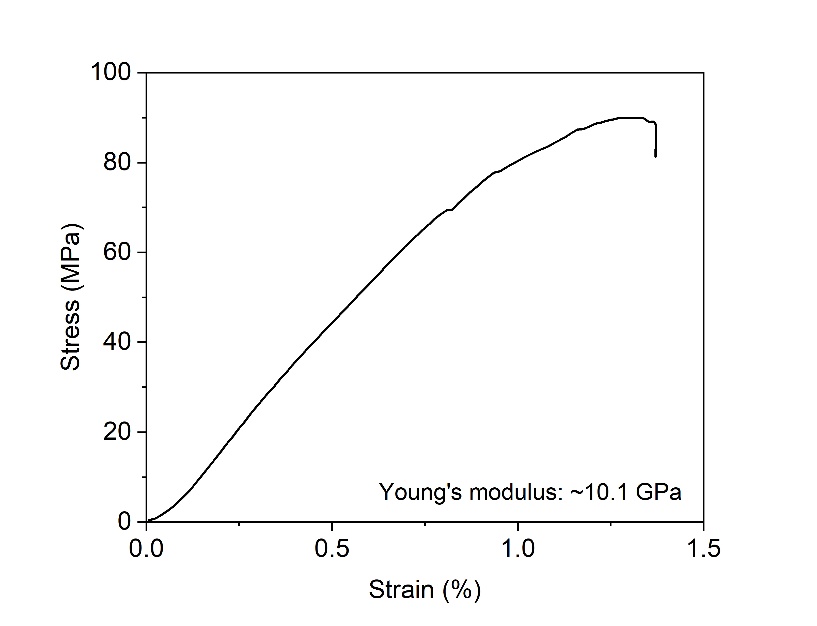


Figure S14. Stress-strain curve of the electrode after pyrolysis.

To evaluate the mechanical stiffness of the carbonized electrode and ensure that the applied pressure during cell assembly would not induce structural deformation, the Young’s modulus of the electrode after pyrolytic carbonization was measured by nanoindentation using a Berkovich diamond tip (Hysitron TriboIndenter) under ambient conditions. The reduced modulus was derived from the unloading stiffness using the Oliver-Pharr method. As shown in Figure S14, the stress-strain curve exhibits a linear elastic region followed by gradual plastic deformation, yielding an average Young’s modulus of approximately 10.1 GPa. This indicates that the pyrolyzed electrode becomes mechanically stiffer due to the formation of a robust carbon framework. Based on this modulus, an assembly pressure of 8 MPa was selected during cell fabrication to ensure reliable interfacial contact while avoiding fracture or permanent deformation.


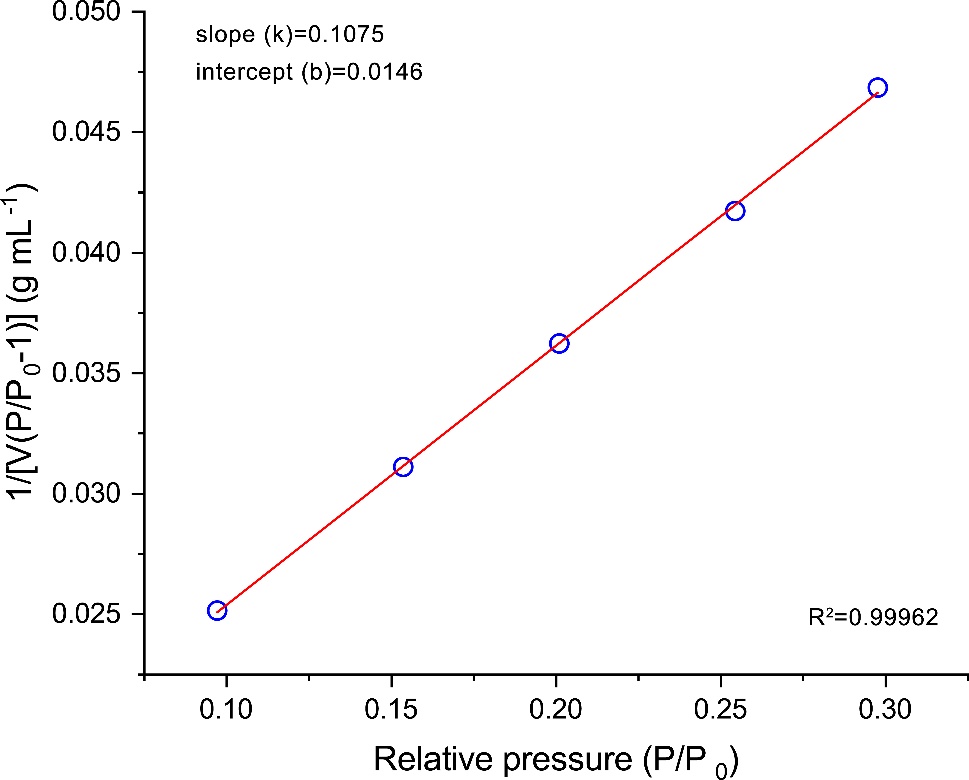


Figure S15. BET fitting curve of the electrode material after pyrolysis.

BET fitting of the electrode material after pyrolysis was performed, and the corresponding linear plot is shown in Figure S15. According to the Brunauer-Emmett-Teller (BET) equation^[13]^:

$$\frac{1}{V(\frac{P_{0}}{P}-1)}=\frac{C-1}{V_{m}C}\times\frac{P}{P_{0}}+\frac{1}{V_{m}C}$$

where $V$ is the adsorbed gas volume, $\frac{P}{P_{0}}$​ is the relative pressure, $V_{m}$​ is the monolayer adsorption volume, and $C$ is the BET constant. From the slope (k=0.1075) and intercept (b = 0.0146) in Figure S15, the monolayer volume can be obtained as:

$$V_{m}=\frac{1}{k+b}=8.19 {cm}^{3}g^{-1}$$

The specific surface area was then calculated using:

$$S_{BET}=\frac{V_{m}N_{A}\sigma}{V}$$

where $N_{A}$ is Avogadro’s number, $\sigma$ is the molecular cross-sectional area of N_2_ (0.162 nm^2^), and $V$ is the molar volume of gas at STP (22414 cm^3^ mol^-1^). The resulting BET specific surface area is:

$$S_{BET}=35.65 m^{2}g^{-1}$$

The excellent linear correlation (*R*^2^ = 0.99962) confirms the reliability of the fitting.


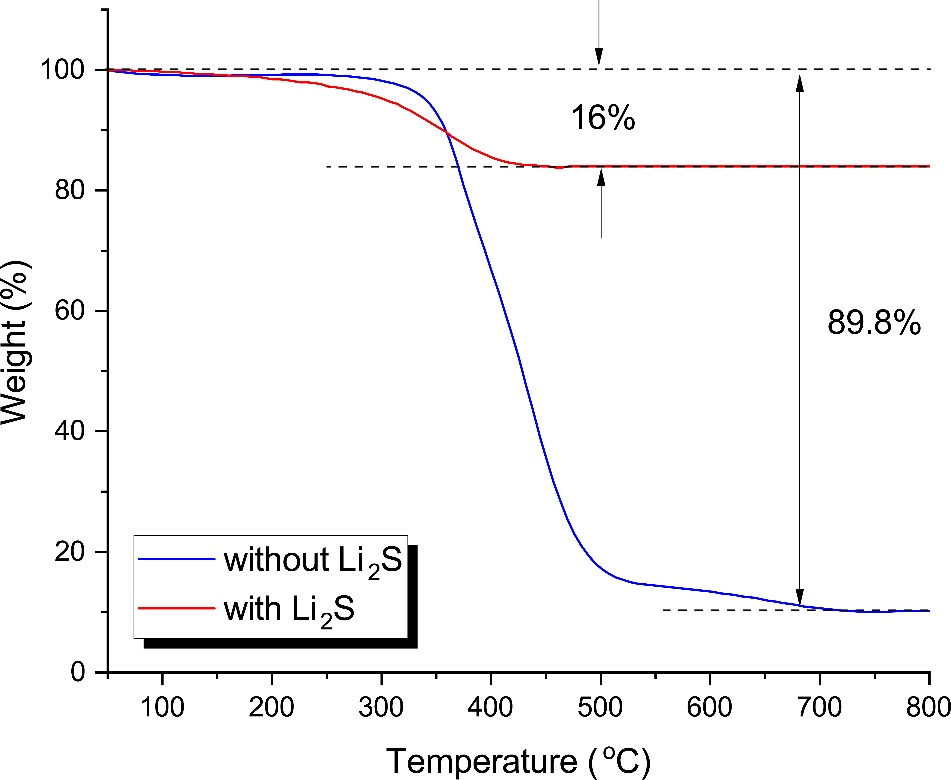


Figure S16. TGA (air, 25-800 °C, 5 °C min⁻¹) of electrodes with and without Li_2_S.

Figure S16 shows the TGA curves of electrodes measured in air with a heating rate of 5 °C min^-1^ up to 800 °C. For the electrode without Li_2_S, a total mass loss of 89.8% was observed above 750 °C, corresponding to a residual fraction of $F_{0}$=0.102. For the electrode containing Li_2_S, the mass loss was 16.0%, leaving a residual fraction of $F_{1}$=0.84. Assuming the initial mass equals 1, the electrode consists of carbon ($c$), inorganic residue Fe_2_O_3_/Fe-N-C ($a$), and Li_2_S ($l$).

During heating, carbon is completely combusted, contributing to a weight loss of $c$. Fe_2_O_3_/Fe-N-C remains stable, leaving a residual fraction of a (determined as 0.102 from the Li_2_S-free electrode). Li_2_S is oxidized to Li_2_SO_4_, leading to a weight increase. The corresponding mass ratio is:

$$r=\frac{M_{Li2SO4}}{M_{Li2S}}=\frac{109.94}{45.95}\approx2.393$$

giving a final residual mass of $r\times l$

From the residual fraction of the Li_2_S-containing electrode:

$$a+r\times l=F_{1}$$

$$l=\frac{F_{1}-a}{r}=\frac{0.84-0.102}{2.393}\approx0.308$$

Thus, the initial Li_2_S mass fraction is ~30.8%. The carbon content is obtained from:

$$c=1-a-l=1-0.102-0.308\approx0.59$$

Therefore, the electrode composition is approximately: Li_2_S ≈ 31%, Fe_2_O_3_/Fe-N-C ≈ 10%, and carbon matrix ≈ 59%.


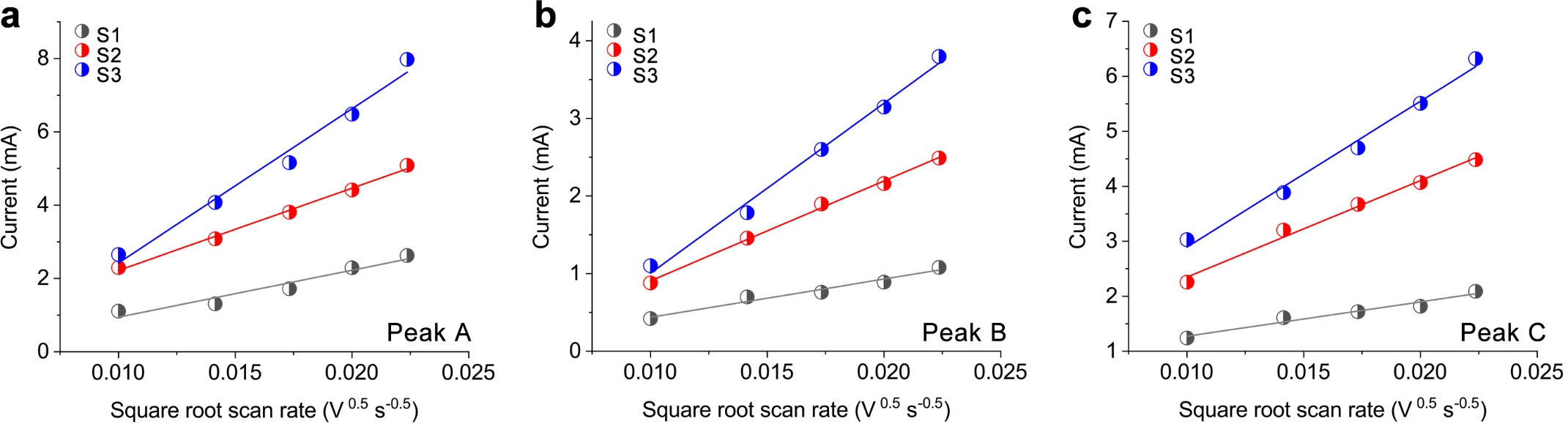


Figure S17. Peak current as a function of the square root of scan rate for the S1, S2, and S3 electrodes at (a) peak A, (b) peak B, and (c) peak C of the CV curves in Figure 3a-c The linear relationship demonstrates the diffusion-controlled nature of the redox processes, and the steeper slopes observed in the S3 electrode indicate enhanced Li^+^ diffusion kinetics compared to S1 and S2.

According to the Randles-Sevcik equation, the diffusion coefficient (D_Li+_) was determined from the slope of the linear fitting between peak current (I) and the square root of the scan rate (v_1/2_). Therefore, the peak currents obtained at different scan rates and their corresponding square roots are plotted. As shown in Figure S17a, the slopes of S1, S2, and S3 electrodes at peak A are 128.1, 224.4, and 420.5, respectively. Similarly, Figure S17b shows slopes at peak B of 62.6 (S1), 175.6 (S2), and 265.2 (S3). Figure S17c displays slopes at peak C, with 49.3 for S1, 128.7 for S2, and 218.9 for S3. These obtained slopes were used to calculate the D_Li+_ values of the S1, S2, and S3 electrodes at peaks A, B, and C. The calculated diffusion coefficients are summarized and plotted in Figure 3d of the main manuscript.


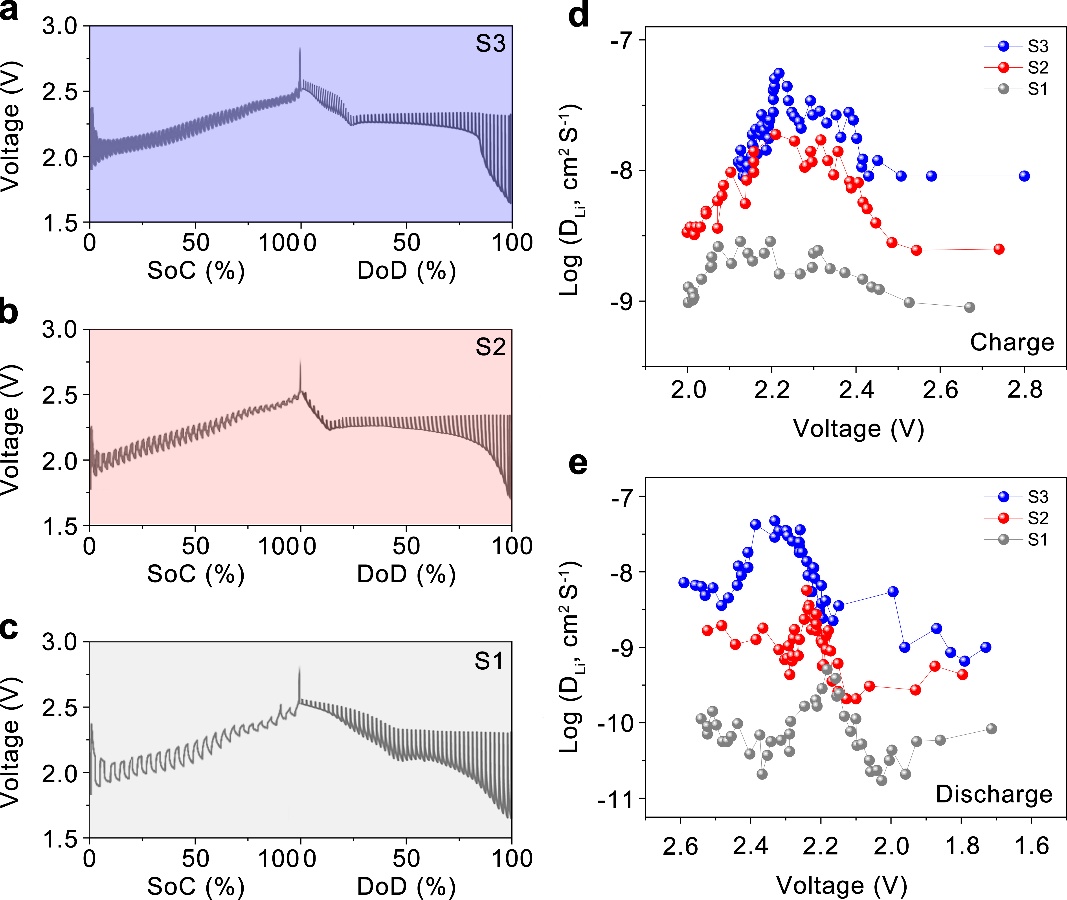


Figure S18. GITT curves of (a) S3 electrode, (b) S2 electrode, and (c) S1 electrode, and the corresponding values of chemical diffusion coefficient (D_Li_) in the (d) charge process and (e) discharge process

The corresponding GITT curves for S3, S2, and S1 electrodes are presented in Figure S18a-c, respectively. All three electrodes exhibit distinct potential plateaus at around 2.1-2.4 V, corresponding to the typical two-step redox reactions of sulfur species. The diffusion coefficient of Li⁺ ($D_{\text{Li}}$) at each titration step was calculated according to Weppner and Huggins’ equation^[14]^:

$$D_{\text{Li}}=\frac{4}{\pi}{(\frac{m_{B}V_{M}}{M_{B}S})}^{2}{(\frac{\Delta E_{s}}{\Delta E_{\tau}})}^{2}\frac{1}{\tau}$$

where $m_{B}$and $M_{B}$are the active material mass and molar mass, $V_{M}$ is the molar volume, $S$ is the electrode area, $\tau$is the duration of the current pulse, $\Delta E_{\tau}$is the change in cell voltage during the constant current pulse (after eliminating the IR drop), and $\Delta E_{s}$is the steady-state voltage change after relaxation.

The calculated $D_{\text{Li}}$ values for the charge and discharge processes are shown in Figure S18d and S18e, respectively. A noticeable increase in $D_{\text{Li}}$ is observed near 2.2 V during charging and around 2.3 V during discharging for all electrodes, which can be attributed to the generation of soluble LiPSs that enhance Li⁺ mobility.

It should be noted that, since Li-S conversion involves multi-phase reactions rather than single-phase diffusion, the GITT analysis here is used qualitatively to reveal relative trends in ion transport kinetics, rather than to determine absolute diffusion coefficients. Nevertheless, the S3 electrode consistently exhibits higher apparent diffusivity than S2 and S1, confirming that the dual-gradient architecture effectively promotes Li⁺ transport and accelerates LiPSs conversion kinetics, which are consistent with the CV-derived diffusion coefficients based on the Randles-Sevcik analysis.


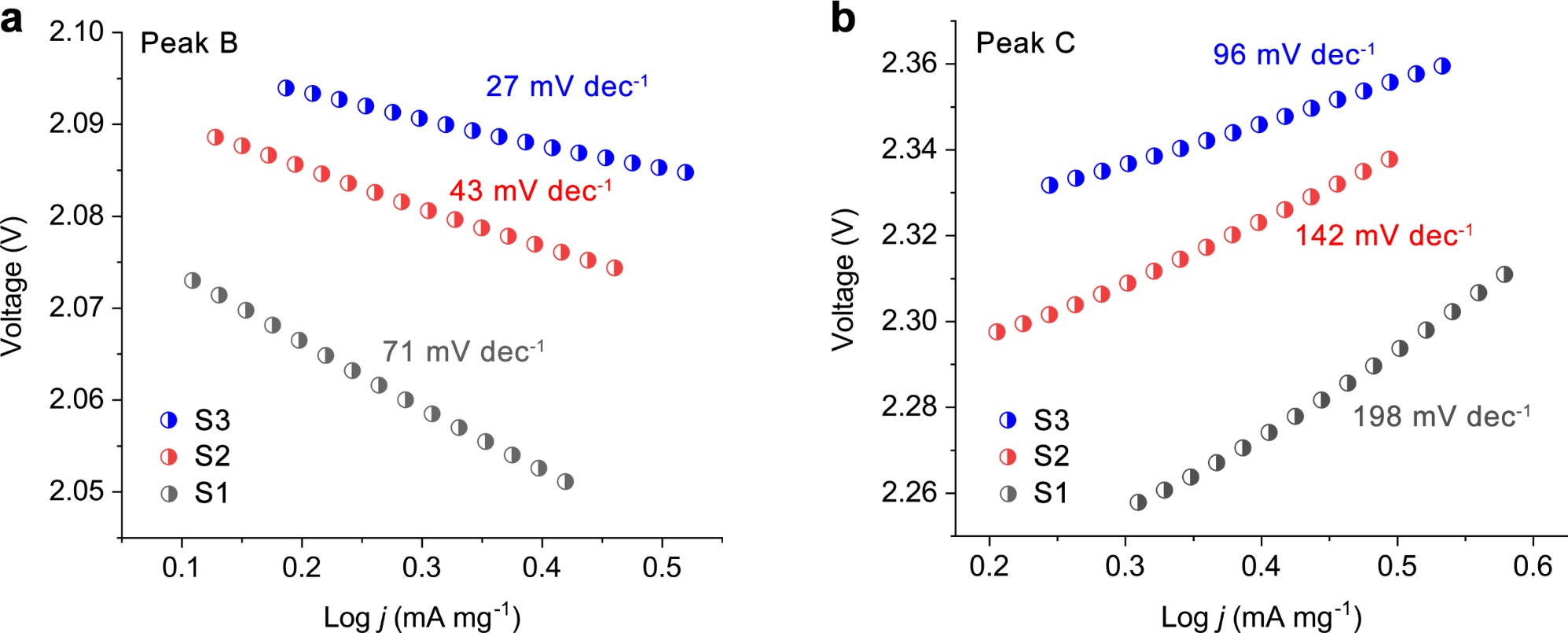


Figure S19. Tafel plots of (a) peak B and (b) peak C for the S1 (gray), S2 (red), and S3 (blue) electrodes. The Tafel slopes derived from these plots reflect the reaction kinetics during the sulfur redox processes.

The slopes of Tafel plots in Figure S19a and S19b at peak B and peak C were calculated to analyze the catalytic activity of the reaction process of short-chain LiPSs (Li_2_S_4_/Li_2_S_2_) reduction and Li_2_S formation, respectively. In comparison with the S1 and S2 electrodes, the S3 electrode exhibited more favorable kinetics for both SER and SRR processes of Li-S batteries simultaneously. This may be attributed to the synergistic effect between the Fe_2_O_3_/Fe-N-C materials and the correlated dual-gradient structural electrode design.


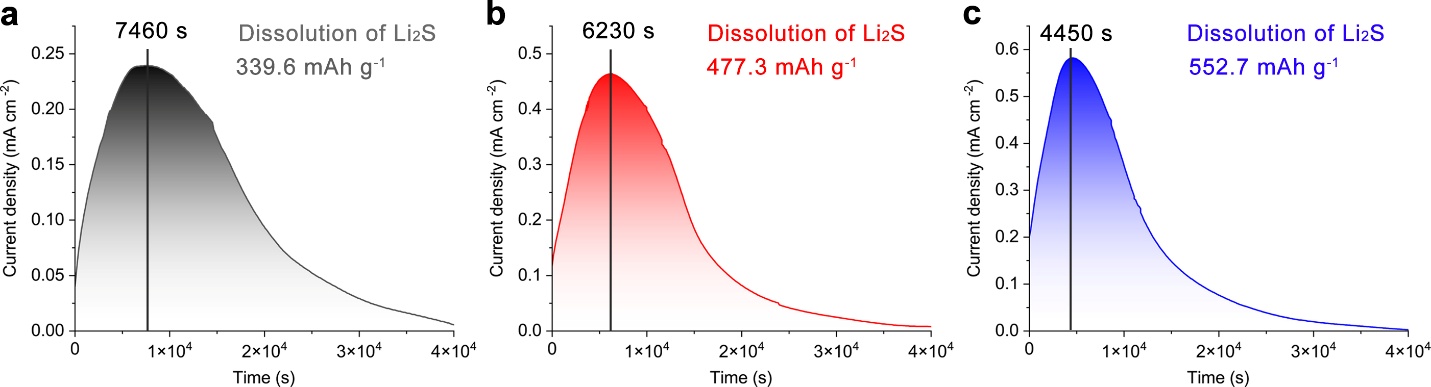


Figure S20. The dissolution profiles of Li_2_S for (a) S1, (b) S2, and (c) S3 electrodes.

The Li_2_S dissolution behavior was evaluated from potentiostatic current-time measurements at 2.4 V to reflect the dissolution kinetics of Li_2_S in the S1, S2, and S3 electrodes. As shown in Figure S20a, the S1 electrode reaches a peak current density of 0.24 mA cm^-2^ at 7,460 s, corresponding to a dissolution capacity of 339.6 mAh g^-1^. As presented in Figure S20b, the S2 electrode shows an enhanced dissolution capacity of 477.3 mAh g^-1^ (higher than that of the S1 electrode, due to the reduced electrode tortuosity facilitating improved Li-ion transport), achieving a higher peak current density of 0.46 mA cm^-2^ (also indicating enhanced kinetics resulting from lower electrode tortuosity) at a shorter response time of 6,230 s. Notably, as illustrated in Figure S20c, the S3 electrode exhibits the highest dissolution capacity of 552.7 mAh g^-1^, surpassing both the S1 and S2 electrodes, along with the highest peak current density of 0.58 mA cm^-2^ and the shortest response time (4,450 s). These results clearly demonstrate that the dual-gradient structural design of the S3 electrode significantly improves Li_2_S dissolution kinetics compared to the S1 and S2 electrodes, resulting in superior electrochemical performance.


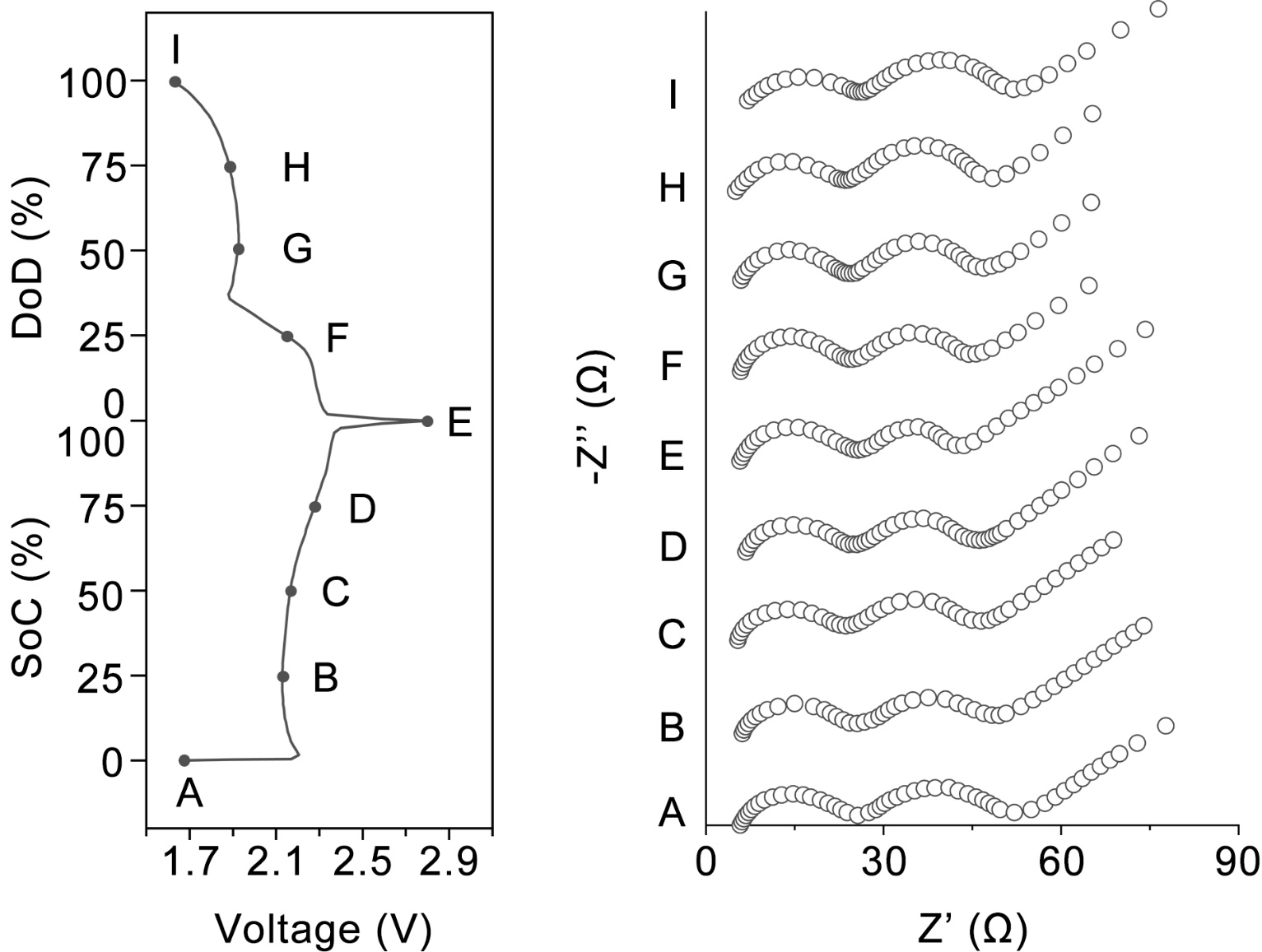


Figure S21. In situ EIS analysis of the S1 electrode and corresponding voltage profile during the second cycle.

The Nyquist plots collected at different states of charge display two distinct semicircles in the high- and medium-frequency regions, followed by a short-inclined tail in the low-frequency region. The intercept at the real axis (Z’) represents the bulk electrolyte resistance. The first semicircle corresponds to the interfacial contact resistance between the electrode components (e.g., carbon, sulfur, binder, and electrolyte), while the second semicircle is associated with the charge transfer resistance at the cathode/electrolyte interface during redox reactions. The equivalent circuit models and the respective resistance components are compared later in Figure S23 and S24.


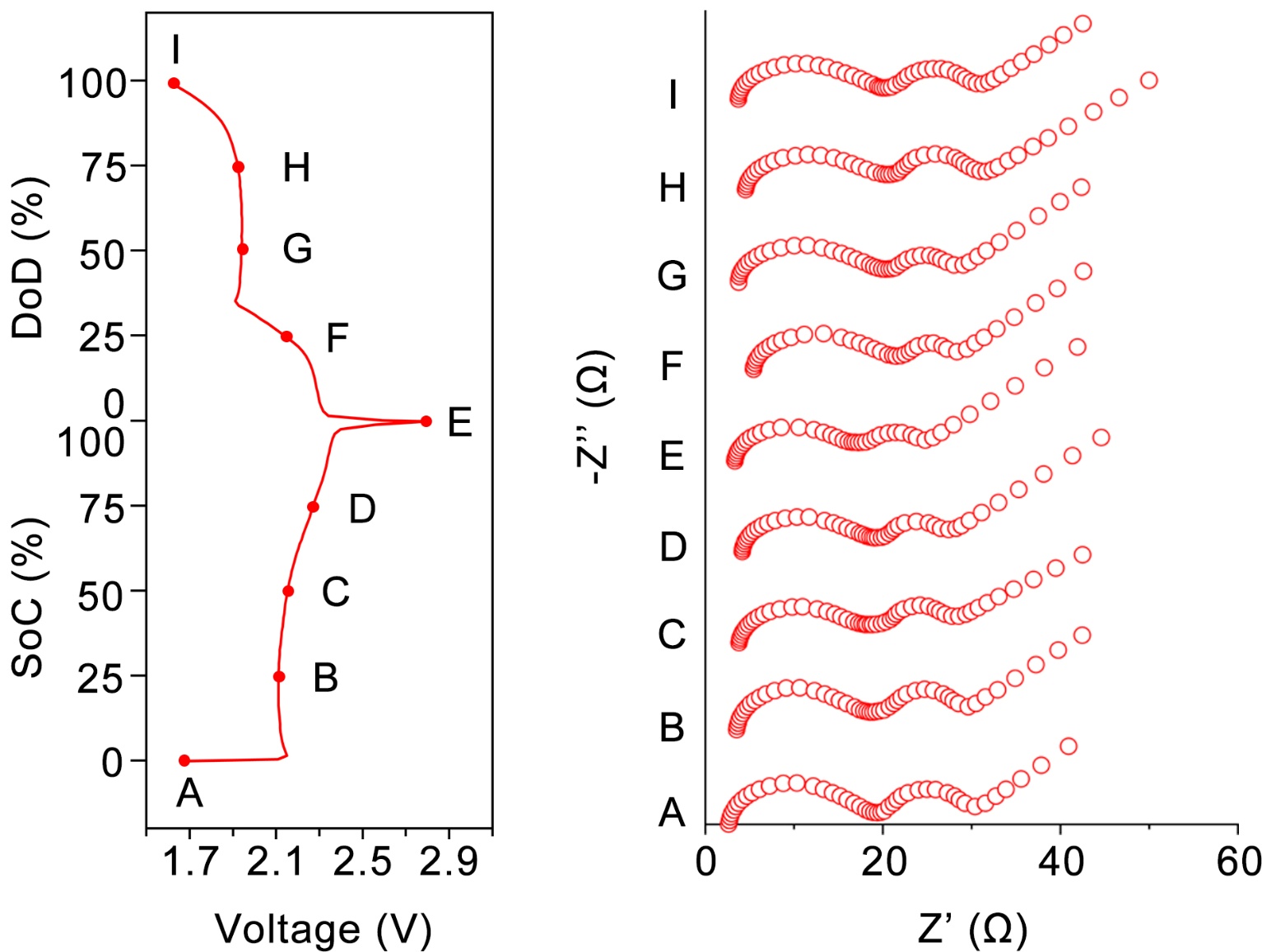


Figure S22. In situ EIS analysis of the S2 electrode and corresponding voltage profile during the second cycle.

Similar to the S1 electrode, the Nyquist plots of the S2 electrode exhibit two semicircles at high and medium frequencies, along with a low-frequency tail. The first intercept at the x-axis reflects the electrolyte resistance. The first semicircle is assigned to interfacial contact resistance between the electrode components (e.g., carbon, sulfur, binder, and electrolyte), and the second semicircle corresponds to charge transfer resistance between the cathode and the electrolyte. These measurements provide insight into the evolution of electrochemical impedance during the cycling process. The equivalent circuit models and the respective resistance components are compared later in Figure S23 and S24.


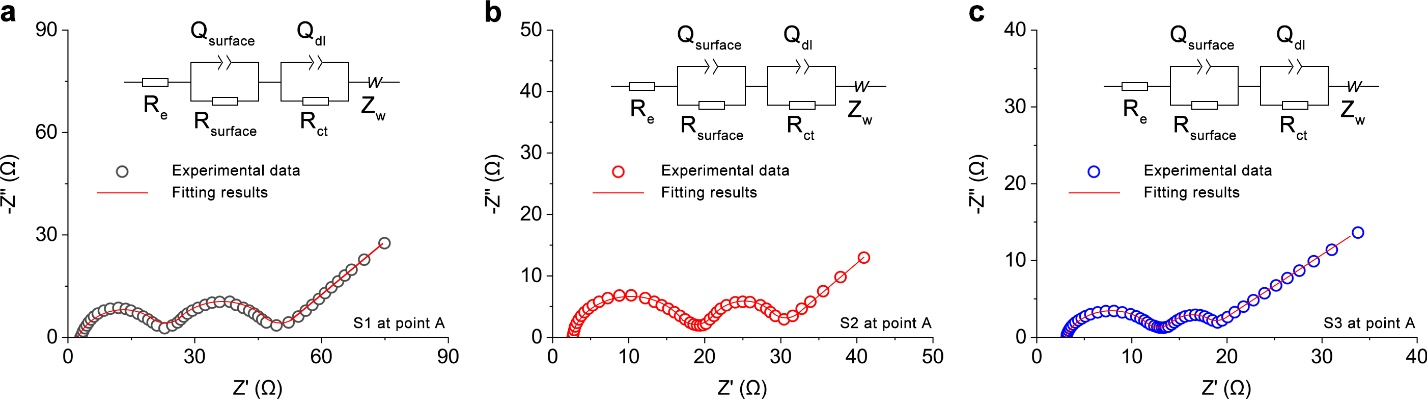


Figure S23. Equivalent circuit fitting of Nyquist plots for (a) S1, (b) S2, and (c) S3 electrodes collected at the same electrochemical state (point A in the voltage profile associated with each Nyquist plot).

The Nyquist plots of the three electrodes, shown in Figure 3h in the main text and Figure S21 and S22, were analyzed using equivalent circuit models to extract detailed resistance and capacitance parameters. Each model comprises a series of electrolyte resistance (R_e_), an interfacial resistance (R_surface_) in parallel with a constant phase capacitor (Q_surface_) to fit the high-frequency semicircle, and a charge transfer resistance (R_ct_) in parallel with another constant phase capacitor (Q_ct_) to fit the medium-frequency semicircle. A Warburg element (Z_w_) is also incorporated to account for the Li^+^ diffusion behavior. All remaining Nyquist plots (from point B to I) were fitted using the same equivalent circuit model for consistency.


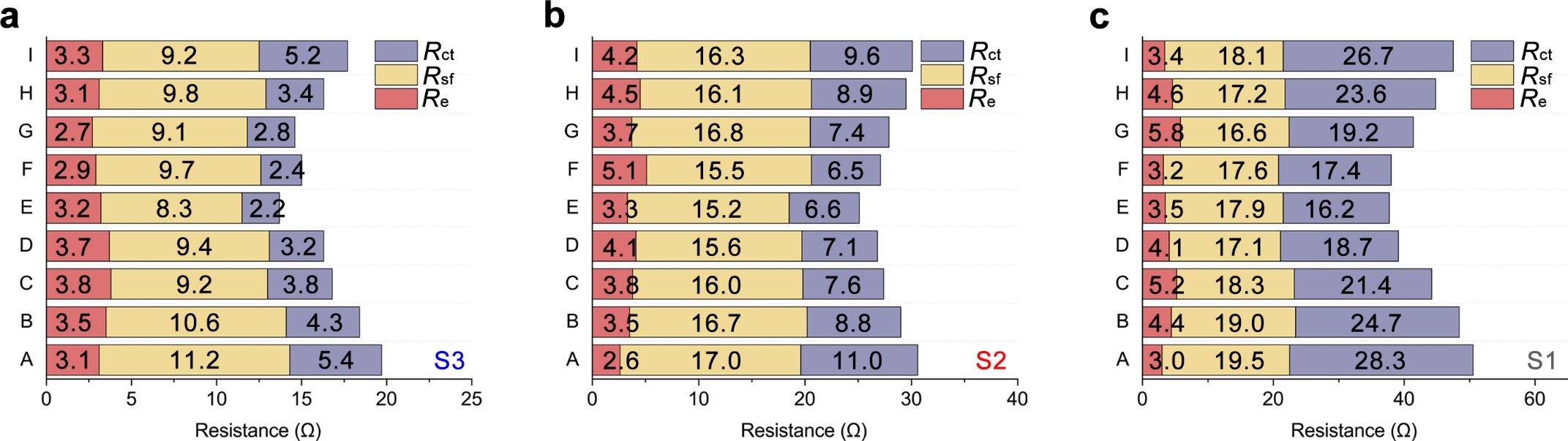


Figure S24. The statistical bar graph of R_e_, R_ct_, and R_sf_ of (a) S3, (b) S2, and (c) S1 electrodes during the second cycle.

Each stacked bar represents the total resistance, comprising electrolyte resistance (R_e_), interfacial contact resistance (R_surface_), and charge transfer resistance (R_ct_). As shown in Figure S24, R_e_ remains relatively constant (~5 Ω) across all samples, indicating stable bulk ionic conductivity. The S3 electrode consistently shows the lowest R_s_ and R_ct_ values across the full cycle, suggesting minimized SEI formation and enhanced Li^+^ transport and charge transfer kinetics as indicated in Figure S24a. The S2 electrode exhibits moderate resistance values as shown in Figure S24b, while the S1 electrode suffers from the highest R_s_ and R_ct_, particularly toward 0% SoC (point A) and 100% DoD (point I), corresponding to sluggish kinetics during Li_2_S_2_/Li_2_S formation and decomposition (Figure S24c). These observations quantitatively confirm that the dual-gradient structure in the S3 electrode not only suppresses interfacial resistance but also accelerates sulfur redox reactions, resulting in improved electrochemical reversibility and lower polarization.


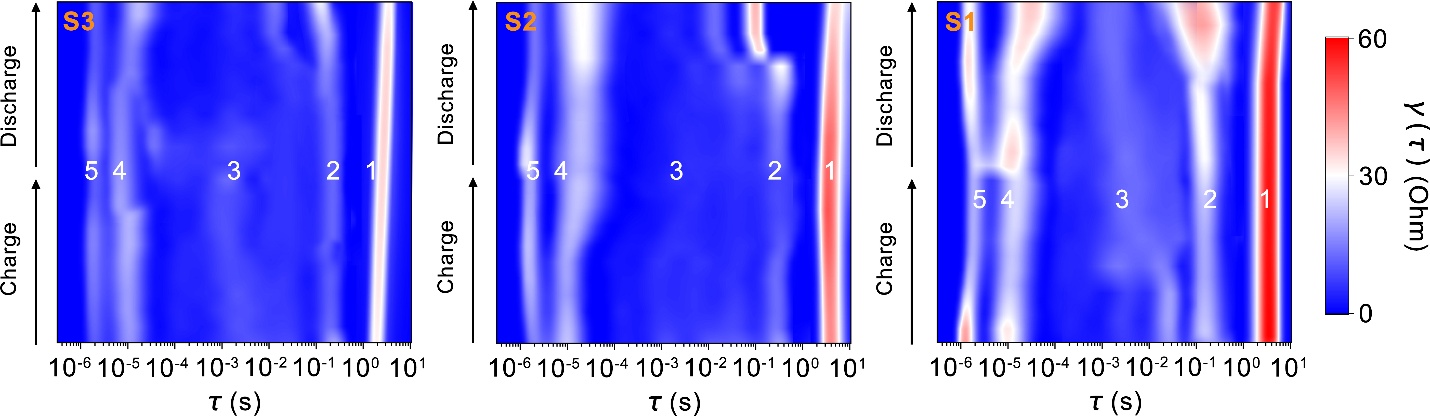


Figure S25. DRT plots (range from 10-6 to 101 s) of the S3, S2, and S1 electrodes at different charge-discharge states.

To further clarify the impedance evolution revealed by the Nyquist plots, the distribution of relaxation times (DRT) analysis was performed for S1, S2, and S3 electrodes during cycling. The DRT spectra resolve five characteristic processes of Li-S batteries, as labeled in Figure S25.

For the S3 electrode, all peaks are narrower and weaker than those of S2 and S1, demonstrating the most homogeneous ion/electron transport. The weakened peak 5 at the end of charge/discharge confirms reduced electrolyte polarization, and the leftward shift of peak 4 near the end of charge indicates accelerated interfacial relaxation as sulfur oxidation completes. Conversely, peak 4 slightly right-shifts at deep discharge, reflecting slower relaxation due to Li₂S accumulation. The weak peak 3 signifies minimal charge-transfer resistance, while the leftward shift of peak 2 near discharge completion reveals enhanced Li⁺ and polysulfide diffusion. The narrow peak 1 further suggests uniform electronic conduction and low inter-particle resistance.

In the S2 electrode, peak 5 slightly broadens during charge and narrows later while shifting rightward, indicating moderately hindered electrolyte diffusion. Peak 4 broadens during discharge, implying nonuniform double-layer relaxation. Peak 3 remains weak, whereas peak 2 shifts leftward with increased intensity at late discharge, suggesting partial concentration polarization. The relatively stronger peak 1 denotes higher inter-particle resistance than S3.

The S1 electrode exhibits broad and intense peaks across all τ ranges. Strong peak 5 responses at the beginning of charge and end of discharge reveal pronounced electrolyte polarization. Peak 4 becomes broader and shifts rightward, reflecting sluggish interfacial relaxation, while peak 3 shows high intensity, evidencing large charge-transfer resistance. The left-shifted and intensified peak 2 indicates severe polysulfide accumulation and nonuniform diffusion, and the broad, strong peak 1 confirms poor electronic connectivity and high local overpotential.

Collectively, the DRT results verify that the S3 electrode possesses the smallest relaxation times, the most balanced kinetic distribution, and the lowest overall impedance, which is fully consistent with the EIS analysis, highlighting the effectiveness of dual-gradient design in synchronizing redox reactions and promoting fast, homogeneous transport across the electrode thickness.


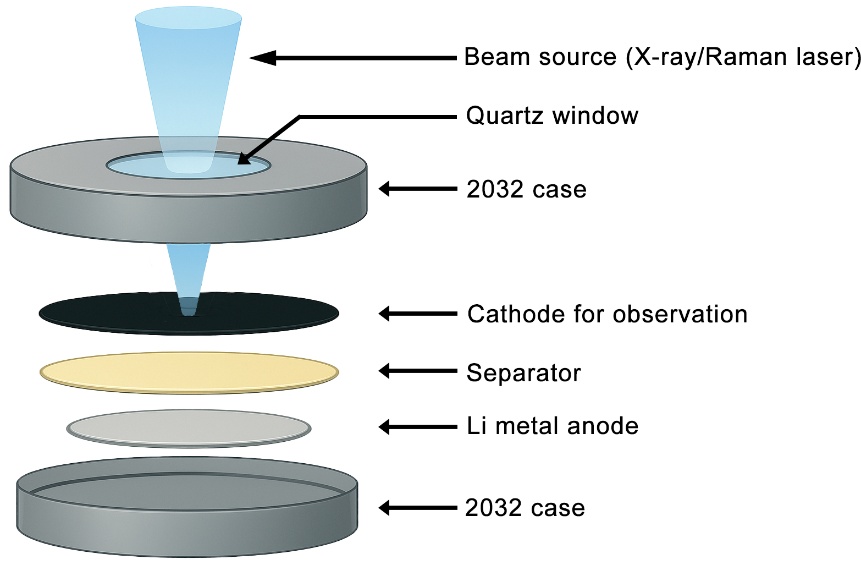


Figure S26 Schematic of the structure of the custom-designed cell for in-situ XRD and Raman observations.

The cell features a quartz window integrated into the top 2032 case, allowing the incident beam from the X-ray or Raman laser to pass through for real-time observation of the cathode. The internal configuration includes the cathode under investigation, a separator, and a lithium metal anode, all assembled within standard 2032 coin cell components.


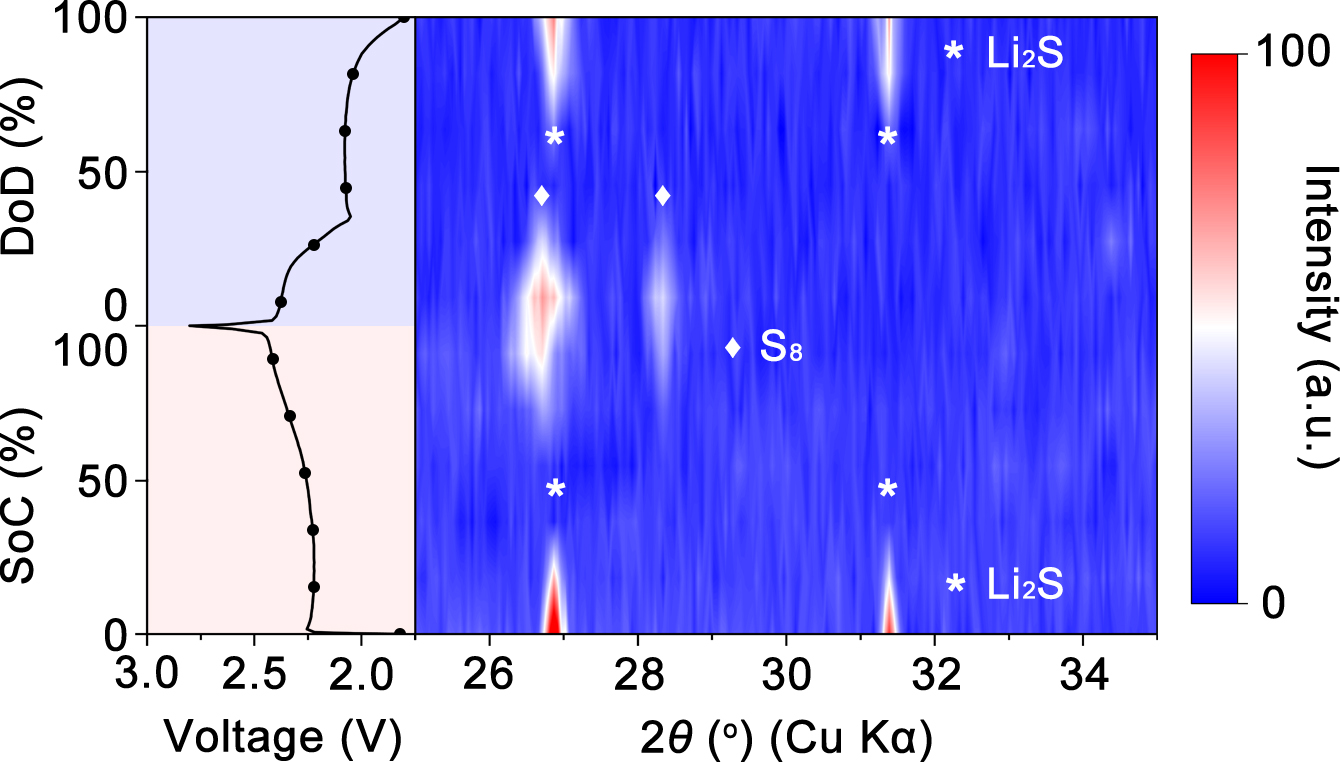


Figure S27. In situ XRD patterns of the S2 electrode collected during the charge-discharge process.

The left panel displays the corresponding voltage profile as a function of SoC and DoD, while the right panel shows the evolution of diffraction peaks with 2θ (Cu Kα). The peaks at ~26.9° and ~31.3° correspond to the characteristic (111) and (200) planes of Li2S, which diminish upon charging, indicating progressive Li_2_S decomposition. Simultaneously, peaks at ~26.7° and ~28.3°, attributed to the (311) and (008) planes of orthorhombic S_8_, gradually emerge, reflecting the formation of solid S_8_ from soluble LiPSs. During discharge, these S_8_ peaks diminish early in the process, reflecting the solid-to-liquid transition as S_8_ is reduced to soluble LiPSs. Toward the end of the discharge, the Li_2_S peaks reemerge, indicating the reformation of Li_2_S via liquid-to-solid conversion reactions.


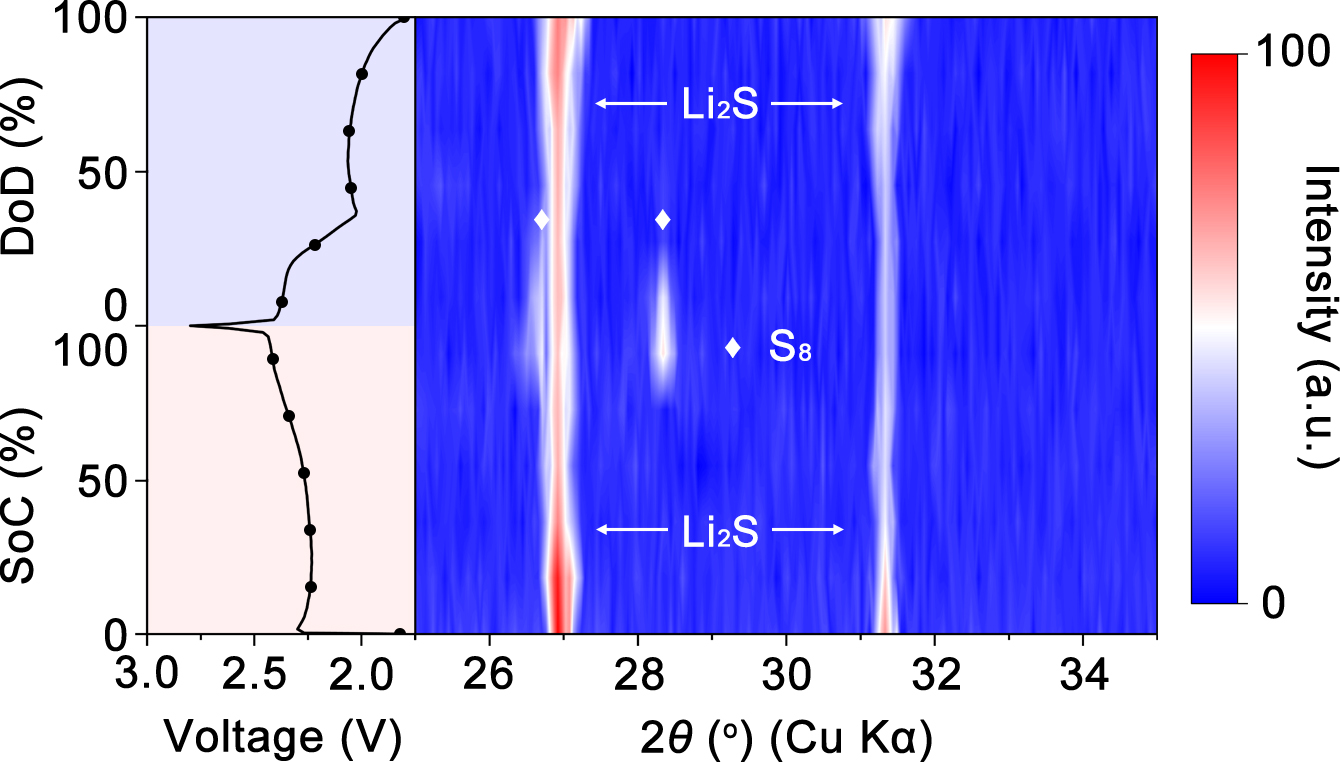


Figure S28. In situ XRD patterns of the S1 electrode collected during the charge-discharge process.

The left panel displays the voltage profile as a function of SoC and DoD, while the right panel presents the corresponding 2D XRD contour map, showing the evolution of diffraction peaks with respect to 2θ (Cu Kα). At the beginning of the charging process, two distinct diffraction peaks remain visible at approximately 26.9° and 31.3° during the entire charge-discharge process, corresponding to the (111) and (200) planes of crystalline Li_2_S, confirming that Li_2_S is the dominant solid phase at the onset of charging. As the charging progresses, these peaks gradually weaken, indicating the stepwise decomposition of Li_2_S. However, unlike the S2 and S3 electrodes, the Li_2_S peak intensity in the S1 electrode remains nearly unchanged after approximately 54% state of charge, suggesting that a significant portion of Li_2_S is not fully consumed by the end of the charging process. Toward the end of charging, diffraction peaks associated with S_8_ begin to emerge, particularly around 26.7° and 28.3°, which correspond to the (311) and (008) planes of S_8_, respectively. Nevertheless, the intensity of the S_8_ peaks is substantially lower than that observed in the S2 and S3 electrodes, indicating limited sulfur regeneration. During the subsequent discharge, the S_8_ peaks progressively diminish, reflecting the reduction of solid sulfur into soluble LiPSs. By the end of discharge, the Li_2_S peaks reappear with increased intensity, indicating the regeneration of Li_2_S as the final discharge product.


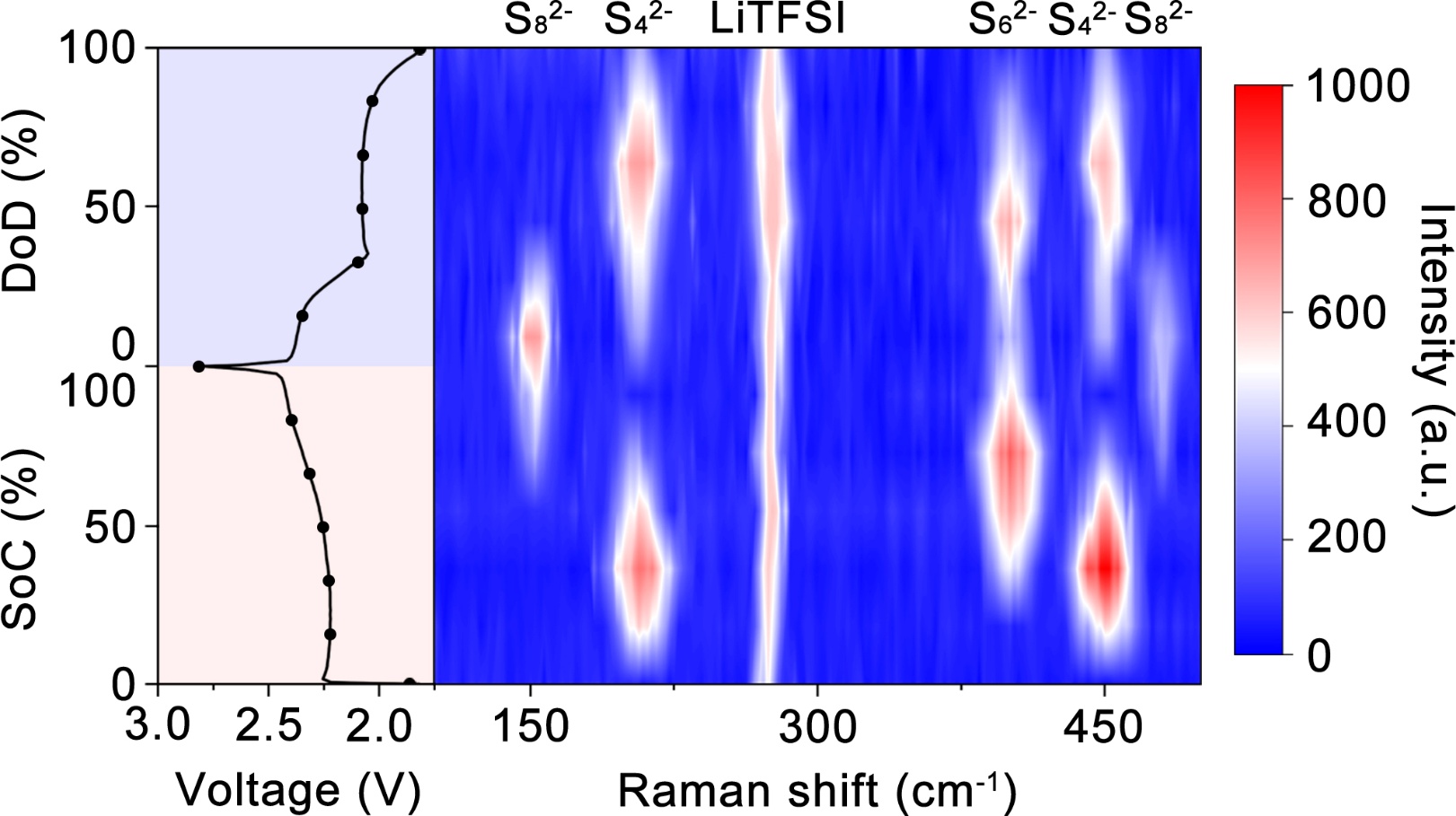


Figure S29. In-situ Raman Spectra of the S2 electrode collected during the charge-discharge process.

The left panel shows the voltage profile as a function of SoC and DoD, while the right panel presents the corresponding in-situ Raman contour plot. The persistent LiTFSI peak at ~282 cm^-1^ serves as a reference and confirms the electrolyte stability during the measurement. Several characteristic peaks are identified and tracked throughout the redox cycle to reveal the dynamic evolution of soluble LiPSs. At the beginning of the charge process, two peaks emerge near 200 cm^-1^ and 450 cm^-1^, corresponding to the symmetric and asymmetric stretching vibrations of Li_2_S_4_, respectively, indicating the initial solid-to-liquid conversion from Li_2_S to Li_2_S_4_. As charging continues, a distinct peak appears at approximately 400 cm^-1^, attributed to Li_2_S_6_, signifying further oxidation of intermediate species. The formation of Li_2_S_8_ is evidenced by the appearance of peaks near 150 cm^-1^ and 473 cm^-1^ (weak) at the same time. However, the persistent presence of the peak at approximately 400 cm⁻¹ at the end of the charging process indicates that Li_2_S_6_ is not fully consumed, suggesting incomplete conversion of intermediate polysulfides. These observations confirm the sequential conversion of Li_2_S to medium- and long-chain LiPSs, including Li_2_S_4_, Li_2_S_6_, and Li_2_S_8_. During the discharge process, the reduction pathway proceeds in reverse, beginning with the disappearance of Li_2_S_8_ and followed by the formation of intermediate species such as Li_2_S_6_ and Li_2_S_4_. The peak near 400 cm⁻¹, attributed to Li_2_S_6_, intensifies in the early stages of discharge, while peaks at 200 and 450 cm^-1^, corresponding to Li_2_S_4_, re-emerge as discharge progresses. Notably, the Li_2_S_4_ peaks remain detectable even at the end of discharge, indicating incomplete conversion of soluble intermediates. This residual signal suggests that the reduction of LiPSs to solid Li_2_S is kinetically sluggish in the S2 electrode, leading to the accumulation of intermediate species and limited Li_2_S regeneration. Overall, the in-situ Raman analysis highlights the prolonged presence and sluggish conversion kinetics of soluble LiPSs in the S2 electrode during both SER and SRR processes.


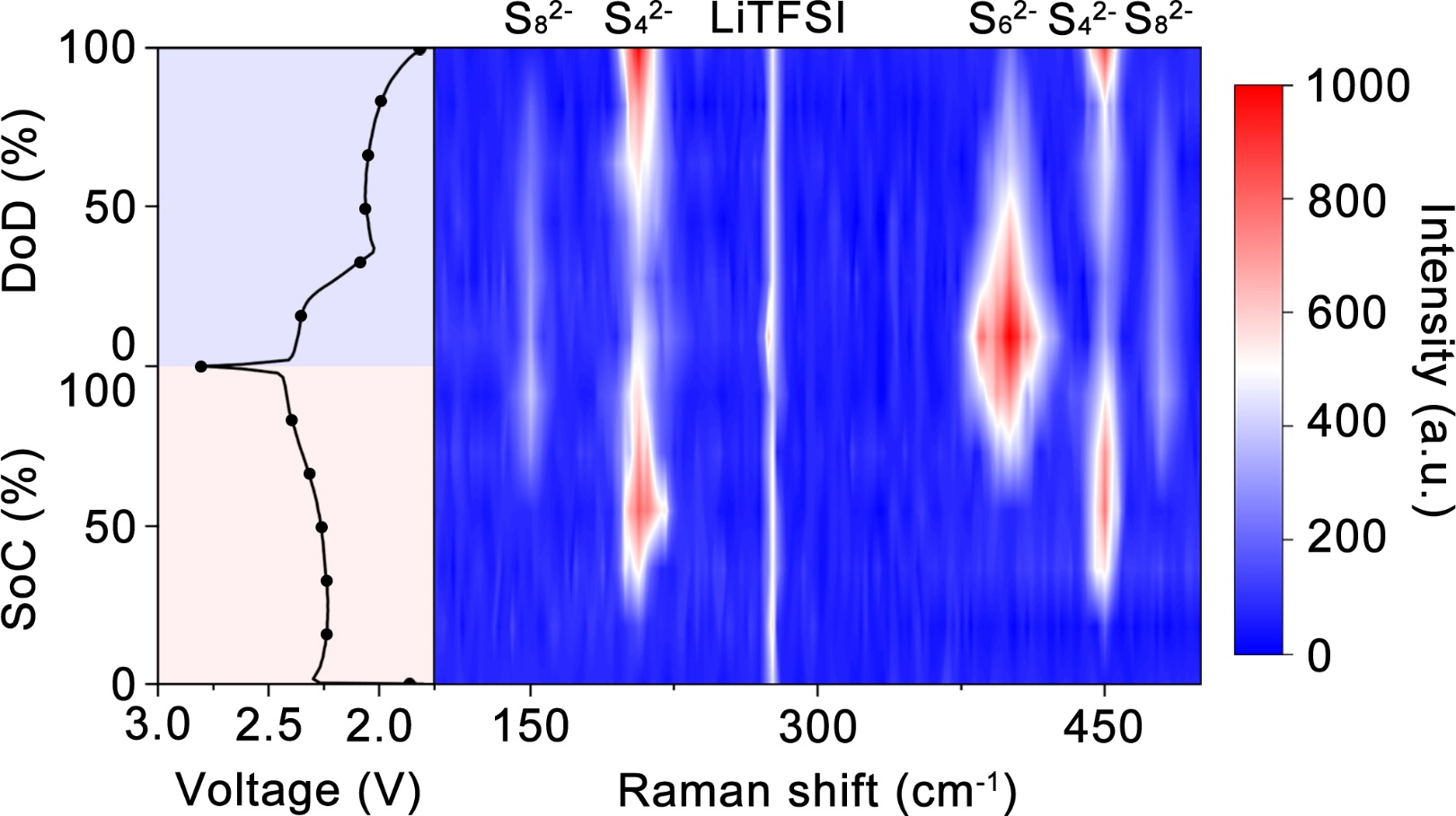


Figure S30. In-situ Raman Spectra of the S1 electrode collected during the charge-discharge process.

The left panel shows the corresponding voltage profile as a function of SoC and DoD, while the right panel presents the evolution of Raman-active species. At about 20% SoC, distinct peaks emerge at ~200 cm^-1^ and ~450 cm^-1^, attributed to Li_2_S_4_, indicating the conversion of solid Li_2_S into soluble LiPSs intermediates. As charging progresses, a peak at ~400 cm^-1^ appears, corresponding to the formation of Li_2_S_6_. In the later stages of charging, weak peaks near ~150 cm^-1^ and ~473 cm^-1^ emerge, representing the generation of long-chain polysulfide Li_2_S_8_. Notably, the Li_2_S_6_ signal remains prominent even at full charge, indicating incomplete consumption of this intermediate during the SER. This incomplete conversion limits the formation of Li_2_S_8_, as reflected by its faint peaks in the high-frequency region. Consequently, the amount of solid S8 generated is also reduced, which may restrict the achievable charge capacity. During the subsequent discharge, the Li_2_S_8_ signal, which is already weak due to its limited formation, disappears rapidly. The peak at ~400 cm^-1^, corresponding to Li_2_S_6_, gradually diminishes toward the end of the discharge, suggesting the progressive consumption of Li_2_S_6_ accumulated during the charge process. Meanwhile, peaks at ~200 cm⁻¹ and ~450 cm⁻¹, attributed to Li_2_S_4_, increase in intensity as Li_2_S_6_ is reduced. However, because a considerable amount of Li_2_S_6_ persists until late in the discharge, Li_2_S_4_ remains detectable at the end, implying incomplete reduction of soluble intermediates to Li_2_S. These results collectively indicate sluggish LiPSs conversion kinetics in the S1 electrode during both the SER and SRR processes, with significant retention of soluble species throughout the electrochemical cycle.


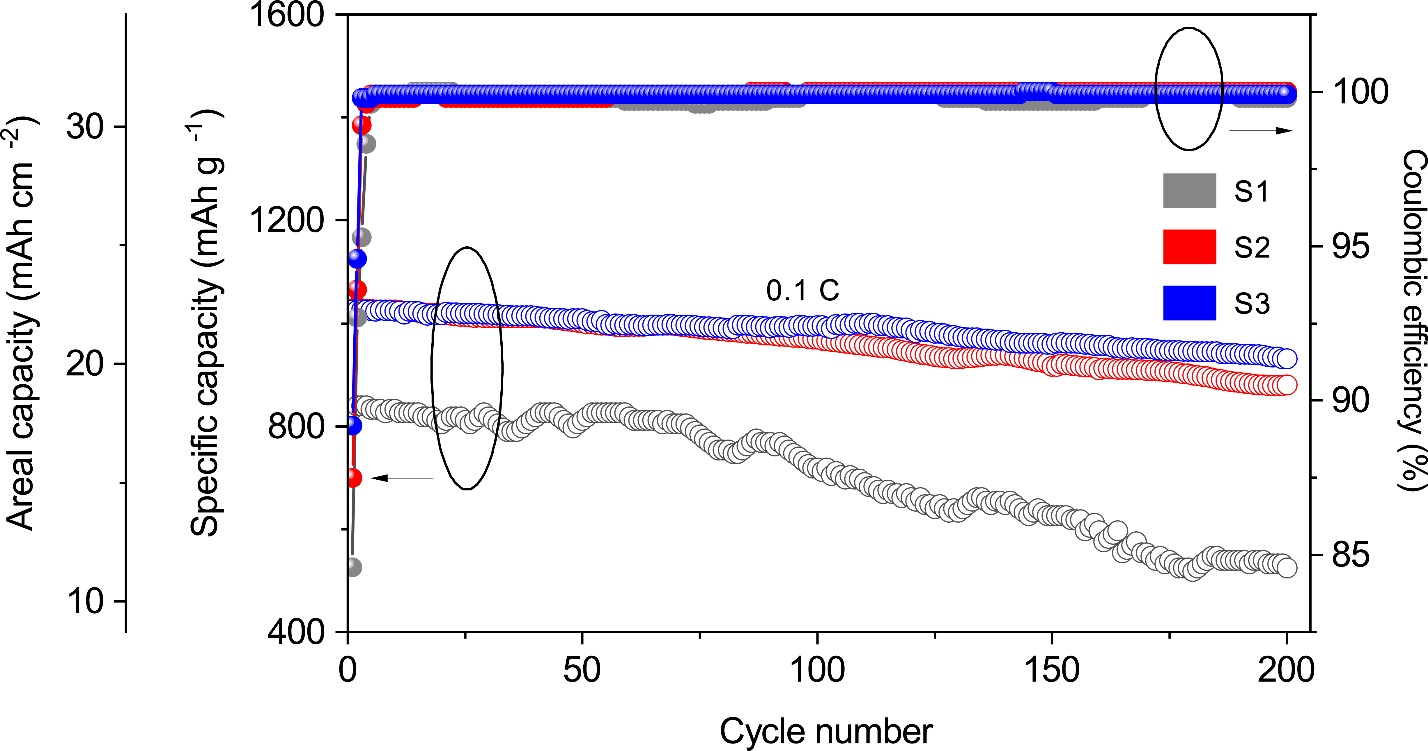


Figure S31. Cycling performance of the S1, S2, and S3 electrodes under 0.1 C

The long-term cycling performance of the S1, S2, and S3 electrodes was evaluated at 0.1 C for 200 cycles, as shown in Figure S31. The S3 electrode delivers the highest initial specific capacity of approximately 1,048 mAh g^-1^ (22.7 mAh cm^-2^), over five times greater than that of traditional LIB cathodes and retains over 941 mAh g^-1^ (20.3 mAh cm^-2^) after 200 cycles with minimal capacity fading. Its high average Coulombic efficiency of 99.92% reflects excellent reversibility and cycling stability, attributed to the dual-gradient architecture that ensures uniform reaction kinetics and suppresses the accumulation of soluble polysulfide intermediates. The S2 electrode shows a moderate initial capacity of around 1,041 mAh g^-1^ (22.5 mAh cm^-2^), stabilizing at 890 mAh g^-1^ (19.2 mAh cm^-2^) after 200 cycles. Its sustained Coulombic efficiency (~99.83%) suggests improved reversibility over S1, likely due to reduced tortuosity and enhanced Li^+^ transport enabled by the 3D uniform framework. In contrast, the S1 electrode suffers from pronounced capacity decay, dropping from an initial 833 mAh g^-1^ (18 mAh cm^-2^) to below 525 mAh g⁻¹ (11.3 mAh cm^-2^), with an average Coulombic efficiency of 99.53%. This degradation arises from sluggish redox kinetics and the persistent buildup of unconverted polysulfides, leading to poor reversibility and material utilization. Overall, the results clearly show that the rational structure design, particularly the dual-gradient framework in the S3 electrode, substantially enhances long-term cycling stability, active material utilization, and reversibility in thick Li-S electrodes.


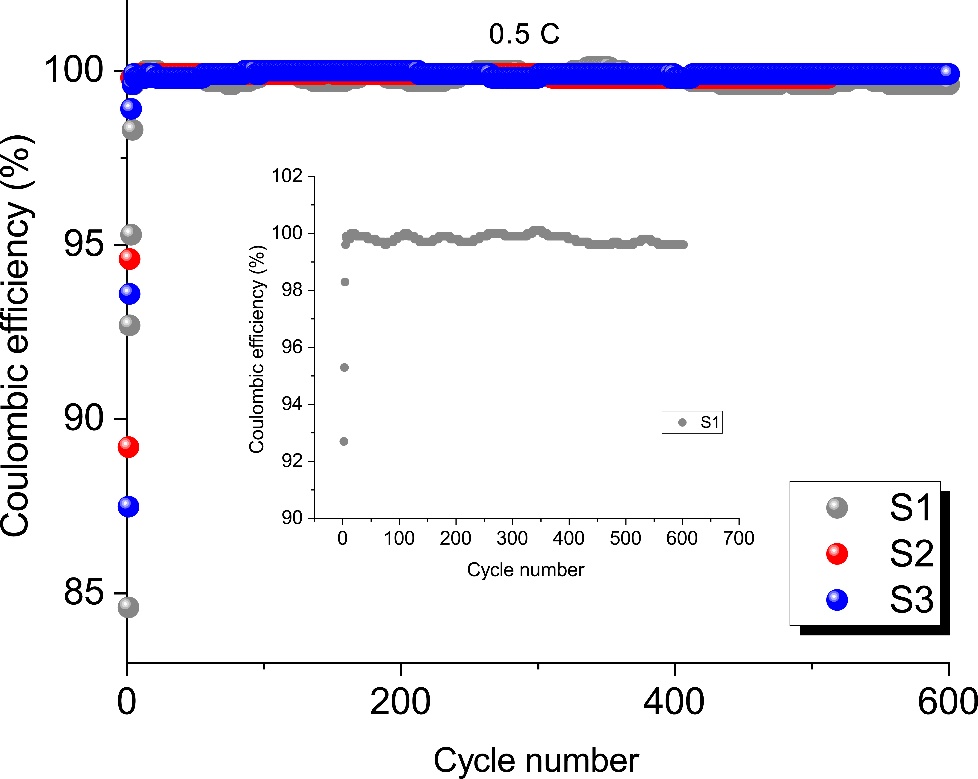


Figure S32. Coulombic efficiency of S1, S2, and S3 electrodes under 0.5 C (Inset, Coulombic efficiency of S1 electrode).

All three electrodes exhibit high CEs after the initial cycles, indicating good reversibility of the redox reactions. Notably, the S3 electrode maintains an average CE of 99.89% throughout the 600-cycle test, reflecting superior stability. The S2 electrode also shows improved stability with an average CE of 99.68%, though slightly lower than that of S3. In contrast, the S1 electrode presents more fluctuations CE during the early cycles, suggesting less efficient polysulfide conversion and higher side reactions. These trends confirm that the structural design of the S3 electrode effectively enhances long-term electrochemical stability under practical cycling conditions.


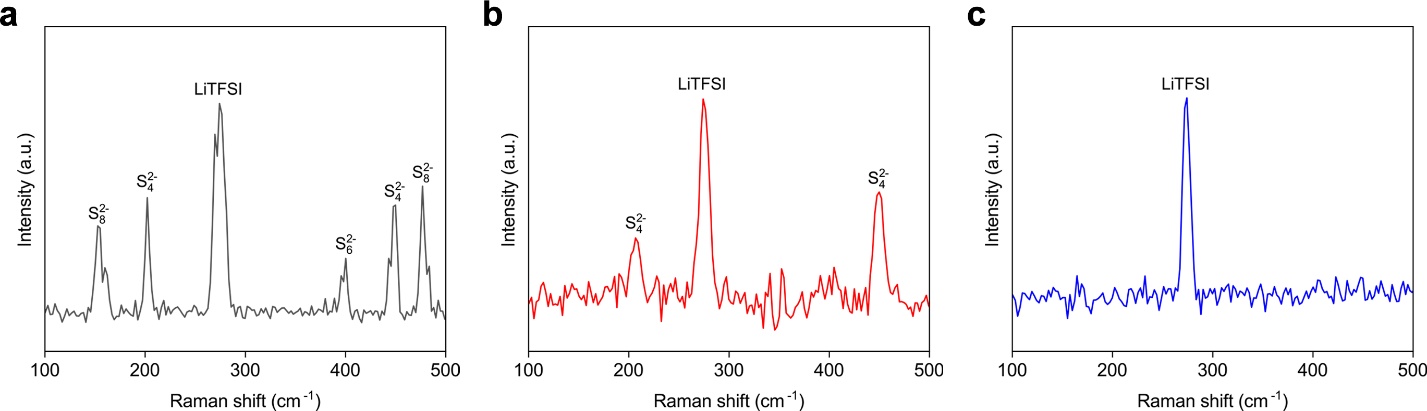


Figure S33. Raman spectra of (a) S1 electrode after 200 cycles, (b) S2 and (c) S3 electrodes after 1100 cycles

Distinct Raman peaks corresponding to polysulfide species (Li_2_S_4_ at ~200 cm^-1^ and ~450 cm^-1^, Li_2_S_6_ at ~400 cm^-1^, and Li_2_S_8_ at ~150 cm^-1^) are clearly observed in the S1 electrode, indicating significant accumulation of residual soluble polysulfides after long-term cycling. This unconsumed fraction, resulting from sluggish conversion kinetics, can be attributed to high tortuosity. The S2 electrode exhibits residual Li_2_S_4_ signals, consistent with its accumulation at the end of each discharge cycle. In contrast, the S3 electrode displays negligible polysulfide-related signals, with only the LiTFSI peak remaining, indicating efficient consumption of soluble LiPSs enabled by enhanced conversion kinetics.


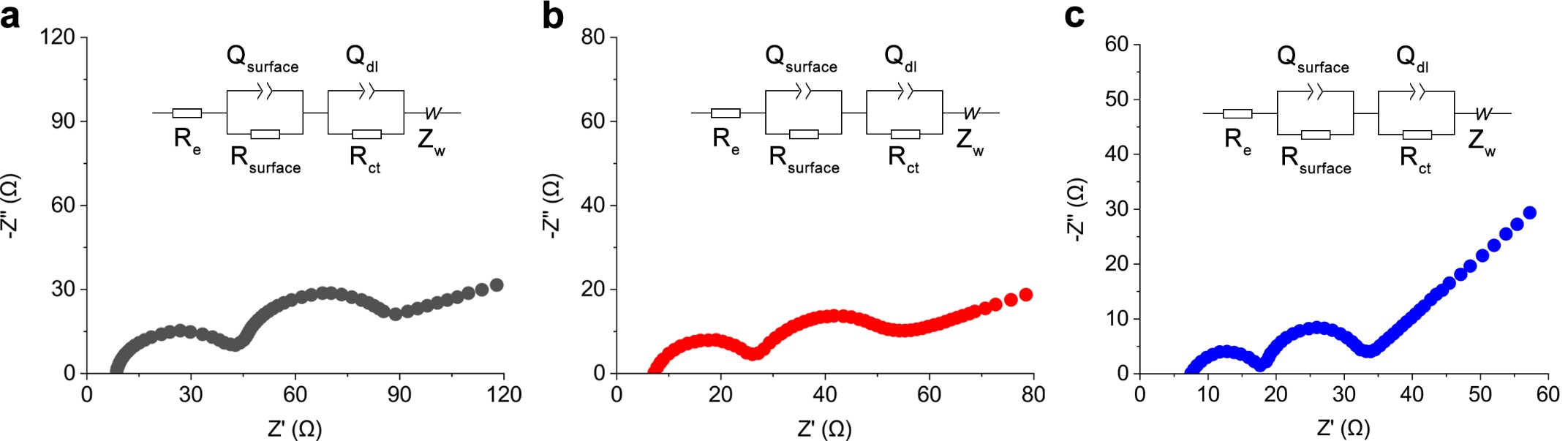


Figure S34. EIS results of (a) S1 electrode after 200 cycles, (b) S2 and (c) S3 electrodes after 1100 cycles

The Re values of ~ 8.6 ohm (S1), ~7.7 ohm (S2), and ~7.8 ohm (S3)) show minimal variation among the S1, S2, and S3 electrodes, indicating comparable bulk ionic conductivity. The S1 electrode exhibits the highest Rs of 37.6 ohm, while the S2 electrode presents a slightly lower Rs of 19.4 ohm, which remains higher than that of the S3 electrode (9.3 ohm), suggesting that S3 possesses the most efficient electrode/electrolyte interfacial contact. Regarding R_ct_, S1 also shows the largest value (49.3 ohm), exceeding that of S2 (33.1 ohm) and S3 (15.2 ohm), indicating progressively improved charge-transfer kinetics from S1 to S3. The lower R_ct_ of S3 compared to S2 can be attributed to the optimized electrode architecture and enhanced catalytic activity of the Fe_2_O_3_/Fe-N-C framework, which promotes more favorable redox reaction pathways.


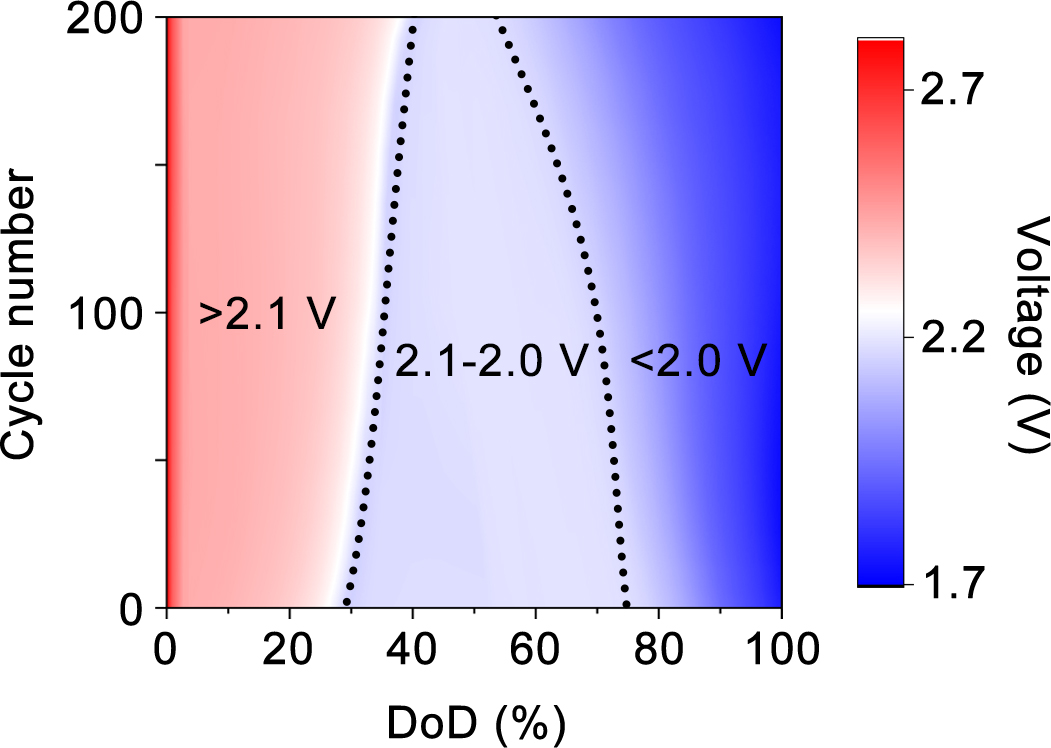


Figure S35. 2D contour plot of the discharge voltage as a function of DoD and cycle number of the S1 electrode

Two-dimensional contour plot showing the discharge voltage distribution of the cell as a function of depth of discharge (DoD) and cycle number. It can be observed that the length of the voltage plateau (2.1-2.0) shrank rapidly during cycling, indicating a progressive increase in polarization and a decline in sulfur utilization. This behavior can be attributed to the gradual accumulation of insulating discharge products and soluble polysulfides, which hinder Li^+^ transport and slow the redox conversion kinetics over prolonged operation.

| Number in Figure 5f | Journal, Year and Ref. | Areal capacity (mAh cm^-2^) | Highest C-rate tested | CUR at the highest rate (%) | Capacity degradation per cycle (%) |
| --- | --- | --- | --- | --- | --- |
| 1 | JACS, 2023 ^[15]^ | 6.2 | 7 | 0.453431 | 0.015 |
| 2 | Adv. Mater., 2023 ^[16]^ | 4.2 | 5 | 0.197304 | 0.075 |
| 3 | Nature Comm., 2023 ^[17]^ | 8.77 | 4.8 | 0.322304 | 0.058 |
| 4 | Adv. Mater., 2024 ^[18]^ | 7.2 | 5 | 0.36152 | 0.018 |
| 5 | Energy Enviro. Sci., 2023 ^[19]^ | 6.4 | 5 | 0.590106 | 0.058 |
| 6 | Nature Comm., 2024 ^[20]^ | 7.5 | 5 | 0.423897 | 0.027 |
| 7 | Nature Comm., 2024 ^[21]^ | 6 | 5 | 0.457721 | 0.059 |
| 8 | Energy Enviro. Sci., 2024 ^[22]^ | 6.5 | 5 | 0.612745 | 0.06 |
| 9 | Adv. Funct. Mater., 2025 ^[23]^ | 4.93 | 4 | 0.481449 | 0.0651 |
| 10 | Adv. Energy Mater., 2024 ^[24]^ | 7.5 | 3 | 0.324142 | 0.077 |
| 11 | Adv. Energy Mater., 2025 ^[25]^ | 8 | 3 | 0.40625 | 0.198 |
| 12 | Adv. Energy Mater., 2025 ^[26]^ | 6 | 3 | 0.544118 | 0.058 |
| 13 | Adv. Mater., 2024 ^[27]^ | 8.2 | 2 | 0.442402 | 0.038 |
| 14 | Adv. Energy Mater., 2025 ^[28]^ | 3.3 | 2 | 0.447304 | 0.052 |
| 15 | Adv. Energy Mater., 2025 ^[29]^ | 7.4 | 2 | 0.551471 | 0.041 |
|  | This work | 22.2 | 5 | 0.641343 | 0.016 |

Table S3. Comparison of electrochemical performance parameters corresponding to Figure 5f, including areal capacity, highest rate tested, CUR at the highest rate, and capacity degradation per cycle. Literature data are listed for reference, and the results for “This work” are obtained from the present study.

Table S3 presents a comparison of electrochemical performance parameters reported in Figure 5f, including areal capacity, highest rate tested, CUR at the highest rate, and capacity degradation per cycle. In this work, the CUR was calculated as the ratio of the experimentally released discharge capacity to the theoretical capacity of Li_2_S, i.e.:

$$\text{CUR}=\frac{Q_{\text{discharge}}}{Q_{\text{theoretical}}}=\frac{Q_{\text{discharge}}}{1166\text{ mAh }g^{-1}\times m\times w_{\text{S}}}$$

where $m$ and $w_{\text{S}}$ are the weight of the electrode andis the experimentally determined the active material mass fraction in the composite cathode.^[30]^ The listed values correspond to representative reports from the literature, while the data for “This work” are obtained from the present study. The comparison highlights the relative performance of different cathode designs in terms of both high-rate capability and long-term cycling stability.


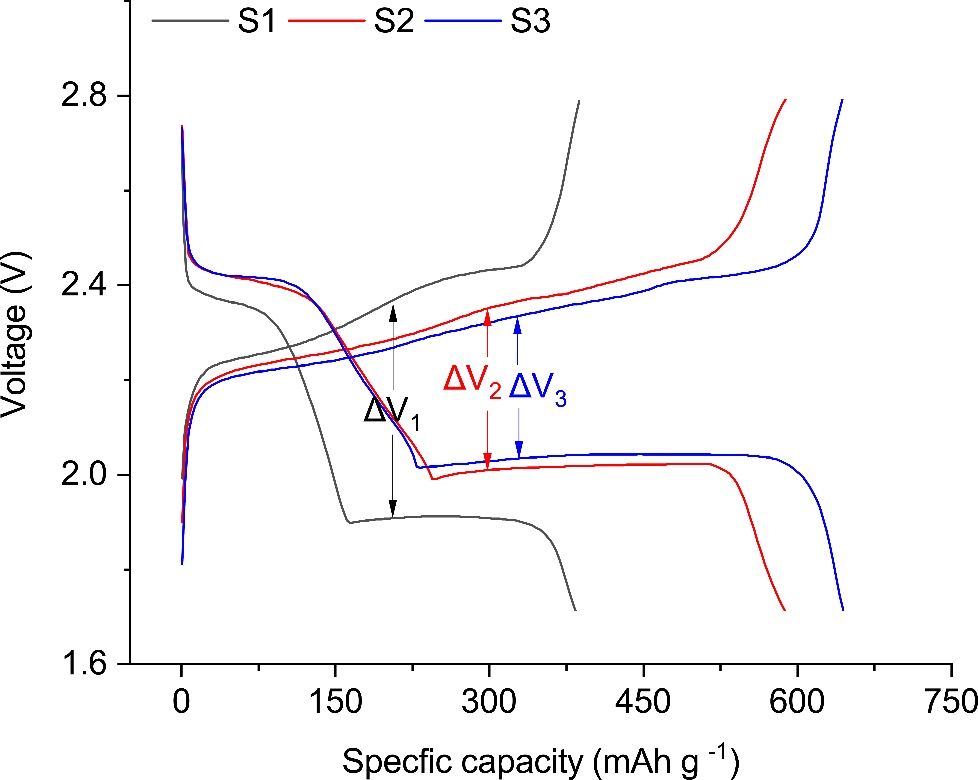


Figure S36. Voltage-capacity profiles of the second cycle for S1, S2, and S3 electrodes at -10 °C under 1 C.

Figure S36 shows the voltage-capacity profiles of the S1, S2, and S3 electrodes at the second cycle under 1 C at -10 °C. Distinct differences are observed in both discharge capacity and polarization. The S3 electrode delivers the highest specific capacity of 644 mAh g^-1^ with a voltage gap (ΔV_3_) of 0.30 V and a capacity ratio (Q_H_/Q_L_) of 1.82. The S2 electrode achieves 589 mAh g^-1^ with ΔV_2_ of 0.34 V and Q_H_/Q_L_ of 1.41. In contrast, the S1 electrode exhibits a lower capacity of 388 mAh g^-1^ with a larger polarization (ΔV_1_ = 0.45 V) and Q_H_/Q_L_ of 1.38. These results demonstrate that the electrode design strongly influences low-temperature electrochemical performance, with S3 showing reduced polarization and improved sulfur utilization compared to S1 and S2.


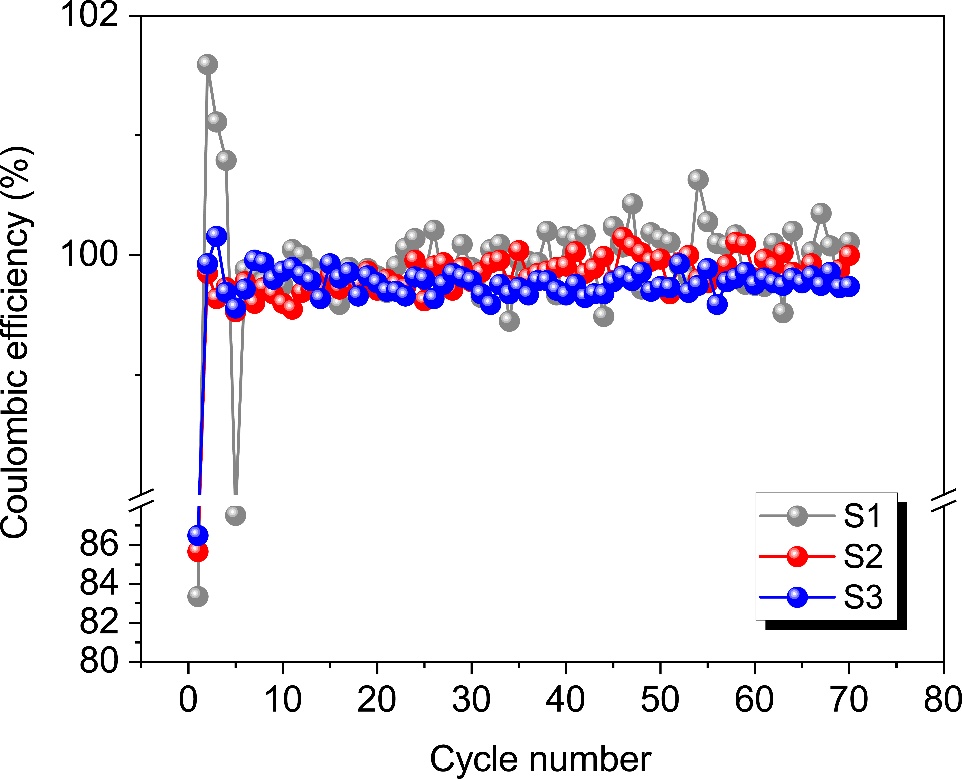


Figure S37. The CE of the S1, S2, and S3 electrodes under low-temperature (-10 °C) cycling conditions.

Under low-temperature (-10 °C) conditions, all electrodes experience a reduction in Coulombic efficiency due to suppressed reaction kinetics and increased internal resistance. The CE profiles of full cells constructed with S1, S2, and S3 cathodes under low-temperature (-10 °C) are shown over 70 cycles. The S3-based full cell exhibits highly stable CE with minimal fluctuations, reflecting uniform reaction behavior and effective suppression of side reactions. In contrast, the S1- and S2-based cells display noticeable spikes and drops, likely arising from less favorable interfacial kinetics or transient LiPSs accumulation. These results highlight the superior structural and catalytic advantages of the dual-gradient S3 electrode in maintaining electrochemical stability at low temperatures.


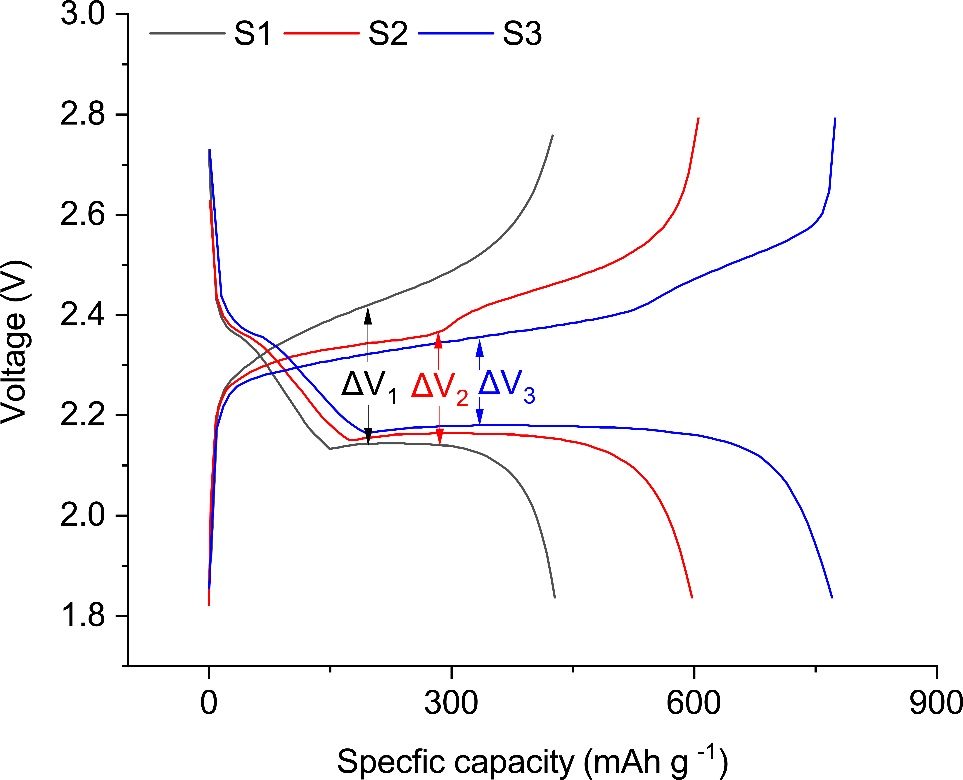


Figure S38. Voltage-capacity profiles of the second cycle for S1, S2, and S3 electrodes paired with a Si/C anode in full cells under 3 C.

Figure S38 compares the voltage-capacity profiles of the S1, S2, and S3 electrodes at the second cycle when paired with a Si/C anode in full cells operated under 3 C. The S3 electrode delivers the highest specific capacity of 774 mAh g^-1^ with the smallest polarization (ΔV_3_ = 0.18 V) and the largest capacity ratio (Q_H_/Q_L_ = 2.78). The S2 electrode shows a specific capacity of 598 mAh g^-1^, ΔV_2_ = 0.22 V, and Q_H_/Q_L_ = 2.46. In contrast, the S1 electrode provides a lower capacity of 429 mAh g^-1^ with larger polarization (ΔV_1_ = 0.29 V) and Q_H_/Q_L_ = 1.88. These results indicate that the S3 configuration enables both improved sulfur utilization and suppressed polarization compared to S1 and S2 under high-rate full-cell conditions.


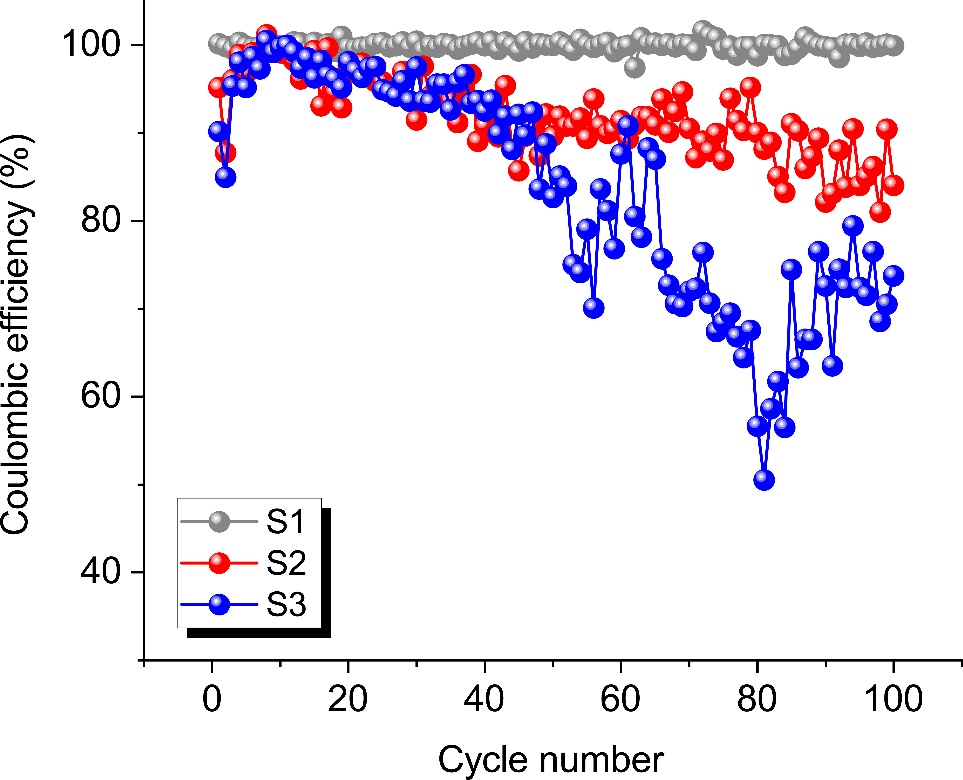


Figure S39. CE of Li_2_S||Si/C full cells assembled with S1, S2, and S3 cathodes over 100 cycles.

The S3 electrode maintains an average CE of 99.89% over 100 cycles, demonstrating excellent redox reversibility and effective suppression of the polysulfide shuttle even under thermally challenging environments. The S2 electrode shows an average CE of 91.90% with noticeable fluctuations and a gradual decline in efficiency, while the S1 electrode suffers from severe CE degradation, dropping below 90% after 40 cycles with an average CE of 83.63%. These results highlight the superior structural and catalytic advantages of the dual-gradient S3 electrode in maintaining electrochemical stability with limited Li source.

| Li metal coated Cu foil | 0.053 g |
| --- | --- |
| Electrolyte (E/S=7.2) | 0.368 g |
| Separator | 0.006 g |
| Al current collector | 0.013 g |
| Cathode active layer | 0.15 g |
| Discharge capacity | 237.16 mAh |

Table S4. The parameters used for specific energy calculation in Figure 6c.

As summarized in Table S4, all parameters used for the pouch-cell energy-density calculation are listed. The calculation includes the masses of the Li-metal-coated Cu foil (anode), electrolyte (E/S = 7.2), separator, Al current collector, and cathode active layer. Based on the measured discharge capacity of 237.16 mAh at 0.05 C, the corresponding specific energy is calculated to be 403 Wh kg^-1^.

**Supplementary References:**

[1] Y. Zhang, H. W. Song, K. R. Crompton, X. Yang, K. Zhao, S. Lee, *Nano Energy* **2023**, 115, 108756.

[2] G. Kresse, J. Hafner, *Physical Review B* **1993**, 47, 558.

[3] P. E. Blöchl, *Physical Review B* **1994**, 50, 17953.

[4] J. P. Perdew, K. Burke, M. Ernzerhof, *Physical Review Letters* **1996**, 77, 3865.

[5] L. Ma, Y. Zhang, S. Zhang, L. Wang, C. Zhang, Y. Chen, Q. Wu, L. Chen, L. Zhou, W. Wei, *Advanced Functional Materials* **2023**, 33, 2305788.

[6] Q. Jiang, H. Xu, K. S. Hui, Z. Ye, C. Zha, Z. Lin, M. Zheng, J. Lu, K. N. Hui, *Angewandte Chemie International Edition* **2024**, 63, e202408474.

[7] Z. Wang, W. Huang, H. Wu, Y. Wu, K. Shi, J. Li, W. Zhang, Q. Liu, *Advanced Functional Materials* **2024**, 34, 2409303.

[8] S. Yu, Y. Sun, L. Song, X. Cao, L. Chen, X. An, X. Liu, W. Cai, T. Yao, Y. Song, W. Zhang, *Nano Energy* **2021**, 89, 106414.

[9] W. Zhang, D. Hong, Z. Su, S. Yi, L. Tian, B. Niu, Y. Zhang, D. Long, *Energy Storage Materials* **2022**, 53, 404.

[10] L. Ren, K. Sun, Y. Wang, A. Kumar, J. Liu, X. Lu, Y. Zhao, Q. Zhu, W. Liu, H. Xu, X. Sun, *Advanced Materials* **2024**, 36, 2310547.

[11] J. Guo, H. Jiang, M. Yu, X. Li, Y. Dai, W. Zheng, X. Jiang, G. He, *Chemical Engineering Journal* **2022**, 449, 137777.

[12] J. Zhang, D. Yang, C. Li, Q. Gong, W. Bi, W.-H. Lai, S. Li, Y. Lei, G. Zhou, A. Cabot, G. Wang, *Energy & Environmental Science* **2025**, 18, 7905.

[13] S. Brunauer, P. H. Emmett, E. Teller, *Journal of the American Chemical Society* **1938**, 60, 309.

[14] W. Weppner, R. A. Huggins, *Journal of The Electrochemical Society* **1977**, 124, 1569.

[15] Y. Zhang, C. Kang, W. Zhao, Y. Song, J. Zhu, H. Huo, Y. Ma, C. Du, P. Zuo, S. Lou, G. Yin, *Journal of the American Chemical Society* **2023**, 145, 1728.

[16] X. Ren, Q. Wang, Y. Pu, Q. Sun, W. Sun, L. Lu, *Advanced Materials* **2023**, 35, 2304120.

[17] Y. Chen, Y. Yao, W. Zhao, L. Wang, H. Li, J. Zhang, B. Wang, Y. Jia, R. Zhang, Y. Yu, J. Liu, *Nature Communications* **2023**, 14, 7487.

[18] B. Sun, D. Wang, Y. Jiang, R. Wang, L. Lyu, G. Diao, W. Zhang, H. Pang, *Advanced Materials* **2024**, 36, 2415633.

[19] W. Wang, X. Wang, J. Shan, L. Yue, Z. Shao, L. Chen, D. Lu, Y. Li, *Energy & Environmental Science* **2023**, 16, 2669.

[20] H. Zhang, M. Zhang, R. Liu, T. He, L. Xiang, X. Wu, Z. Piao, Y. Jia, C. Zhang, H. Li, F. Xu, G. Zhou, Y. Mai, *Nature Communications* **2024**, 15, 5451.

[21] Z. Wu, M. Liu, W. He, T. Guo, W. Tong, E. Kan, X. Ouyang, F. Qiao, J. Wang, X. Sun, X. Wang, J. Zhu, A. Coskun, Y. Fu, *Nature Communications* **2024**, 15, 9535.

[22] J. Shen, Z. Liang, T. Gu, Z. Sun, Y. Wu, X. Liu, J. Liu, X. Zhang, J. Liu, L. Shen, M. Zhu, J. Liu, *Energy & Environmental Science* **2024**, 17, 6034.

[23] J. Pu, S. Fan, Z. Shen, J. Yin, Y. Tan, K. Zhang, B. Wu, G. Hong, Y. Yao, *Advanced Functional Materials* **2025**, 35, 2424215.

[24] J. Zhang, Z. Xie, W. Xi, Y. Zhang, R. Wang, Y. Gong, B. He, H. Wang, J. Jin, *Advanced Energy Materials* **2024**, 14, 2401792.

[25] W. Hua, H. Li, Z. Hu, T. You, J. Qie, H. Dong, H. Sun, Y. Li, S. Dai, Q.-H. Yang, K. Chen, *Advanced Energy Materials* **2025**, n/a, 2501963.

[26] M. Hao, X.-G. Xiong, Z. Li, D. Ma, L. Cai, S. Lu, Q. Yue, *Advanced Energy Materials* **2025**, 15, 2501226.

[27] H. Yuan, J. Zheng, G. Lu, L. Zhang, T. Yan, J. Luo, Y. Wang, Y. Liu, T. Guo, Z. Wang, J. Nai, X. Tao, *Advanced Materials* **2024**, 36, 2400639.

[28] Q. Lv, Y. Sun, B. Li, C. Li, Q. Zhang, L. Wang, *Advanced Energy Materials* **2025**, 15, 2403223.

[29] P. Zhang, Y. Yu, K. Li, R. Yang, R. Hou, Y. Li, Y. Wei, M. Cai, G. Shao, P. Zhang, *Advanced Energy Materials* **2025**, n/a, 2501940.

[30] N. Kang, Y. Lin, L. Yang, D. Lu, J. Xiao, Y. Qi, M. Cai, *Nature Communications* **2019**, 10, 4597.
